# Modelling new insecticide-treated bed nets for malaria-vector control: how to strategically manage resistance?

**DOI:** 10.1186/s12936-022-04083-z

**Published:** 2022-03-24

**Authors:** Philip G. Madgwick, Ricardo Kanitz

**Affiliations:** 1 Syngenta, Jealott’s Hill International Research Centre, Bracknell, RG42 6EY UK; 2 Syngenta Crop Protection, Rosentalstrasse 67, 4058 Basel, Switzerland

**Keywords:** Resistance evolution, Modelling, Resistance management, Vector control, ITNs, Insecticides, Bed nets

## Abstract

**Background:**

The program to eradicate malaria is at a critical juncture as a new wave of insecticides for mosquito control enter their final stages of development. Previous insecticides have been deployed one-at-a-time until their utility was compromised, without the strategic management of resistance. Recent investment has led to the near-synchronous development of new insecticides, and with it the current opportunity to build resistance management into mosquito-control methods to maximize the chance of eradicating malaria.

**Methods:**

Here, building on the parameter framework of an existing mathematical model, resistance-management strategies using multiple insecticides are compared to suggest how to deploy combinations of available and new insecticides on bed nets to achieve maximum impact.

**Results:**

Although results support the use of different strategies in different settings, deploying new insecticides ideally together in (or at least as a part of) a mixture is shown to be a robust strategy across most settings.

**Conclusions:**

Substantially building on previous works, alternative solutions for the resistance management of new insecticides to be used in bed nets for malaria vector control are found. The results support a mixture product concept as the most robust way to deploy new insecticides, even if they are mixed with a pyrethroid that has lower effectiveness due to pre-existing resistance. This can help deciding on deployment strategies and policies around the sustainable use of these new anti-malaria tools.

**Supplementary Information:**

The online version contains supplementary material available at 10.1186/s12936-022-04083-z.

## Background

Malaria is a debilitating infectious disease that continues to carry a significant burden on the poorest communities and most vulnerable people around the world; in 2019, 94% of the estimated 229 million cases of malaria across the world were in Africa and 67% of the estimated 409,000 deaths occurred in children aged under 5 years [1]. Malaria is caused by *Plasmodium* parasites that have vector-borne transmission between human hosts via mosquitoes. In Africa, *Plasmodium falciparum* is the most prevalent parasite accounting for 99.7% of the cases of malaria in 2018, which is mostly transmitted by *Anopheles* mosquitoes (especially *Anopheles gambiae* and *Anopheles funestus*) [2]. Over the last two decades there has been significant investment and progress toward eliminating malaria through vector control; between 2000 and 2015, the international financing of malaria interventions has increased 20-fold, resulting in a 40% decrease in the clinical incidence of malaria and averting an estimated 663 million cases of malaria [3]. The greatest contributor accounting for around 68% of this decrease was the mass distribution of insecticide-treated bed nets (ITNs) [3] that both provide a physical barrier that reduces the opportunity for a mosquito to bite a human and an insecticide that kills mosquitoes that attempt to feed on a protected human during their blood-feeding cycle. In contrast to other interventions (of artemisinin-based combination therapy and indoor-residual spray that account for 22% and 10% of the decrease, respectively) [3], ITNs have been successful because they are cheap [4] and can be mass-distributed freely (or at very low cost) to the poorest communities that tend to be the worst affected by malaria [2]. Between 2016 and 2018, over 578 million ITNs were delivered across the world, including to protect at least 40% of the population in the 10 countries in Africa with the highest prevalence of malaria. In total, since the renewed focus on the eradication of malaria from 2004, over 2 billion ITNs have been delivered worldwide [5].

There has been growing concern that the increasing levels of resistance may threaten the efficacy of ITNs (and, indeed, other vector control methods of malaria intervention) [6–8]. Until recently, the World Health Organization (WHO) only recommended a single mode of action for use on ITNs—pyrethroids, in part because pyrethroids themselves are inexpensive and so contribute to the low cost manufacture of ITNs [9]. Although the clinical incidence of malaria continues to decline year-on-year, the rate of decline is slowing [2] and it is predicted that if pyrethroid-resistance becomes widespread then the trend may reverse, as the cases of malaria averted by ITNs could decrease by more than 40% [10, 11]. This is already beginning to happen in some localities, as the annual decline in the clinical incidence of malaria in the 10 countries in Africa with the highest prevalence of malaria has reversed to an increase in both 2017 and 2018 [2]. At present, the consensus from numerous studies is that pyrethroid-ITNs remain a more effective vector control method than untreated bed nets regardless of resistance because vector mortality tends to be higher even in pyrethroid-resistant strains of mosquitoes [12, 13]. However, results are variable across studies, which may reflect geographical variation in the resistance mechanism affording different levels of resistance. Indeed, across Africa, whilst there has been a dramatic rise in the prevalence of resistance over the last two decades, there is also substantial heterogeneity in the genetics of resistance that differs by region [12–14]. If malaria interventions continue unchanged and the most effective forms of resistance continue to spread throughout Africa, there is a realistic threat that mosquito control will fail and, in the return of prevalence of malaria pre-2000, clinical incidence and mortality due to malaria would more than double [6, 15]. Therefore, resistance is a pressing threat to the steps already made toward the eradication of malaria and there is a need for action to be taken now to prevent reversal—let alone any further progress towards eradication.

To-date, the public health approach to combat malaria has been reactive, using the available tools to decrease malaria incidence under a very limited budget that constrains the cost of conceivable interventions [16]. In general, little consideration has been given to resistance management [17]. For example, DDT was used until resistance rendered it ineffective and other insecticides that have subsequently been deployed have had their usage dictated by the balance of short-term economics and efficacy [6]. However, the threat of insecticide resistance has been proactively responded to before the failure of mosquito control by the Bill and Melinda Gates Foundation in their support of the Innovative Vector Control Consortium (IVCC) with the aim of developing new insecticides for mosquito control in collaboration with industrial partners to eradicate malaria by 2040. The initial research and development work focused on long-lasting indoor-residual sprays [18]; the first product that was launched into the market in 2013 was Actellic® 300CS, which was developed in collaboration with Syngenta using the repurposed organophosphate pirimiphos-methyl. The product received full WHO recommendation [19], which was the normative requirement at the time for governments and aid agencies to purchase the product for vector control. A few years later in 2017, the first new long-lasting ITN (Interceptor® G2), which was developed in collaboration with BASF, was launched, combining the existing pyrethroid alpha-cypermethrin and a new pyrazole chlorfenapyr. Whilst three more indoor-residual sprays have been launched in collaboration between the IVCC and Sumitomo and Bayer in recent years, the only other new long-lasting ITN (Royal Guard®) was developed by Disease Control Technologies without assistance from the IVCC and launched in 2019, combining the existing pyrethroid permethrin with the repurposed juvenile hormone analogue pyriproxyfen (that is unusual in primarily affecting mosquito fecundity rather than mortality). These two new long-lasting ITNs have been included on the prequalified list, which is the current normative requirement for governments and aid agencies to purchase them [20, 21]. Additionally, the IVCC reports that four new chemistries are in the final stages of pre-development with industrial partners [18] including a novel strobilurin-like insecticide for use on ITNs that is being developed in collaboration with Syngenta—that is referred to here as Syngenta Compound 1 (SC1) [22, 23]. Both Interceptor® G2 and Royal Guard® pair existing and repurposed chemistries, but there is the potential to use combinations of new insecticides like SC1 within a single ITN or across multiple ITNs in a deployment area. With a diversity of new chemistries being developed in relative synchrony, there is the rare opportunity for foresight in integrating resistance management into the design and/or deployment of ITNs to delay the evolution of resistance to the new insecticides and ensure high levels of mosquito control for a sufficiently long time to provide the greatest chance of eradicating malaria.

Here, building on a parameter framework [17] that has been extensively analysed [24, 25], conceptual resistance-management strategies using multiple insecticides of rotations, mixtures and mosaics are compared to lower and upper benchmarks to assess what strategy (if any) should be taken to delay the evolution of resistance to the new insecticides. The model setup and analysis differ from Levick et al. [17] in identified ways, including: (i) the explicit assessment of complementary measures of strategy success such as population control, (ii) the comparison of a broader range of resistance-management strategies and (iii) the examination of a different range of scenarios that include new parameters. Massive simulations with variable parameters are used to manage uncertainties and provide an expectation of why different resistance-management strategies are favoured. As SC1 is the only new insecticide where relevant properties are known to the authors, special attention is given to what strategy should be taken with SC1, which is likely to differ from other new insecticides in having a target-site that is encoded in the mitochondrial genome, as an inhibitor of the cytochrome-*bc1* complex like azoxystrobin [26]; this affords it special evolutionary properties that are explicitly treated the simulations and analysis here. Results from the simulations are visualized using conditional inference trees to partition the probabilities of strategy success to describe how a given strategy’s success depends on the parameter space. Further, by examination of parts of the parameter space, the potential for different strategies to be best in combination with different partner insecticides (existing pyrethroids *vs* other new chemistries, based on insecticide effectiveness) and geographic locations (variable zoophily based on female exposure) is considered. In discussion, the practicalities of delivering the recommended strategy are considered to make an initial resistance-management recommendation for SC1 and the other new insecticides.

## Methods

A primary problem for the modelling of resistance evolution is the need to reduce a complex reality into a manageable number of parameters, which also necessarily creates a secondary problem in the capacity to obtain data to estimate those parameters. These problems are particularly acute when attempting to predict the evolution of resistance to new (i.e. untested) insecticides in mosquitoes. This study follows the precedent of an existing mathematical model that has been explored using simulations of parameter combinations [17], which has provided the parameter framework for other studies as well [24, 25]. The model is ‘deterministic’ in that it only describes the evolutionary pressure from selection, which is suitable for making comparisons about the relative time to resistance under alternative resistance-management strategies because those strategies are primarily focused on delaying the evolution of resistance by reducing the strength of selection for resistance. The model supposes that there are two insecticides which each have a corresponding resistance locus with a rare resistance allele. Previous analysis has focused on comparing resistance management via mixtures and sequences, where mixtures involve the use of two insecticides until they both fail and sequences involve using one insecticide until it fails before switching to the other until that one fails too. These previous results show that mixtures tend be favoured when the exposure is low and the effectiveness of insecticides is high. Here, the aim is to compare resistance-management strategies for SC1 and other new insecticides for mosquito control, which brings with it additional challenges that require modifications to the methods in Levick et al. [17], that are described in Additional file 1. The key modifications include: exploring resistance alleles with different combinations of modes of inheritance (i.e. nuclear and mitochondrial), expanding the range of parameters under consideration whilst also significantly increasing the number of simulations run, incorporating a model of population size to more directly address population control, and modifying how strategies are compared to make an arguably fairer comparison (especially with the additional strategies that are considered).

### Mathematical description of selection in the model

This section provides a technical description of the fitness model consistent with the parameter framework in [17], but with some modifications (see Additional file 1). The notation for this section is summarized in Table 1. At the start of a simulation of a parameter combination, alleles are assumed to be in linkage equilibrium. The fitness of an individual with particular alleles and sex can be described as a function of model parameters (Tables 2, 3). For a given generation, the fitness model depends upon how many insecticides are in use and at what rate, which depends upon the strategy—although this really reflects a concept of mosquito interaction with an ITN more so than a practical description of ITN design or deployment:

**Table 1:** Parameter framework and model notation

Parameter		Definition	Notation
Population size		Starting population size (and carrying capacity in logistic model)	$N$
Intrinsic birth rate		% population growth rate (in logistic model)	$b$
Intrinsic death rate		% breeding mosquitoes that die into next generation	$d$
Exposure	Female	% female mosquitoes that receive a dose	*x*_♀_
	Male	% male mosquitoes that receive a dose	*x*_♂_
Insecticide effectiveness	Insecticide 1	% dosed mosquitoes that die from insecticide 1	${m}_{1}$
	Insecticide 2	% dosed mosquitoes that die from insecticide 2	${m}_{2}$
Initial frequency	Allele A	Starting frequency of allele A	${f}_{0,A}$
	Allele B	Starting frequency of allele B	${f}_{0,B}$
Resistance restoration	Allele A	% return to baseline fitness with resistance allele A	${r}_{A}$
	Allele B	% return to baseline fitness with resistance allele B	${r}_{B}$
Dominance of resistance restoration	Allele A	% resistance restoration in heterozygote with allele A	${h}_{A}^{r}$
	Allele B	% resistance restoration in heterozygote with allele B	${h}_{B}^{r}$
Resistance cost	Allele A	% non-dosed mosquitoes that die from carrying allele A	${c}_{A}$
	Allele B	% non-dosed mosquitoes that die from carrying allele B	${c}_{B}$
Dominance of resistance cost	Allele A	% resistance cost in heterozygote with allele A	${h}_{A}^{c}$
	Allele B	% resistance cost in heterozygote with allele B	${h}_{B}^{c}$

**Table 2:**
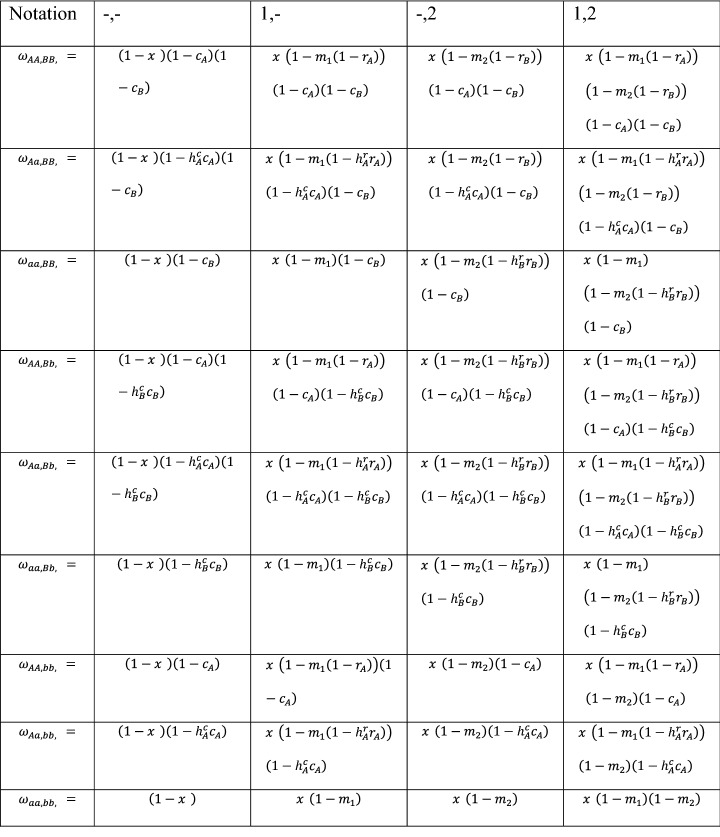
Female fitness separated across insecticide niches, where ‘−‘ represents no insecticide, ‘1’ represents insecticide 1 and ‘2’ represents insecticide 2

Labelling is consistent with Levick et al. [17], except that low concentration insecticide niches are ignored here. If the resistance locus has mitochondrial inheritance, then the homozygotes represent the haploid states

**Table 3:** Male fitness as a function of female fitness

Notation	Expression
*ω*_AA,BB,♂_ =	
*ω*_Aa,BB,♂_ =	
*ω*_aa,BB,♂_ =	
*ω*_AA,Bb,♂_ =	
*ω*_Aa,Bb,♂_ =	
*ω*_aa,Bb,♂_ =	
*ω*_AA,bb,♂_ =	
*ω*_Aa,bb,♂_ =	
*ω*_aa,bb,♂_ =	

If the resistance locus has mitochondrial inheritance, then the homozygotes represent the haploid states

*Sequences* are the simplest ‘strategy’, where one insecticide is 100% in-use at any one point in time and this strategy switches to the second insecticide once the resistance allele to the first in-use insecticide exceeds 50% frequency (i.e. switching from insecticide niche: A, -to- ,B). This corresponds to the default scenario (or ‘non-strategy’) as a ‘benchmark’ for interpreting a delay in resistance from other strategies because an insecticide is used solo on an ITN until its efficacy starts to fail and then a new insecticide is used solo instead. The order in that insecticides are used is determined such that the first-to-break goes first.

*Rotations* are similar, except that the in-use insecticide switches every 36 generations, which corresponds to the recommended three-year replacement or retreatment schedule of ITNs [27]. The first- and second-to-break measures could be crossed multiple times with fluctuating allele frequencies, whereupon the first crossing of the threshold is always recorded. For the most part, the exact length of the replacement or retreatment schedule is essentially arbitrary insofar as switch-period is much smaller than the time to the threshold of resistance (i.e. 50% resistance allele frequency), and this is even more true with the ‘soft’ analysis of the threshold data (see next section). Like sequences, the order of insecticides is with the first-to-break going first.

*Mosaics* involve both insecticides being 50% in-use at the same time (i.e. corresponding to insecticide niches: ½ A,- and ½ -,B), assuming that a mosquito would only encounter one insecticide within a generation. Accordingly, a mosquito has a 50% chance of encountering one or other insecticide within a generation. This is an idealization, but makes for a useful comparison because a micro-mosaic that occurs at such a fine-scale that a mosquito is likely to encounter both insecticides before it reproduces acts like a mixture, or a macro-mosaic that occurs at such a coarse-scale that a mosquito lineage is unlikely to encounter more than one insecticide between generations (because of population structure) acts like solo-use (i.e. sequences).

*Mixtures* involve an exposed mosquito encountering both insecticides at a reduced rate $k$ (i.e. insecticide niche: *k* A,B), as if both insecticides were on the same ITN. $k$ is a constant for a particular parameter combination that ensures that mixtures have the same average initial control as rotations and mosaics for their like-for-like comparison, which is assumed to reflect the balance of formulating each insecticide for use as a mixture with any corresponding modifications of insecticide dose. This constant is calculated as a function of the effectiveness of the two insecticides (${m}_{1}$, ${m}_{2}$; see Table 1):

1 $$k=\frac{{m}_{1}+{m}_{2}-\sqrt{{\left({m}_{1}+{m}_{2}\right)}^{2}-2{m}_{1}{m}_{2}\left({m}_{1}+{m}_{2}\right)}}{2{m}_{1}{m}_{2}}$$

‘*Maximum*’ describes an upper benchmark (like sequences provide a lower benchmark) for the comparison of strategies that are the same as mixtures but assume that $k=1$, which could be plausible were a mosquito to acquire the same dosage of the two insecticides from one or more ITNs within a generation as the mosquito would acquire when each insecticide has solo deployment. This is how mixtures are attributed in Levick et al. [17]. However, here, this is taken to provide an upper benchmark for strategy comparison as an ideal, just like sequences provide a lower benchmark of a non-strategy.

After selection from the exposure to the insecticide niche, breeding takes place using the fitness of individuals with particular alleles (or genotypes) and their sex to generate the frequency of each genotype that offspring will have through random mating. Offspring are assumed to be produced under equal sex ratio. Breeding is carried out by taking the fitness vector of female individuals:2

And the fitness vector of male individuals:3

And taking their cross-product, which produces a matrix that describes the different frequencies of matings between females and males with different genotypes. Each entry of this matrix describes a distribution of offspring genotypes, and so the frequencies of matings are then decomposed, collecting offspring frequency by both genotype and sex. Modelling breeding in this way permits selection to build linkage disequilibrium with respect to the resistance alleles at the two loci and also with respect to their sex.

In the simplest model, offspring frequency by genotype and sex represents the distribution of individuals in the next generation, but this model also considers population size, overlapping generations and density-dependence. Population size and its change is modelled using a simple logistic model using an intrinsic birth rate parameter ($b$). Overlapping generations are included using an intrinsic death rate parameter ($d$) that describes the percentage of breeding adults that survive into the next generation. In mosquitoes, density-dependence is primarily a property of breeding sites rather than feeding sites [28], with competition taking place among larvae that are in the next generation. Consequently, density-dependence is attributed to offspring only, which is attributed such that in the absence of the insecticide(s) the population of females and males (*N*_♀_, *N*_♂_) remains constant at its initial population size ($N/2$ for each sex) as the carrying capacity. Accordingly, with respect to the vector of genotype frequencies for each sex and for adults after selection (*f*_♀,a_, *f*_♂,a_) and offspring (*f*_♀,o_, *f*_♂,o_), the vector of the number of females and males with genotypes in the next generation (, ) is given as:
4a4b

The total number of females in the next generation is simply the sum of the vector of the number of females with genotypes in the next generation () and the same for the total number of males (), as the genotype frequency vector necessarily sums to 1. The vector of genotype frequency by sex can then be decomposed into the total allele frequencies in the population to calculate the measures of strategy success (as appropriate).

### Simulation inputs, scenarios and outputs

The full range of each of the 17 parameters of the genetic model of resistance evolution is explored in a parameter space using the same random sample of 1,000,000 parameter combinations. Several preliminary simulations were run to check that this parameter space and sample size affords a suitable dataset for subsequent analysis. Most parameters are suited to a range between zero and one (Table 4). Some parameters are better suited to a log-scale (population size, initial frequency and resistance cost) to ensure the random sampling of values that would be considered both qualitatively large and small without being implausible. Lastly, one parameter has no meaningful upper limit (intrinsic birth rate) so a standard log-normal distribution is used, which ensures that the majority of randomly sampled values maintain a stable population size (at the carrying capacity) in the absence of insecticides.

**Table 4:** The parameters and their ranges for the deterministic simulations

Parameter		Definition	Range
Population size		starting population size and carrying capacity	10^2^–10^9^
Intrinsic birth rate		% population growth rate (in logistic model)	0–NA
Adult death rate		% breeding mosquitoes that die into next generation	0–1
Exposure	Female	% female mosquitoes that receive a dose	0–1
	Male	% male mosquitoes that receive a dose	0–1
Insecticide effectiveness	Insecticide 1	% dosed mosquitoes that die from insecticide 1	0–1
	Insecticide 2	% dosed mosquitoes that die from insecticide 2	0–1
Initial frequency	Allele A	starting frequency of allele A	10^–9^–10^–2^
	Allele B	starting frequency of allele B	10^–9^–10^–2^
Resistance restoration	Allele A	% return to baseline fitness with resistance allele A	0–1
	Allele B	% return to baseline fitness with resistance allele B	0–1
Dominance of resistance restoration	Allele A	% resistance restoration in heterozygote with allele A	0–1
	Allele B	% resistance restoration in heterozygote with allele B	0–1
Resistance cost	Allele A	% non-dosed mosquitoes that die from carrying allele A	10^–3^–10^−½^
	Allele B	% non-dosed mosquitoes that die from carrying allele B	10^–3^–10^−½^
Dominance of resistance cost	Allele A	% resistance cost in heterozygote with allele A	0–1
	Allele B	% resistance cost in heterozygote with allele B	0–1

Where possible, parameters are randomly sampled across their full range, but some parameters are better suited to a log-scale (population size, initial frequency and resistance cost) and one parameter has no meaningful upper limit (intrinsic birth rate) so a standard log-normal distribution is used (with mean = 0 and sd = 1). Initial frequency is limited to be within the range 1/N to N/100 where N is population size

For each randomly sampled set of the 1,000,000 parameter combinations as the input, the fitness model is run for 15 scenarios from all combinations of the five strategies identified above and three combinations of modes of inheritance for the two resistance loci. With special consideration of SC1, there is a need to incorporate an insecticide where target-site resistance evolves at a mitochondrial locus, which differs from a nuclear locus through maternal inheritance and (effective) haploidy. Although SC1 is known to have a target-site that has mitochondrial inheritance, this does not mean that resistance will only ever evolve at the mitochondrial locus as resistance could evolve through mutations that affect regulatory factors or metabolic pathways that would most likely have nuclear inheritance. Further, other new insecticides may not also have a mitochondrially-inherited target-site. As a result, simulations to assess the evolution of resistance to new insecticides are run for combinations of resistance alleles with different modes of inheritance: nuclear inheritance only (nuclear and nuclear; often abbreviated to NN to describe each locus), mixed inheritance (or mitochondrial and nuclear; MN) and mitochondrial inheritance only (or mitochondrial and mitochondrial, MM).

The main output of the simulations are two measures of resistance evolution: the time for the first resistance allele to reach 50% frequency (first-to-break) and the time for the second resistance allele to reach 50% frequency (second-to-break). This approach is consistent with the majority of genetic models in the literature [29], but is only indirectly related to the goals of resistance management in mosquito control and malaria eradication. The logistic model is used to give an equivalent threshold metric for mosquito control, equivalent to how the time it takes for a resistance allele to reach 50% frequency is a metric for the spread of resistance. Across the scenarios considered, it is important that the chosen threshold is low enough that the female mosquito populations start below it in the first generation that the insecticide is applied, high enough to provide a large quantitative separation between strategies and not-too-high that most female mosquito populations do not reach the threshold within the timeframe that the model is examined across (500 generations). In-keeping with previous work [30], the threshold of the time that it takes for the female population to recover is taken to be 80% of its size prior to application of insecticides to balance these considerations. For the resistance and control metrics, the simulation output will record a value or it will not; when it does not, an additional step of data extraction is undertaken to provide information on why a value is not recorded. Based on the change in allele frequency or population size between the start and end of the simulation, the simulated runs can be categorized into:

*Successful measurement* within 500 generations, as 50% resistance allele frequency or 80% of initial population size are reached.

*Toward the threshold* where there is an increase in allele frequency, implying that successful measurement would occur in > 500 generations.

*Away from the threshold* where there is a decrease in allele frequency, implying that allele-frequency change has followed the opposite direction of resistance evolution.

*Population extinction*, as the female population size drops below one individual leading to extinction of that simulation’s population.

For the purpose of analysis, these simulation-outcome types, or simply “data types”, are given nominal values that ensure their ranked interpretation (from shortest to longest): where ‘Toward Threshold’ is set to 1000, ‘Away from Threshold’ is set to 1500 and ‘Extinction’ is set to 2000.

### Analysis of simulation outputs

Following Levick et al. [17], the primary method of analysis of simulation data uses conditional inference trees. With the comparison of multiple strategies for a given measure (e.g. first-to-break), conditional inference trees provide a robust analysis for the categorical classification of when a particular strategy tends to be favoured in different regions of the parameter space. Trees are built and drawn using R:ctree (in the ‘partykit’ package) [31], which uses iterative permutation tests in an algorithm to make a binary split in the variable with the strongest differentiation of output distributions. The iterations that form the tree have a controlled stop when the algorithm can no longer make a split into terminal nodes with > 5% of the data, which is a control applied for the visualization of the tree to ensure a manageable number of terminal nodes. Necessarily, there is an element of bias introduced by the choice of any parameter space, but the use of conditional inference trees mitigates against wholescale bias because the meaningful outputs do not rely upon the frequency of a category across the chosen parameter space but rather within statistically different parameter subspaces that are identified algorithmically. Therefore, whilst the frequency of categories must be interpreted with caution (as they depend on the chosen parameter space), there is only the potential for bias to enter into the output in determining the precise boundaries between parameter subspaces where different strategies are more successful. In this way, the use of conditional inference trees is aligned with the aim of understanding how some strategies are favoured over others for a particular measure, which can then provide the basis for insight into why some strategies are favoured over others in different contexts.

For building conditional inference trees, the classification can include ‘sequences’ as a lower benchmark, but must exclude the ‘maximum’ upper benchmark because this would mask meaningful comparisons, so comparisons are made between sequences, rotations, mosaics and mixtures. Recognizing that the development of mixtures of new insecticides has significant challenges (e.g. physio-chemical compatibility), the classification of which strategy is favoured is also rerun excluding mixtures (i.e. for sequences, rotations and mosaics) to consider how this alters the results. Taking one measure at a time (first-to-break, second-to-break or control-failure), the output variable is assembled by classifying which one or combination of strategies have > 10% difference (in either direction) for that measure. A combination of strategies is categorized when strategies have < 10% difference with each other and all have > 10% difference with all other strategies. This ‘soft’ cut-off can help avoid misinterpreting the quantitative variation in the measure where strategies have near-equal results. However, this does generate a problem of intransitivity (e.g. where A is near-equal B, B is near-equal C and A is not near-equal C), which is best avoided by making fewer comparisons. Consequently, although results can be summarized in a tree that compares all strategies and their combinations at once (as is done in the main-text), these results are compared to a different set of trees where each strategy is examined in isolation and classified as the most successful (> 10% difference than all others), the equally-most successful with one or more other strategy (> 10% difference than the worst), the equally-most successful alongside all other strategies (all < 10% difference) or not among the most successful (other(s) have > 10% difference; Additional file 2 and Additional file 3: Figures S1–12).

Whilst conditional inference trees describe how different strategies are favoured in particular parameter spaces, here there is a special focus in the resistance-management strategy for SC1 and other new insecticides. As such, the secondary method of analysis concerns the results when a new insecticide is used alongside a partner insecticide and in different geographic settings. Due to the uncertainty around the genetic mechanism of resistance for SC1 or any other new insecticide, the only parameter that SC1 or another new insecticide constrains is effectiveness because of the design constraint from WHO criteria, requiring that a new insecticide has > 0.8 effectiveness to obtain prequalification listing [21, 27]. A new partner insecticide would be similarly constrained, but the WHO also recommends the use of pyrethroids despite widespread resistance [9] that tend to have < 0.8 effectiveness [12, 13]. Yet, regardless of the insecticide, the genetics of new forms of resistance is uncertain. Further, in different geographic settings, there are many complex and interacting factors that contribute toward uncertainty in exposure, including zoophily [32] and coverage [2]. In response to these uncertainties when seeking to assess the scope for resistance management with at least one new insecticide, the impact of effectiveness can be assessed by exploring the trend in time to resistance across effectiveness to capture variation in insecticide choice and exposure to capture variation in geographic setting.

## Results

A parameter space of 17 input parameters was assembled by randomly sampling the parameters for each of the 1 million simulated runs of the model for specific combinations of resistance allele modes of inheritance (N = nuclear and M = mitochondrial, giving NN, MN and MM combinations) and strategies (the lower benchmark sequences, the upper benchmark maximum and the three main strategies: rotations, mosaics and mixtures) to record data on the time for the first and second resistance allele to reach > 50% frequency (first-to-break and second-to-break), the time for the population to recover to > 80% of its size prior to the application of insecticides (control-failure) and the data types (especially the occurrence of extinction). To avoid any ambiguity, the *same* random sample of parameters was used for each of the 15 simulations of combinations of inheritance and strategy to make results comparable by inheritance and strategy. The total number of simulations run here is therefore 15 million.

### Distributions of the data types

**Fig. 1:**
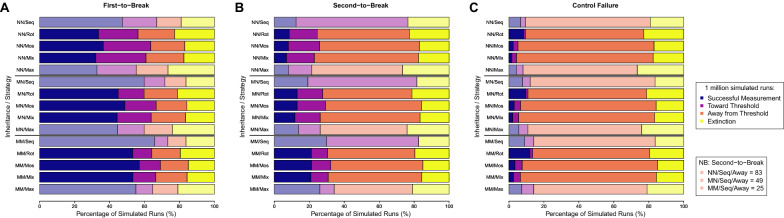
Bar chart of the output of the simulation of 1 million randomly sampled parameter combinations by data type. Simulations are divided between threshold measures (**A**–**C**), mode of inheritance (N = nuclear and M = mitochondrial, giving NN, MN and MM combinations) and strategy (Seq = sequences, lower benchmark; Rot = rotations; Mos = mosaics, Mix = mixtures; Max = maximum, upper benchmark). Simulation runs are classified as ‘Successful Measurement’ meaning that the threshold statistic was exceeded to give a time to the threshold measurement, ‘Toward Threshold’ meaning that the resistance allele or population size was approaching the threshold but too slowly to give a measurement, ‘Away from Threshold’ meaning that resistance allele or population size was decreasing over time (or, for population size, never decreases below 80% to be able to give a measurement) and ‘Extinction’ meaning that the female population size drops below 1 (and the simulation terminates). For **B** and the second-to-break measure, the bar chart appears to show no ‘Away from Threshold’ data types for the sequences strategy, but this is due to a low number of simulated runs (with raw numbers given in the NB box), which reflects that the insecticide that is first-to-break has a resistance allele that is placed first in the sequence order

The results show substantial variability in the simulation-outcome data types across different inheritances, strategies and measures (Fig. 1). For all measures (i.e. ‘first-to-break’, ‘second-to-break’ and ‘control failure’), there is an almost linear trend in data types across inheritances, with nuclear-only inheritance (NN) showing fewer successful measurements and more changes in allele frequency toward the 50% threshold for measurement, mitochondrial-only inheritance (MM) showing a shift toward the opposite pattern and mixed inheritances (MN) showing an intermediate pattern. With mixed modes of inheritance (MN), the insecticide with the resistance allele with mitochondrial inheritance is disproportionately more likely to be the first-to-break (with a mean across strategies of 61.5%). Across modes of inheritance, the first-to-break measure shows more successful measurements and changes in allele frequency toward the threshold for measurement than the second-to-break measure that shows many more changes in allele frequency away from the threshold for measurement, which reflects the inherent order in the results of these measures (i.e. naturally the second-to-break would take longer than the first-to-break event).

Across comparisons, strategies group together with similar distributions of data types. The two strategies with a temporal dimension as insecticide use switches through time (sequences benchmark and rotations) tend to group together, although sequences show an elevated frequency of successful measurements for the first-to-break measure due to applying the strongest selection pressure of any strategy (from one insecticide in constant use) and a highly elevated frequency of changes in allele frequency toward (at the expense of away from) the threshold for measurement for the second-to-break measure because the resistance allele starts from its initial frequency at the point that its insecticide first comes into use (which is a constraint of the use of this benchmark). The other three strategies without the temporal dimension (mosaics, mixtures, maximum) also tend to be grouped together, but the maximum strategy shows an elevated frequency of population extinctions due to applying the strongest initial control of any strategy (from two insecticides at full combined effectiveness).

Both the second-to-break and control-failure measures show a large fraction of simulated runs with changes in allele frequency away from the threshold for measurement; this has consequences for strategy comparison, implying that strategies have a more uniform impact on these measures than first-to-break. This is especially problematic for the control-failure measure, which has a large fraction of simulated runs with changes in allele frequency away from the threshold for measurement because the population size never drops below the threshold of 80% of initial population size for the recording of a measure of returning to 80% of population size. Altering the 80% threshold does not lead to the generation of more data where strategies can be differentiated because such data would simply reveal more space where strategies have a more uniform impact of this measure. The main driver of this result is that the simulation across parameter ranges covers a large fraction of parameter combinations where the initial control that decreases population size is minimal relative to the intrinsic birth rate that increases population size. This is a consequence of the unbiased approach to sampling the parameter space. Furthermore, simply decreasing the average intrinsic birth rate does not provide a solution because this causes a reversion to another problematic outcome in decreasing the frequency of successful measurement of the first- and second-to-break measures through increasing the frequency of extinctions. But, moreover, making a change would imply that the frequency of this data type result is simply an artefact of the model setup, whereas it is an informative result in suggesting that strategies have a more uniform impact on control, which is unsurprising given that all the strategies have similar initial control (and, to make strategy comparison fairer, mixtures are even ensured to have the exact same average initial control as rotations and mosaics). Therefore, whilst this data type means that the control-failure measure is not informative for analysis going forward (although the results of the analysis of this measure are presented in Additional file 3), the focus of analysis can now shift to where strategies produce a different result, which is in their first-to-break, second-to-break and probability-of-extinction measures (derived from data types that reflect population control).

**Fig. 2:**
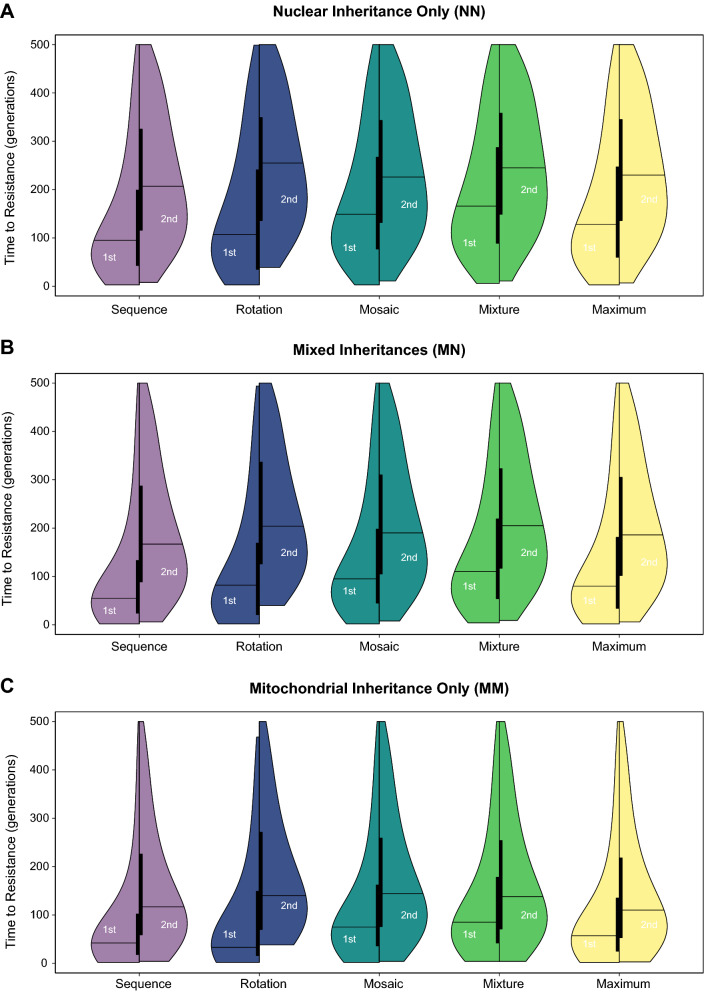
Violin plot of the time to resistance allele exceeding 50% frequency. The distribution is plotted for the ‘Successful Measurement’ data type (see Fig. 1) with results divided between panels (**A**–**C**) by the mode of inheritance (N = nuclear and M = mitochondrial, giving NN, MN and MM combinations). Strategies are plotted purple-to-yellow (R:viridis) by colour with the benchmarks (Sequences and Maximum) given a faded colour. Violins are drawn using R:vioplot with separate lefts for the first-to-break measure (1st) and rights for the second-to-break measure (2nd). Each half-violin has the kernel-density distribution around a box plot, where the vertical bar is drawn between the upper (75th percentile) and lower (25th percentile) quartiles and the horizontal bar is at the median

Within the data type of ‘successful measurement’ within 500 generations for the first- and second-to-break measures (Fig. 2), there is a consistent distribution of measured values across inheritance and strategy. Whilst it is rare for a measure to be recorded in a very small number of generations, which reflects the time it takes for a resistance allele to spread from its initial frequency (that can be as high as 1%) to > 50% frequency, it is also rare for a measure to take a large number of generations (nearer 500), which reflects that the distribution is conditional upon successful measurement within 500 generations. The result is a skewed-normal-like distribution, which is more skewed toward zero for the first-to-break measure than the second-to-break measure because of the inherent order in the results of these measures. Following the same pattern that is observed in the distribution of data types, inheritance produces an almost linear trend across inheritance in both measures, with mitochondrial-only inheritance (MM) tending to show a faster time to resistance for both measures, nuclear-only inheritance (NN) tending to show a slower time to resistance and mixed inheritances (MN) showing an intermediate pattern between the two extremes. Across strategies, sequences appear to provide a suitable lower benchmark in having a distribution that is obviously lower for both measures in being more skewed toward zero, but the maximum strategy does not appear to obviously set an upper benchmark. This can be explained with reference to data types (Fig. 1), where maximum shows an elevated frequency of extinctions, which are excluded from the distribution of the data type for successful measurement (Fig. 2). This implies that the distribution of successful measurements shows an underrepresentation of longer times to resistance for the maximum because of extinction. This finding demonstrates that the analysis going forward needs to incorporate multiple data types to accurately describe the results. Across the main strategies, the degree of skew in the distribution toward zero of the first-to-break measure follows the order (more-to-less) of rotations, mosaics and then mixtures, but the distributions are more similar (and variable across inheritance) for the second-to-break measure.

### Conditional inference trees

**Fig. 3:**
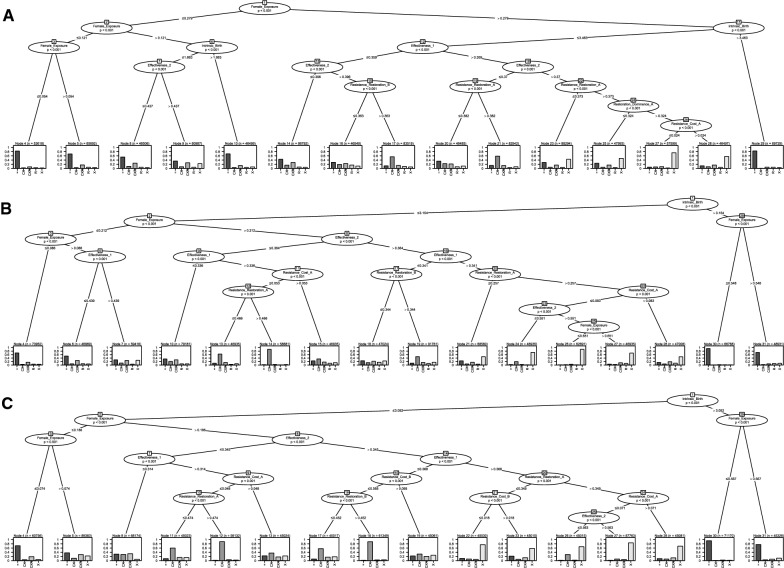
Conditional inference trees for the first-to-break measure by each combinatorial mode of inheritance (N = nuclear and M = mitochondrial, giving combinations for **A** as NN, **B** as MN and **C** as MM). The simulation data on the first-to-break for each strategy is classified into a categorical variable to describe whether one or multiple strategies have > 10% difference (in either direction) in their first-to-break measure, including the ‘sequences’ lower benchmark but excluding the ‘maximum’ upper benchmark (because this would mask meaningful comparisons). Non-measured data types (see Fig. 1) are given nominal values that ensure their hierarchical interpretation: where ‘Toward Threshold’ is set to 1000, ‘Away from Threshold’ is set to 1500 and ‘Extinction’ is set to 2000. Data classifications are given for all strategies and their combinations, but only five classifications are needed to describe the data: ‘ = ’ where all strategies have < 10% difference, ‘CX’ where mosaics (C) and mixtures (X) have < 10% difference but > 10% difference than rotations (R) or sequences, ‘CXR’ where mosaics (C), mixtures (X) and rotations (R) have < 10% difference but > 10% difference than sequences, ‘R’ where rotations have > 10% difference than all other strategies and ‘X’ where mixtures have > 10% difference than all other strategies. The conditional inference tree is used to partition the data classification output based on the parameter space inputs based on the 1 million randomly sampled parameter combinations for the 17 parameters (see Table 4). Trees are built and drawn using R:ctree, which uses permutation tests to iterate an algorithm that tests the independence between the inputs and output variables and makes a binary split in the variable with the strongest differentiation of output distributions. The parameter of each split is given in the nodes within the tree, which reports the parameter (as per Table 4) and the *p*-value of the independence test; the quantitative place of the split in the parameter itself is recorded in the line between nodes. The iterations that form the tree stop when algorithm can no longer make a split into terminal nodes with > 5% of the data, which is a control applied for the visualization of the tree to ensure a manageable number of terminal nodes. The distributions of data classification are given in the terminal nodes as a bar chart, where the y-axis describes the proportion of data points

Conditional inference trees provide a robust analysis for describing how the quantitative value of a particular parameter influences whether or not a particular strategy tends to be favoured over others. For a given measure, a strategy is favoured when it has > 10% difference (in either direction) for that measure, which also applies to a combination of strategies when a subset of strategies has < 10% difference with each other and all have > 10% difference than all other strategies. All data types are included in the analysis using nominal values. For the conditional inference tree comparing the four strategies (sequences, rotations, mosaics and mixtures; excluding maximum as this would mask the other strategies) and their combinations as presented here in the main text, there is potentially a problem of intransitive comparisons. This can be avoided by making a more explicit comparison of when a particular strategy is favoured outright, in combination with some strategies against others, equally with all other strategies or is not favoured. This approach requires generating one conditional inference tree per inheritance and strategy as is presented in Additional file 3: Figures S1–12. Yet, the categorization process reveals that only five classifications are needed to describe the data: ‘ = ’ where all strategies have < 10% difference, ‘CX’ where mosaics (C) and mixtures (X) have < 10% difference but > 10% difference than rotations (R) or sequences, ‘CXR’ where mosaics (C), mixtures (X) and rotations (R) have < 10% difference but > 10% difference than sequences, ‘R’ where rotations have > 10% difference than all other strategies and ‘X’ where mixtures have > 10% difference than all other strategies. Consequently, with just five comparisons, the results for the summary tree of all comparisons presented here are a good approximation of the cumulative results of the trees-by-strategy for a given inheritance (Fig. 3). Additionally, the summary of results presented here is only for the first-to-break measure, but the second-to-break and control-failure measures are detailed in summary and full figures in Additional file 3: Figures S13–42.

Across the conditional inference trees for the different modes of inheritance, the results are remarkably consistent for the first-to-break measure. All strategies tend to be near-equally favoured ( =) when female exposure is < 0.3, which is logical because this would most likely represent a scenario where the balance of the conditional advantage to a female from resistance restoration under exposure to an insecticide is offset by the unconditional disadvantage of the resistance cost. Indeed, this explanation can be confirmed as a large percentage of simulated runs (means: NN = 70.8%, MN = 61.2%, MM = 53.4%) show that the change in allele frequency is either toward or away from the threshold when female exposure is < 0.3. Additionally, all strategies tend to be near-equally favoured (=) when female exposure is > 0.3 and the intrinsic birth rate is > 3, which corresponds to an extremely high birth rate with unstable population dynamics such that a large percentage of simulated runs end in extinction (means: NN = 70.5%, MN = 71.2%, MM = 73.9%). Mixtures (X) tend to be favoured outright when female exposure is > 0.3, the intrinsic birth rate is < 3 and the effectiveness of both insecticides is > 0.35. When one or other insecticide has effectiveness > 0.35, then mixtures tend to be favoured alongside mosaics (CX) or mosaics and rotations (CXR, which is all the strategies apart from the sequences benchmark). In this parameter space, mixtures and mosaics tend to be favoured (CX) when the insecticide with higher effectiveness (> 0.35) also has higher resistance restoration (> 0.4). The second-to-break measure also shows a related pattern across inheritances, all strategies tend to be near-equally favoured (=) when the intrinsic birth rate is > 3, where the extremely high birth rate leads to unstable population dynamics that often end in extinction (means: NN = 78.2%, MN = 79.2%, MM = 80.0%). Alternatively, when the intrinsic birth rate is < 3, mosaics, mixtures and rotations (CXR) are favoured over sequences. In contrast to both the time to resistance measures, the control-failure measure shows no detectable pattern across the parameter space, which is simply because all strategies are near-equally favoured (=).

In case mixtures are not practically possible, the classification can be rerun excluding mixtures (i.e. for sequences, rotations and mosaics) to consider how this alters the conditional inference tree results (see Additional file 3: Figures S43–78). In short, where mixtures would have been favoured outright (X), instead mosaics tend to be favoured outright (C). For the first-to-break measure, all strategies tend to be near-equally favoured (=) when the female exposure < 0.3 or female exposure is > 0.3 and the intrinsic birth rate is > 3.5. When female exposure is < 0.3 and the intrinsic birth rate is < 3.5, mosaics tend to be favoured outright (C) when the effectiveness of at least one insecticide is > 0.45, else mosaics and rotations are jointly favoured (CR).

### Scenarios of new insecticides

To provide a quantitative estimation of the difference between strategies across the relevant parameter space for the possibilities for the deployment of new insecticides, the broad trends in the parameter space for all of the different strategies including both benchmarks can be examined across key parameters. The choice of a new insecticide and its partner restricts the parameter space along the axes of the effectiveness of both insecticides, where a new insecticide (including SC1) has high effectiveness > 0.8 from WHO guidelines [27] whereas pyrethroids can have variable effectiveness within and between localities [12–14]. Different geographic regions may have different genetic mechanisms of resistance, but this is unpredictable and so geographic variation is only interpreted as restricting the parameter space along the axes of female exposure that is primarily understood as reflecting mosquito zoophily and/or ITN coverage. Consistent with conditional inference trees, there is little distinction between strategies for the second-to-break and control-failure measures (Additional file 3: Figures S13–42), and so results presented here in the main-text focus on the probability of resistance and time to the first-to-break. The probability of resistance describes the fraction of results of the data types for the first-to-break measure that are either ‘Successful Measurement’ or ‘Toward Threshold’ where allele frequencies are changing in the direction of resistance.

Across modes of inheritance, nuclear-only (NN) and mitochondrial-only (MM) inheritances show a similar variability in the probability of resistance and time to first-to-break across the parameters of effectiveness and female exposure, but with mitochondrial-only inheritance (MM) showing a greater probability of resistance and shorter time to first-to-break. Like for the conditional inference trees, mixed inheritance (MN) shows an intermediate pattern across effectiveness parameters, which is closer to nuclear-only inheritance (NN) with lower values of effectiveness and closer to mitochondrial-only inheritance (MM) with higher values of effectiveness. This is a consequence of the insecticide with the resistance allele with mitochondrial inheritance being more likely to be the first-to-break when the corresponding resistance allele has higher effectiveness. In contrast, mixed inheritance (MN) has a pattern across female exposure that is much closer to mitochondrial-only inheritance (MM), which reflects the high probability (with a mean across strategies of 69.0%) that the insecticide with the mitochondrial resistance allele is also the first-to-break.

**Fig. 4:**
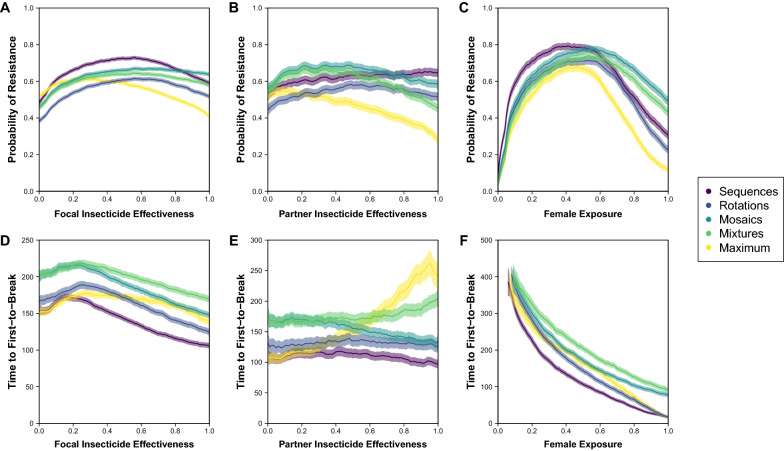
Relationship between insecticide choice and geographic location on probability of resistance and time to first-to-break with nuclear-only inheritance (NN). Insecticides differ by their effectiveness and geographies differ by their female exposure. The probability of resistance describes the fraction of all simulated runs where the data type is ‘Successful Measurement’ or ‘Toward Threshold’ (see Fig. 1). The time to first-to-break is calculated from the ‘Successful Measurement’ data type only. In each panel, the bold-colour lines (per strategy purple-to-yellow; R:viridis) come from partitioning the y-axis parameter from the simulations by the x-axis parameter into 101 rounded bins and calculating the mean of the y-axis measure per bin; a $k=5$ backward-tail moving average is used to smooth the mean-line. Around each bold-colour line, there is a transparent-shading of the same colour that describes the 95% confidence intervals for the mean ($\pm 1.96*SE$), which is also smoothed with a $k=5$ backward-tail moving average. In panels A and D, the x-axis is arbitrarily designated for a focal insecticide as effectiveness 1, as if it were a new insecticide. In panels B and E, effectiveness 1 is assumed to be > 0.8 (in accordance with WHO guidelines for new ITNs), and the x-axis is then for a partner insecticide. In panels C and F, effectiveness 1 is also assumed to be > 0.8, and the x-axis is then for fem ale exposure

**Fig. 5:**
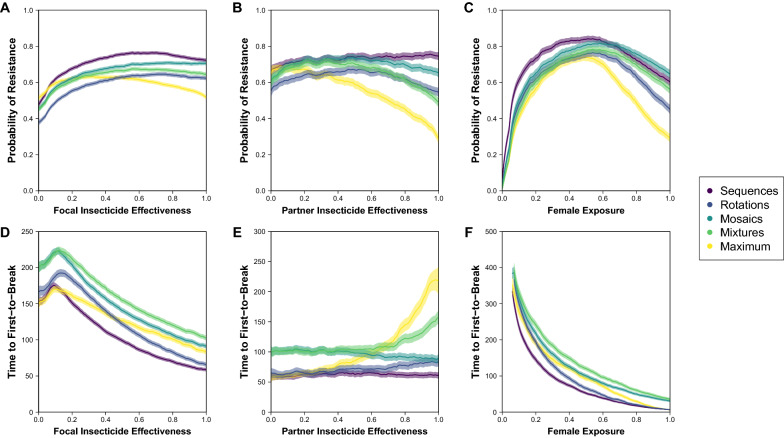
Relationship between insecticide choice and geographic location on probability of resistance and time to first-to-break with mixed inheritance (MN). Insecticides differ by their effectiveness and geographies differ by their female exposure. The probability of resistance describes the fraction of all simulated runs where the data type is ‘Successful Measurement’ or ‘Toward Threshold’ (see Fig. 1). The time to first-to-break is calculated from the ‘Successful Measurement’ data type only. In each panel, the bold-colour lines (per strategy purple-to-yellow; R:viridis) come from partitioning the y-axis parameter from the simulations by the x-axis parameter into 101 rounded bins and calculating the mean of the y-axis measure per bin; a $k=5$ backward-tail moving average is used to smooth the mean-line. Around each bold-colour line, there is a transparent-shading of the same colour that describes the 95% confidence intervals for the mean ($\pm 1.96*SE$), which is also smoothed with a $k=5$ backward-tail moving average. In **A**, **D**, the x-axis is designated for the insecticide that corresponds to the mitochondrial inheritance of resistance as effectiveness 1, as if it were a new insecticide. In **B**, **E**, effectiveness 1 is assumed to be > 0.8 (in accordance with WHO guidelines for new ITNs), and the x-axis is then for a partner insecticide. In **C**, **F**, effectiveness 1 is also assumed to be > 0.8, and the x-axis is then for female exposure

**Fig. 6:**
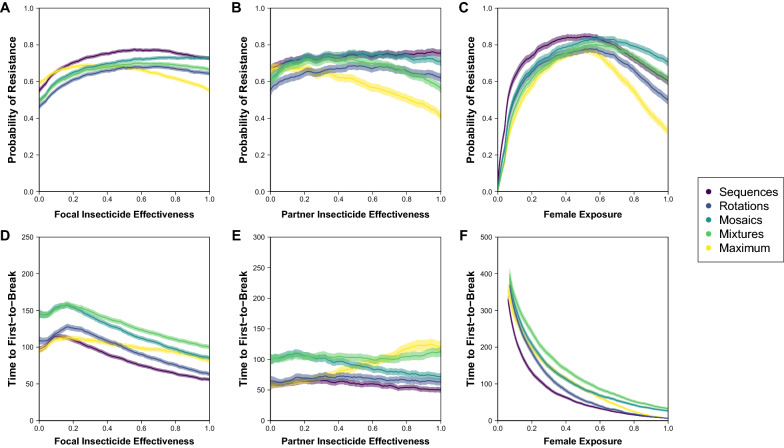
Relationship between insecticide choice and geographic location on probability of resistance and time to first-to-break with mitochondrial-only inheritance (MM). Insecticides differ by their effectiveness and geographies differ by their female exposure. The probability of resistance describes the fraction of all simulated runs where the data type is ‘Successful Measurement’ or ‘Toward Threshold’ (see Fig. 1). The time to first-to-break is calculated from the ‘Successful Measurement’ data type only. In each panel, the bold-colour lines (per strategy purple-to-yellow; R:viridis) come from partitioning the y-axis parameter from the simulations by the x-axis parameter into 101 rounded bins and calculating the mean of the y-axis measure per bin; a $k=5$ backward-tail moving average is used to smooth the mean-line. Around each bold-colour line, there is a transparent-shading of the same colour that describes the 95% confidence intervals for the mean ($\pm 1.96*SE$), which is also smoothed with a $k=5$ backward-tail moving average. In **A**, **D**, the x-axis is arbitrarily designated for a focal insecticide as effectiveness 1, as if it were a new insecticide. In **B** and **E**, effectiveness 1 is assumed to be > 0.8 (in accordance with WHO guidelines for new ITNs), and the x-axis is then for a partner insecticide. In **C** and **F**, effectiveness 1 is also assumed to be > 0.8, and the x-axis is then for female exposure

Across strategies, the same patterns that were observed in the conditional inference trees can be given a rough quantitative estimation (Figs. 4, 5, 6). Again, there is a division in the shape of results among strategies based on the temporal dimension of insecticide switching through time, with sequences and rotations showing a similar variability in the probability of resistance and time to first-to-break across the parameters of effectiveness and female exposure, and mosaics, mixtures and maximum showing a similar variability (albeit that mosaics show the opposite trend across partner insecticide effectiveness for the time to first-to-break). Across all strategies and the probability of resistance and time to first-to-break (panels A, D), there is a similar pattern of results across focal insecticide effectiveness with a higher probability of resistance at intermediate effectiveness, which reflects the balance of data types (Fig. 1) at the extremes of ‘Away from Threshold’ with lower effectiveness < 0.3 and ‘Extinction’ with higher effectiveness > 0.5. In comparison between strategies, mosaics and mixtures tend to perform similarly with lower effectiveness < 0.3, but with mosaics having a higher probability of resistance and shorter time to first-to-break with higher focal insecticide effectiveness. Rotations have a lower probability of resistance and a shorter time to first-to-break than mosaics and mixtures. The quantitative differences between strategies do not matter for directly addressing the question using a strategy to delay the evolution of resistance because WHO guidelines [27] require that an insecticide has > 0.8 effectiveness, but this does indicate what this guideline entails.

There is less variability across the trend line of partner insecticide effectiveness and more variability in estimating the trend line (panels B, E), which is partly because the parameter space is restricted to assume a new focal insecticide with effectiveness > 0.8. Sequences and rotations are especially uniform across partner insecticide effectiveness, with rotations increasing the time to first-to-break compared to the sequences benchmark (that is like a non-strategy negative control) by ~ 25%, which is roughly equivalent to an additional two years of insecticide susceptibility from a baseline of eight years. Again, mosaics and mixtures tend to perform similarly with low effectiveness < 0.3, which increases the time to first-to-break compared to the sequences benchmark by ~ 50% or an additional four years of insecticide susceptibility. With higher partner insecticide effectiveness, mosaics show a decrease in the time to first-to-break to become very similar to rotations, whereas mixtures show an increase in the time to first-to-break of up to ~ 100% or an additional eight years of insecticide susceptibility. Interestingly, the maximum benchmark, which is how mixtures are attributed in [17], performs similarly to sequences with low partner effectiveness < 0.3, which is because the time to first-to-break solely reflects the focal insecticide effectiveness; but increases with partner insecticide effectiveness to delay resistance by up to 150% or an additional twelve years on the sequences benchmark.

Whilst there is more variability across female exposure (panels C, F), strategies are more uniform. Sequences both show a higher probability of resistance and lower time to first-to-break than other strategies with lower female exposures, but all other strategies are similar. At higher female exposures, the main strategies have quantitatively different probabilities of resistance that follow a predictable order with mosaics having the highest probability of resistance, mixtures next highest and then rotations; but the difference between strategies narrows with more resistance alleles with mitochondrial inheritance. At higher female exposures for time to first-to-break, rotations increasingly perform similarly to sequences whilst mixtures increasingly perform similarly to mosaics (at a longer time to first-to-break). It is difficult to determine the quantitative consequences of higher female exposure because of competing effects on the probability of resistance and time to first-to-break. At practically plausible levels of female exposure (< 0.8), the probabilities of resistance between strategies are not so dissimilar (e.g. at female exposure of ~ 0.4), where mixtures show an increase (on sequences) in the time to first-to-break of ~ 100%, mosaics up to ~ 75% and rotations of ~ 25%, which is equivalent to seven, five and one additional years of insecticide susceptibility respectively. With higher female exposure at ~ 0.6, rotations are very similar to sequences, but mixtures and mosaics are more dissimilar with a ~ 150% and ~ 100% increase in the time to first-to-break that only amounts to an additional five and three years of insecticide susceptibility respectively. Higher female exposure is expected in settings with highly anthropophilic mosquito species and contexts such as sub-Saharan Africa [33] and/or high ITN coverage such as in more affluent African nations [2]. With lower female exposure at ~ 0.1, the main strategies are hard to distinguish but all show a < 10% increase in the time to first-to-break on sequences that represents up to an additional three years of insecticide susceptibility. Lower female exposure is expected in settings with highly zoophilic mosquito species and contexts such as India [33] and/or low ITN coverage such as in regions with more isolated rural communities [34].

## Discussion

Against the backdrop of widespread resistance to existing technologies, the program to eradicate malaria is at a critical juncture as new insecticides enter their final stages of development for use via the main tool in the fight against malaria in insecticide-treated bed nets (ITNs). The near-synchronous development of these new insecticides offers the current opportunity to build resistance management into mosquito control methods and maximize the chance of eradicating malaria. Here, building directly on existing modelling [17, 24, 25], resistance-management strategies using multiple insecticides are compared to suggest how to deploy combinations of available and new insecticides on bed nets for maximum impact. Although the strategy comparison considers what to do with any new insecticide, special attention to SC1, which is the only new insecticide that is known to the authors in having a strobilurin-like chemistry that is unusual for having a target-site that is encoded in the mitochondrial genome. Massive simulations across randomly sampled sets of parameters are run to compare basic strategy concepts for two insecticides (of rotations, mosaics and mixtures under the assumption of equal initial control) against benchmarks (a ‘non-strategy’ minimum of a sequence of one insecticide at a time and a ‘best-case’ maximum where an exposed mosquito gets the full impact of both insecticides simultaneously). Given that strategies are setup for control equivalence to make the comparison ‘fairer’, (unsurprisingly) the results are only appreciably different for the measures of the time until the first and second resistance allele reach > 50% frequency (or break).

For the time until the second-to-break, each strategy nearly always has > 10% difference than a non-strategy (i.e. sequences) but < 10% difference amongst each other, which favours the use of any strategy. Yet, strategies tend to lead to much more variability in the time until the first-to-break. The non-strategy of using insecticides in sequence has < 10% difference to any strategy (rotations, mosaics or mixtures) for the time to the first-to-break when the exposure of female mosquitoes to ITNs is low (< 30%) across combinations of resistance allele inheritance (Fig. 3). Low female exposure would arise in contexts with more zoophilic mosquitoes, such as in rural India [33], and/or lower ITN coverage, such as in inaccessible regions of sub-Saharan Africa [2]. However, malaria eradication programs should aim for universal coverage to provide community-wide protection from vector control [9, 34], so most deployment scenarios can assume higher female exposure (> 30%). In this context, a strategy (rotations, mosaics or mixtures) leads to at least > 10% difference in the time until the first-to-break. Mixtures tend to have > 10% difference than the other strategies when the effectiveness of both insecticides is higher (> 35%; Fig. 3). As a new insecticide is required by WHO guidelines to have an effectiveness that is > 80% in a standard cone assay [27], using SC1 or another new insecticide alongside a partner in a mixture increases the time to first-to-break by between 50 and 100% (or + 4 and + 8 years from the estimated and relative baseline of non-strategy taking 8 years) depending on whether the partner insecticide has lower or higher effectiveness (Figs. 4, 5, 6 panel E). When one or other insecticide has higher effectiveness (> 35%), then mixtures tend to have < 10% difference than mosaics but > 10% difference than rotations or a non-strategy (Fig. 3). Consequently, using SC1 or another new insecticide alongside a partner in a mosaic increases the time to first-to-break by between 50 and 25% (or + 4 and + 2 years) depending on whether the partner insecticide has lower or higher effectiveness (Figs. 4, 5, 6 panel E). By contrast, a rotation uniformly increases the time to first-to-break by 25% (or + 2 years) irrespective of the effectiveness of the partner insecticide. Given that one insecticide has high effectiveness (> 80%), there is some variability in the quantitative difference between strategies across female exposure (Figs. 4, 5, 6 panel F), which could imply that different strategies might be suitable in geographic contexts where different levels of ITN coverage are obtainable. However, differences between strategies still supply a strong signal with high focal insecticide effectiveness (> 80%) to afford the same pattern of results across partner insecticide effectiveness, which become more pronounced with higher female exposures. Therefore, although different strategies are favoured under different parameter combinations (Fig. 3), the minimal restriction of WHO criteria on insecticide effectiveness is enough to suggest that mixtures tend to produce the greatest delay in the evolution of resistance across relevant parameter combinations.

Using the same parameter framework, the modelling presented here supports a similar result as in Levick et al. [17], but with some important differences. First, the simulations that are run here are more massive (1 million vs 10,000), which introduces less variability into the outputs. Second, the model is run in a way that explicitly categorizes data types, which extracts additional information from a run where resistance does not arise within the 500 generations of the simulation. Third, more strategies are compared with greater concern for making a ‘fair’ comparison, using the additional consideration of population size to ensure that strategies have the same average initial control, which helps to more distinctly isolate the effects of differences between strategy concepts. Consequently, when Levick et al. [17] report that mixtures tend to be favoured over sequences (with > 20% difference) in the time to first-to-break when female exposure is < 60% (and, by the range of parameter, > 10%) and effectiveness of both insecticides is > 70%, the simulations and their analysis here afford us confidence in clarifying this result. Mixtures are not implemented in the same way as in [17] in order to make all strategies that are compared here have equal initial control; although the pattern of results are similar, the quantitative differences between mixtures here and in Levick et al. [17], which is implemented in the maximum benchmark, are substantial (Fig. 4). The results presented here do not support the suggestion that mixtures tend to be favoured over sequences when female exposure is < 60%. As the analysis in Levick et al. [17] excludes comparisons where resistance does not arise within 500 generations for either mixtures or sequences, this biases the results against mixtures for higher female exposures because a mixture is more likely to delay the time until resistance to be > 500 generations (compare sequences and maximum in Figs. 1, and 4 panel C). Consequently, the restriction of mixtures being favoured to lower female exposure < 60% is likely to be an artefact of the strategy comparison in Levick et al. [17].

The results presented here do support the general finding that higher insecticide effectiveness tends to favour mixtures over other strategies, but the threshold is lower at > 35% effectiveness. Moreover, mixtures and mosaics tend to be favoured over sequences when one or other insecticide has effectiveness > 35%, which is a marked relaxation of this limit. As such, when mixtures are excluded from strategy comparison (e.g. were they not to be possible for manufacturing reasons), mosaics tend to be favoured in their stead. Therefore, the results here support the general findings about high effectiveness in Levick et al. [17] that is focused on in subsequent analysis [24, 25], especially in contrast to the forerunning ideas from interpretation of the findings in Curtis, the extensions that are implemented within the same parameter framework for two insecticides enable us to clarify that temporally-invariable strategies (mixtures/mosaics) tend to be favoured over temporally-variable strategies (rotations/sequences) in this model. In strategic terms, this suggests that the advantages of simultaneous selective pressures tend to outweigh the advantages of variable selective pressures.

What strategy does the modelling suggest should be adopted for SC1 and other new insecticides? When addressing this question, it is important to understand that this modelling exercise has excluded some factors from consideration (due to computational constraints), which could have an impact on the preference for a strategy. For example, a mixture of insecticides would need to have compatible physio-chemical properties. Accepting such limitations, the modelling and analysis here would support the use of a mixture for a variety of reasons and, on the balance of resistance-management and other considerations for SC1, especially a mixture with a pyrethroid. Firstly, there is a significant advantage of using multiple insecticides in concert. This advantage primarily comes from delaying the evolution of resistance to the first insecticide that breaks, where it can more than double the time that a population broadly remains susceptible to that insecticide. There is also an advantageous effect on the second-to-break in contrast to a non-strategy (sequences), but this effect is around a 10% delay. Secondly, the advantage of using SC1 in particular alongside another insecticide is likely to be even more acute because of its unusual property of having a target-site that is encoded in the mitochondrial genome. Were resistance to evolve as a target-site mutation, there is a ~ 70% chance that the mitochondrial resistance allele would be the first-to-break, and so using multiple insecticides in concert may be especially important for SC1. This cannot be used to suggest that having a mitochondrially-inherited target-site is undesirable for resistance management because the modelling here only examines the impact of selection in the rate of spread of a pre-existing resistance allele, and excludes the time it may take for a resistance allele to arise in the first place, which is expected to be longer than with a nuclear resistance allele because of haploidy and maternal inheritance; the time to mutation is likely to be especially important for SC1 because a survey of fungicide use in Africa shows a very low historic application of strobilurin chemistries, suggesting that there is minimal to no prior or secondary selection for resistance to this chemistry from agriculture (unpublished data, Bristow and Firth). Thirdly, despite uncertainties, using SC1 or another insecticide as part of a mixture is likely to be more robust in delaying the evolution of resistance than other strategies. With a new insecticide having to have high effectiveness (> 80%) to meet WHO criteria [9, 27], a mixture tends to outperform other strategies across insecticide effectiveness and female exposure, which represent the key dimensions of partner choice and geographic variability (Figs. 4, 5, 6). Fourthly, the time to resistance is delayed more by choosing a mixture strategy over other strategies than by choosing one insecticide partner over others. This has relevance to the choice of partner insecticide where there is pre-existing pyrethroid-resistance that reduces pyrethroid effectiveness: Although a mixture of two new insecticides is ideal from the focused-perspective of delaying the evolution of resistance, a mixture of a new insecticide with a pyrethroid (that tends to have lower effectiveness < 80%) is preferable to non-mixture strategies with two new insecticides. Fifthly, for a mixture with SC1, a pyrethroid has a major advantage in reducing the overall cost per ITN compared to a new insecticide. As a significant part of the cost of an ITN is the cost of the insecticide and pyrethroids are very cheap compared to new insecticides, the relative time of delayed resistance between partner insecticides with higher or lower effectiveness is marginal compared to the cost difference. A cheaper ITN would presumably enable more ITNs to be procured in a given region. Sixthly, a mixture strategy in the form of a single ITN is practically robust through immunity to deployment error or noncompliance, which adds ignored complexity to the assessment of rotations and mosaics.

The strategies that are being discussed are idealized; deployment error and noncompliance could make real-life rotations and mosaics more like other strategies in this model. For example, what has been described as a mosaic elsewhere occurs at such a fine-scale that it is like mixtures are here (e.g. Corbel et al. [35]), or occurs at such a coarse-scale that it is like sequences are here (e.g. Hemingway et al. [36]). In contrast, if two insecticides are put onto one net, this is robustly like the mixture strategy is here. Lastly, the criteria for WHO approval require ITNs to pass stringent safety and efficacy tests, which for a mixture ITN would require both insecticides to pass safety and efficacy standards independently and together. This adds additional economic cost and development time for mixture ITNs (which is problematic), albeit that these additional costs are lessened with the partner insecticide being a pyrethroid because it has already obtained WHO approval. Therefore, conditional upon the feasibility of its manufacture, the use of SC1 (and other new insecticides) alongside a partner insecticide in a mixture could help to build resistance-management into the bed net design, but there are significant economic challenges to producing a mixture, such that a pyrethroid may be an attractive choice for its cost-effectiveness. Further work is needed to understand how to balance resistance-management benefits and economic costs to ensure high levels of mosquito control for a sufficiently long time to provide the greatest chance of eradicating malaria.

## Conclusions

This study builds on the well-known modelling framework used in Levick et al. [17] with significant improvements on parameter space explored, the strategies considered, and also on computational resources employed. Here, mixtures tend to be a far superior conceptual resistance-management strategy across most of the parameter space. And, when it comes to deploying new insecticides for use in ITNs (e.g. SC1), their durability and impact can be maximized if these are integrated into a mixture product concept (even when the mixture partner is a pyrethroid). The theoretical results presented here can serve as a predictive guideline to bring these new insecticides to market in an evolutionarily robust way—minimizing the effects of resistance—and improving the chances of eradicating malaria in the coming decades.

## Supplementary Information

**Additional file 1. **Comparison of methods between this study and Levick et al. [17].

**Additional file 2. **Figure legends for supporting conditional inference trees.

**Additional file 3.** Supporting conditional inference trees.

## Data Availability

Data derived from the simulations executed for this study are available at the Zenodo Digital Repository: https://doi.org/10.5281/zenodo.5074771. The source code behind the model is available in GitHub as an R package called ‘detsims’: https://github.com/rkanitz/Madgwick-Kanitz-detsims. Instructions on how to reproduce the simulations and analyse the results are provided in the package’s README file, also showed as the main content in the package’s GitHub homepage.

## Abbreviations

ITN(s): Insecticide-treated bed net(s)
IVCC: Innovative Vector Control Consortium
C: Abbreviation for mosaic resistance-management strategy
f_(♀/♂, a/o)_: Genotype frequencies (for females ‘♀’ or males ‘♂’ and for adults ‘a’ or offspring ‘o’)
MM: Mitochondrial + Mitochondrial genetic inheritance
MN: Mitochondrial + Nuclear genetic inheritance
MM: Nuclear + Nuclear genetic inheritance
N_(♀/♂)_: Population size (for females ‘♀’ or males ‘♂’)
R: Abbreviation for rotation resistance-management strategy
SC1: Syngenta Compound 1
WHO: World Health Organization
X: Abbreviation for mixture resistance-management strategy
