# Supplementary material for: Modelling new insecticide-treated bed nets for malaria-vector control: how to strategically manage resistance?
Source: Malar J. 2022 Mar 24;21:102. doi: 10.1186/s12936-022-04083-z (PMC8944051; doi:10.1186/s12936-022-04083-z)
Supplement: Supplementary file 1 — Additional file 1. Comparison of methods between this study and Levick et al. [17]. [file 12936_2022_4083_MOESM1_ESM.docx]

# Additional file 1 for Madgwick & Kanitz: Modelling new insecticide-treated bed-nets for malaria-vector control: How to strategically manage resistance?

This document includes supplementary information about the comparison of methods between this study and the paper that it builds on (Levick *et al.*, 2017).

# Comparison of methods

This section does not describe the methods of this paper, but instead describes the key differences between the methods of Levick *et al.* (2017) and ours. Although this paper uses the same underlying mathematical model of fitness, we have not used the same codebase, which enables the results presented here to act as an independent verification of the results in Levick *et al.* (2017) using a similar methodology. One of the major differences arises in the choice of parameter space, where Levick *et al.* (2017) pick the upper and lower limits of parameter ranges for comparison to Curtis (1985), without discussing the choice of range in detail, and randomly sample within the parameter range for a simulation with 10,000 runs. Levick *et al.* (2017) restrict the parameter range to exclude resistance cost and its dominance term but include a term relating to the correct deployment of mixtures that is ignored here, which results in a parameter space with 11 parameters. Instead, here, the full range of each parameter is explored across different scenarios using a random sample of 1,000,000 parameter combinations. We have also made a series of additions, alterations and exclusions to the model and approach in Levick *et al.* (2017) as a result of three key changes that are relevant to the specific questions of this paper, detailed below.

## Mitochondrial inheritance

The simulations in Levick *et al.* (2017) focus on resistance alleles with nuclear inheritance, albeit that X-chromosomal inheritance is briefly considered but not for analysis using simulations. With special consideration of SC1, there is a need to incorporate an insecticide where target-site resistance evolves at a mitochondrial locus. A resistance allele at a mitochondrial locus is different from a nuclear locus (including the X-chromosome) because of maternal inheritance, (effective) haploidy and the irrelevance of dominance terms. Although SC1 is known to have a target-site that has mitochondrial inheritance, this does not mean that resistance will only ever evolve at the mitochondrial locus as resistance could evolve through mutations that affect regulatory factors or metabolic pathways that would most likely have nuclear inheritance. Further, other new insecticides may not also have a mitochondrially-inherited target-site. As a result, simulations to assess the evolution of resistance to new insecticides are run for combinations of resistance alleles with different modes of inheritance: nuclear inheritance only (nuclear and nuclear; often abbreviated to NN to describe each locus), mixed inheritance (or mitochondrial and nuclear; MN) and mitochondrial inheritance only (or mitochondrial and mitochondrial, MM).

Mitochondrial inheritance also impacts the treatment of linkage disequilibrium because it is transmitted between the generations in (what is essentially) the manner of asexual reproduction. In Levick *et al.* (2017), the differential selection of females and males is permitted to build differential linkage disequilibrium in each sex. Here, this is extended to describe the mitochondrial mode of inheritance (and also make the nuclear mode of inheritance comparable). This adds a substantial degree of complexity to the model because selection must be recorded and accounted in terms of sex-specific haplotypes. Consequently, we also simplify the parameter framework in Levick *et al.* (2017) to ignore the ‘low concentration insecticide niches’ (that can be used to capture the decay of insecticides on bed-nets over their deployment lifespan), which means that here a mosquito is either exposed to a dose of insecticide(s) or it is not.

Similarly, in Levick *et al.* (2017), resistance cost is only applicable to mosquitoes that are not exposed to insecticide, which simplifies the computational process by reducing the dimensions of the parameter space. However, this assumes that resistance cost has a complicated epistatic effect on fitness that is dependent upon the exposure parameters – and hence why resistance cost is dropped in their simulations. Instead, here, resistance cost is attributed irrespective of whether or not a mosquito is exposed to insecticide, which is arguably more biologically realistic. Further, this resistance cost is attributed irrespective of sex (*i.e.* also to males), which makes the model more complicated but again arguably better reflects biological reality.

## Population control

The metric used in Levick *et al.* (2017) to measure strategy success is the time it takes for a resistance allele to reach 50% frequency within the population. With two insecticides, this leads to two resulting measures: the time for the first resistance allele to reach 50% frequency (first-to-break) and the time for the second resistance allele to reach 50% frequency (second-to-break). This approach is consistent with the majority of genetic models in the literature (REX Consortium, 2010), but is only indirectly related to the goals of resistance management in mosquito control and malaria eradication. For example, as dominance is varied, there is a relevant distinction between resistance allele and phenotype frequencies, as a recessive resistance allele may spread to appreciable frequencies with only a minimal impact on mosquito control. Moreover, deceptively, focusing solely on a genetic assessment of strategy success may inappropriately imply that the optimal resistance-management strategy to delay the time it takes for a resistance allele to reach 50% frequency would be not to deploy the insecticide at all, but this would not be a very effective method of mosquito control. In reality, the reduction in the population size of mosquitoes is itself only an indirect measure of the ultimate aim to reduce malaria transmission, but there is no obvious conflict between mosquito control and reducing malaria transmission in the way that there is between slowing the spread of resistance and mosquito control. Yet, it is worth considering that only female mosquitoes transmit malaria because male mosquitoes do not blood-feed, and so control should reflect the special interest in the female population size. Therefore, a balance needs to be struck between slowing the spread of resistance alleles and the extent of female mosquito control, so both qualities are measured here.

The inclusion of mosquito population size into the parameter framework in Levick *et al.* (2017) requires the incorporation of a model of mosquito demography. There is a significant challenge in formulating an accurate demographic model that is compatible with the genetic model in Levick *et al.* (2017). Here, a simple logistic model is used, which can be used to compare strategies but is not calibrated to give an accurate picture of mosquito population change – which is, indeed, also true of the genetic model in Levick *et al.* (2017). Additionally, an eco-evolutionary approach is adopted, which integrates the change in population size and allele frequencies that would be unchanged in the absence of insecticide. Whilst using the same basic mathematical model, population size is integrated into the model with minimal change. Parents have their population size and allele frequencies modified in relative terms by selection upon interaction with insecticides. Parents may survive between the generations at a rate equal to the complement of the intrinsic death rate. Offspring arise from the random mating of parents, with population size affected by the intrinsic birth rate and density-dependent mortality (that is known to principally affect larvae; Charlwood, 2020). As such, consistent with the simulation framework in Levick *et al.* (2017), the evolutionary process is modelled in the order of mortality selection on adults from exposure to the insecticide, random mating among surviving adults and offspring production to form a new generation of adults. There is some evidence that most African mosquito species primarily mate before females start their blood-feeding cycle (Takken *et al.*, 2006), which would mean that females acquire sperm before exposure to the insecticide on bed-nets, which could potentially change the strength of selection because the surviving females would have mated before any selection from the insecticide on males (assuming non-zero male exposure). Females mating before blood-feeding is equivalent to zero male exposure, but females mating after blood-feeding is also captured by the framework in Levick *et al.* (2017) when there is non-zero male exposure. So, herein, it is important to note that the modelling setup with variable male exposure makes the assumption that males are subjected to selection before mating (and this may not apply to all mosquito species). Therefore, overall, the model of mosquito demography is basic with population size following a standard logistic model whilst allele frequencies follow standard models of population genetics.

The logistic model is used to give an equivalent threshold metric for mosquito control, equivalent to how the time it takes for a resistance allele to reach 50% frequency is a metric for the spread of resistance. Across the scenarios considered, it is important that the chosen threshold is low enough that the female mosquito populations start below it in the first generation that the insecticide is applied, high enough to provide a large quantitative separation between strategies and not-too-high that most female mosquito populations do not reach the threshold within the timeframe that the model is examined across (500 generations). In-keeping with previous work (Gould, 1986), we chose to use the threshold of the time that it takes for the female population to recover to 80% of its size prior to application of insecticides to balance these considerations. Yet, with the incorporation of demography, there is the possibility of population extinction were the population size to drop below one, which affords a new data type for analysis. It would also be possible to calculate a metric for initial control, but this is used to setup a fairer strategy comparison and so does provide any additional information for the analysis here (see next section).

## Strategy comparison

In Levick *et al.* (2017), strategy comparison of mixtures and sequences uses the time until resistance in the first- and second-to-break measures. Practically, two insecticides may well be the maximum number of insecticides that can be developed as mixtures on bed-nets due to the requirement of compatible physiochemical properties, but other strategies could easily have more insecticides. However, to make a like-for-like comparison, it seems appropriate to restrict attention to two insecticides. In Levick *et al.* (2017), mixtures involve the use of two insecticides until they both fail (*i.e.* 50% resistance allele frequency); there is some consideration of the incorrect deployment of mixtures (though not with using simulations), which is not examined here. Sequences involve using one insecticide until it fails before switching to the other until it fails. Necessarily, the setup of strategies introduces a bias in favour of mixtures because a mixture gains the advantage of deployment alongside a partially-effective insecticide (that has >50% resistance allele frequency) whereas a sequence never involves the deployment of an insecticide that is partially-effective. Accordingly, the first-to-break is a more comparable measure of strategy success, which is given much more attention in Levick *et al.* (2017) and also here. A challenge of using this measure, which is not addressed in Levick *et al.* (2017), is that some of the strategies under comparison (like sequences) involve using insecticides in a particular order, whereas others do not (like mixtures). To avoid introducing a bias and to ensure that a like-for-like comparison is most often made, the insecticide that is first-to-break when solo goes first in the order, which makes the same insecticide most likely to be compared across strategies. This approach becomes particularly relevant when two modes of inheritance are in question (*i.e.* MN).

The biggest difference between the strategy comparison in Levick *et al*. (2017) and here is in the strategies themselves. Here we examine five resistance-management strategies, albeit that two are treated as ‘benchmarks’ for the comparison of the other three. As in Levick *et al.* (2017), sequences provide the default strategy (or, really, non-strategy) of solo deployment where each insecticide is used until it is no longer effective. The insecticide that is the first-to-break is deployed first for comparability (as described in the previous section). Sequences provide a ‘lower benchmark’ for comparing other strategies in establishing a ‘no resistance management’ baseline, like a negative control. At the other end of the spectrum, we use the same formulation of the mixture strategy as in Levick *et al.* (2017) to be an ‘upper benchmark’ like a positive control. Although dependent upon the dose optimisation process, this strategy is potentially unrealistic because it supposes that the insecticides have the same effectiveness (in terms of mortality) irrespective of whether they are in a mixture or solo. As such, the formulation of the mixture strategy as in Levick *et al.* (2017) is treated here as the ‘maximum’ strategy that sets the upper benchmark, which enables the other strategies to be compared within limits of possibility.

The three main strategy concepts under comparison are rotations, mosaics and (our sense of) mixtures. For rotations, insecticides are switched on a fixed term cycle, which corresponds to the three-year replacement or retreatment schedule of bed-nets of 36 generations. The insecticide that is the first-to-break is deployed first for comparability (as described in the previous section). For mosaics, a mosquito has an exclusive 50% chance of being exposed to one or other insecticide, which is as if ITNs are equally distributed throughout a population. The relationship between rotations and mosaics can be summarised by initial control, which is calculated as the complement of mean fitness ($1-\bar{\omega}$; see next section for fitness equations). The average initial control from rotations when both insecticides are first deployed is equal to the initial control from mosaics, which sets up as close a like-for-like comparison as possible with rotations only differing from mosaics in the temporal dimension of insecticide deployment. In deviation from their attribution in Levick *et al.* (2017), mixtures can also be attributed to have the same average initial control as rotations and mosaics to make for a similarly like-for-like comparison. Mixtures differ from mosaics in that if a mosquito is exposed to the ITN, it is inclusively exposed to both insecticides. This can be standardised to ensure the same average initial control by applying a constant to the effectiveness of each insecticide, which reflects the dose optimisation process, which can be thought of as implying rough cost-equivalence (given that the cost of the ITN is largely the cost of the insecticide). Such considerations of economic cost may seem superfluous for a comparison of strategy concepts as in here, but they are essential for making fair comparisons among practical alternatives.

An additional practical constraint of the simulations in Levick *et al.* (2017) is that the measure for strategy comparison is only recoded if it is within a finite timeframe because of computational constraints. In Levick *et al.* (2017), the relevant timeframe for a resistance-management strategy was identified as 500 mosquito generations; this roughly corresponds to 40 years (assuming a generation per month), which exceeds the time it would take to develop a new insecticide for use on ITNs. Consequently, the analysis in Levick *et al.* (2017) excludes all comparisons where at least one strategy leads to a resistance allele not reaching 50% frequency within 500 generations. Necessarily, this approach excludes comparisons where one strategy is highly successful at delaying the evolution of resistance, which in the comparison in Levick *et al.* (2017) would bias results against (their sense) mixtures (or, our sense, the maximum benchmark). The extent of the bias is difficult to assess, but it would potentially start to matter much more when a larger number of strategies are compared because it is more likely for at least one to take more than 500 generations to reach 50% resistance allele frequency. Therefore, so as not to introduce a bias against comparisons where a particular strategy is very successful, if a strategy does not reach 50% resistance allele frequency within 500 generations but the resistance allele nonetheless has an increasing frequency, then the output can be given a nominal value above the upper limit to include this outcome in as far as possible in the strategy comparison.

## Simulation analysis

The primary method of analysis of simulation data uses classification trees, which is similar to how simulation results are presented using in Levick *et al.* (2017). A major difference between Levick *et al.* (2017) and here is that Levick *et al.* (2017) only need to compare two strategies (mixtures and sequences), and so they used a simplistic approach to classification tree analysis (Barbosa & Hastings, 2012). Here, we need to compare multiple strategies at once, whereupon conditional inference trees provide a robust analysis for the categorical classification of when a particular strategy tends to be favoured in different regions of the parameter space for a particular measure (*e.g.* first-to-break). Trees are built and drawn using R:ctree (in the ‘partykit’ package) (Hothorn *et al.*, 2015), which uses iterative permutation tests in an algorithm that tests the independence between the inputs and output variables and makes a binary split in the variable with the strongest differentiation of output distributions. The iterations that form the tree have a controlled stop when the algorithm can no longer make a split into terminal nodes with >5% of the data, which is a control applied for the visualisation of the tree to ensure a manageable number of terminal nodes. Necessarily, there is an element of bias introduced by the choice of any parameter space, but the use of conditional inference trees mitigates against wholescale bias because the meaningful outputs do not rely upon the frequency of a category across the chosen parameter space but rather within statistically different parameter subspaces that are identified algorithmically. Therefore, whilst the frequency of categories must be interpreted with caution (as they depend on the chosen parameter space), there is only the potential for bias to enter into the output in determining the precise boundaries between parameter subspaces where different strategies are more successful. In this way, the use of conditional inference trees is aligned with the aim of understanding how some strategies are favoured over others for a particular measure, which can then provide the basis for insight into why some strategies are favoured over others in different contexts.

The classification process to assemble the output in Levick *et al.* (2017) first filtered the data to exclude all comparisons where at least one strategy takes longer than the 500 generations of the simulation, and then was either based on a ‘hard’ cut-off of whichever strategy had the longest time to resistance or biasedly requiring >20% longer time to resistance for mixtures (else sequences). Here, to make additional comparisons, non-measured data types that take longer than the 500 generations of the simulation (see Figure 1) are given nominal values that ensure their hierarchical interpretation: where ‘Toward Threshold’ is set to 1000, ‘Away from Threshold’ is set to 1500 and ‘Extinction’ is set to 2000. These simulation-outcome types, or simply “data types”, are referred to in detail in the results section. With additional strategies, the classification can include ‘sequences’ as a lower benchmark but must exclude the ‘maximum’ upper benchmark because this would mask meaningful comparisons, so comparisons are made between sequences, rotations, mosaics and mixtures. Taking one measure at a time (first-to-break, second-to-break or control-failure), the output variable is assembled by classifying which one or combination of strategies have >10% difference (in either direction) for that measure. A combination of strategies is categorised when strategies have <10% difference with each other and all have >10% difference with all other strategies. This ‘soft’ cut-off can help avoid misinterpreting the quantitative variation in the measure where strategies have near-equal results. However, this does generate a problem of intransitivity (*e.g.* where A is near-equal B, B is near-equal C and A is not near-equal C), which is best avoided by making fewer comparisons. Consequently, although results can be summarised in a tree that compares all strategies and their combinations at once (as is done in the main-text), special attention is given to a different set of trees where each strategy is examined in isolation and classified as the most successful (>10% difference than all others), the equally-most successful with one or more other strategy (>10% difference than the worst), the equally-most successful alongside all other strategies (all <10% difference) or not among the most successful (other(s) have >10% difference; Supplementary Figures S1-12).

It is particularly relevant to the development of new insecticides that there are significant challenges to the design of an ITN with a mixture of insecticides. There are a large number of research and development hurdles to the practical development of an ITN with a mixture, including that insecticides may have different physiochemical properties that are difficult to simultaneously accommodate and/or insecticides may interact antagonistically and so reduce each other’s effectiveness. Additionally, there are also criteria to meet, including that the WHO requires that both insecticides are effective on their own, *e.g.* each with >80% mortality in cone assays (where a mosquito is placed in a confined space with the ITN for 3 minutes and mortality is measured 24 hours after this exposure) and also together (WHO, 2013). Lastly, there are also economic hurdles to the success of an ITN with a mixture, both in the funding of research and development (given the other hurdles) and in the manufacturing the end-product. In case mixtures are not possible, the classification of which strategy is favoured is also rerun excluding mixtures (*i.e.* for sequences, rotations and mosaics) to consider how this alters the results.

Whilst conditional inference trees describe how different strategies are favoured in particular parameter spaces, here there is a special focus in the resistance-management strategy for SC1 and other new insecticides. As such, the secondary method of analysis concerns the results when a new insecticide is used alongside a partner insecticide and in different geographic settings. Due to the uncertainty around the genetic mechanism of resistance for SC1 or any other new insecticide, resistance could involve mutations that affect the target-site or other mutations that bring about changes in metabolism or behaviour, which has different implications for mitochondrial or nuclear inheritance, the extent of resistance restoration (and its dominance) and the resistance cost (and its dominance). Consequently, the only parameter that SC1 or another new insecticide constrains is effectiveness because of the design constraint from WHO criteria, requiring that a new insecticide has >0.8 effectiveness to obtain prequalification listing (WHO, 2013, 2020). A new partner insecticide would be similarly constrained, but the WHO also recommends the use of pyrethroids despite widespread resistance (WHO, 2012). South and Hastings (2018) estimate the fitness model parameters for pyrethroids using data from an experimental hut study, but this estimation was restricted to *kdr* (*i.e.* target-site) resistance, whereas the genetics of resistance to pyrethroids is known to be highly heterogeneous across sub-Saharan Africa (Hancock *et al.*, 2020) and pyrethroids have been shown to have variable effectiveness on different mosquito strains from different locations – even in the same regions of sub-Saharan Africa (Strode *et al.*, 2014; Kleinschmidt *et al.*, 2018). Further, although it is typical for pyrethroids to have lower effectiveness than new insecticides because of pre-existing resistance, this does not imply anything about the genetics of new forms of resistance going forward. Therefore, to assess the restricted parameter space for resistance management with at least one new insecticide, the impact of effectiveness can be assessed by exploring the trend in time to resistance across a focal insecticide’s effectiveness and, given a focal insecticide has >0.8 effectiveness, across a partner insecticide’s effectiveness.

Assessing geographic variation also comes with similar problems for assessing the genetics of resistance, but there are qualitative differences between regions on the basis of female exposure. South and Hastings (2018) do not give an estimate of exposure because this cannot easily be quantified by any current data as it is the net result of numerous interacting factors. For example, the major malaria vectors are known to be more zoophilic (animal-feeding) outside of Africa, whilst Africa itself has co-occurring species of which the key transmitters of malaria are more anthropophilic, but it is difficult to quantify zoophily *in situ* where zoophily is both an evolved factor of ecology and a consequence of host availability in a local area (*e.g.* see comparisons in Waite *et al.*, 2017). The use of ITNs adds further complication to estimating the effect of zoophily on exposure because ITNs are likely to both select for zoophily within species and cause an ecological shift toward more zoophilic species (Stone & Gross, 2018). Setting zoophily aside, the most quantifiable aspect of exposure is ITN coverage. Although the distribution of ITNs has aimed for universal coverage to maximize mosquito control (Killeen, 2020), this aim has proved especially difficult to achieve in Africa where malaria burdens are highest. On average across at-risk populations in sub-Saharan Africa in 2018, whilst ~60% of households have access to an ITN, only 40% of households have enough ITNs for all occupants (WHO, 2019). There is also substantial variation in ITN coverage to at-risk households across sub-Saharan Africa from >80% having access to an ITN in their household in Uganda to <10% in Gabon. Therefore, although quantitative estimation of female exposure is complicated by numerous underlying factors, to assess the restricted parameter space for resistance management where a focal insecticide has >0.8 effectiveness, the qualitative impact of factors like zoophily and coverage leading to high or low female exposure can be assessed by exploring the trend in time to resistance across female exposure.
