# Supplementary material for: Modelling new insecticide-treated bed nets for malaria-vector control: how to strategically manage resistance?
Source: Malar J. 2022 Mar 24;21:102. doi: 10.1186/s12936-022-04083-z (PMC8944051; doi:10.1186/s12936-022-04083-z)
Supplement: Supplementary file 2 — Additional file 2. Figure legends for supporting conditional inference trees. [file 12936_2022_4083_MOESM2_ESM.docx]

# Supplementary Figure Legend

*The legend for any supplementary figure describes a conditional decision tree, and so has the general structure that follows a set pattern where some elements vary (as* ***highlighted as emboldened*** *and described on the subsequent pages):*

Figure S**#**. Conditional inference trees for the **W** measure for the **X** mode of inheritance. The simulation data on the first-to-break for each strategy is classified into a categorical variable to describe how focal strategy **Y** differs from its comparison set **Z**. Non-measured data types (see Figure 1) are given nominal values that ensure their hierarchical interpretation: where ‘Toward Threshold’ is set to 1000, ‘Away from Threshold’ is set to 1500 and ‘Extinction’ is set to 2000. The conditional inference tree is used to partition the data classification output based on the parameter space inputs based on the 1 million randomly sampled parameter combinations for the 17 parameters (see Table 4). Trees are built and drawn using R:ctree, which uses permutation tests to iterate an algorithm that tests the independence between the inputs and output variables and makes a binary split in the variable with the strongest differentiation of output distributions. The parameter of each split is given in the nodes within the tree, which reports the parameter (as per Table 4) and the *p*-value of the independence test; the quantitative place of the split in the parameter itself is recorded in the line between nodes. The iterations that form the tree stop when algorithm can no longer make a split into terminal nodes with >5% of the data, which is a control applied for the visualisation of the tree to ensure a manageable number of terminal nodes. The distributions of data classification are given in the terminal nodes as a bar chart, where the y-axis describes the proportion of data points.

**#** = a number between 1 and 78

**W** = first-to-break, second-to-break or control-failure; or the time it takes for the first resistance allele to reach >50% frequency, the time it takes for the second resistance allele to reach >50% frequency, the time it takes for the population size to recover to >80% of its size prior to the application of insecticides

**X** = nuclear-only (NN), mixed nuclear and mitochondrial (NM) or mitochondrial-only (MM); the mode of inheritance for each of the two resistance loci

**Y** = all strategies (ALL, like Figure 3) mosaic (C), rotation (R), sequence/non-strategy (S) or mixture (X; if included); the focal strategy in the comparison for ALL is like Figure 3, where the output variable is classified as the most successful in isolation (>10% difference than all others), jointly-most successful alongside other identified strategies (<10% difference with each other but all have >10% than the other strategies) or equally-most successful with all other strategies (<10% difference with all other strategies); the focal strategy in the comparison for any other single strategy is classified with each strategy examined in isolation and classified as the most successful (denoted with the strategy abbreviation C/R/S/X; >10% difference than all others), the jointly-most successful with one or more other strategy (J; >10% difference than the worst), the equally-most successful alongside all other strategies (=; all <10% difference) or not among the most successful (O; others have >10% difference)

**Z** = including mixtures (CRSX) or excluding mixtures (CRS); where the focal strategy is compared against all strategies with or without mixtures (X)

| Figure number (#=) | Measure (W=) | Inheritance (X=) | Focal strategy (Y=) | Comparison set (Z=) |
| --- | --- | --- | --- | --- |
| 01 | first-to-break | NN | C | CRSX |
| 02 | first-to-break | NN | R | CRSX |
| 03 | first-to-break | NN | S | CRSX |
| 04 | first-to-break | NN | X | CRSX |
| 05 | first-to-break | MN | C | CRSX |
| 06 | first-to-break | MN | R | CRSX |
| 07 | first-to-break | MN | S | CRSX |
| 08 | first-to-break | MN | X | CRSX |
| 09 | first-to-break | MM | C | CRSX |
| 10 | first-to-break | MM | R | CRSX |
| 11 | first-to-break | MM | S | CRSX |
| 12 | first-to-break | MM | X | CRSX |
| 13 | second-to-break | NN | ALL | CRSX |
| 14 | second-to-break | NN | C | CRSX |
| 15 | second-to-break | NN | R | CRSX |
| 16 | second-to-break | NN | S | CRSX |
| 17 | second-to-break | NN | X | CRSX |
| 18 | second-to-break | MN | ALL | CRSX |
| 19 | second-to-break | MN | C | CRSX |
| 20 | second-to-break | MN | R | CRSX |
| 21 | second-to-break | MN | S | CRSX |
| 22 | second-to-break | MN | X | CRSX |
| 23 | second-to-break | MM | ALL | CRSX |
| 24 | second-to-break | MM | C | CRSX |
| 25 | second-to-break | MM | R | CRSX |
| 26 | second-to-break | MM | S | CRSX |
| 27 | second-to-break | MM | X | CRSX |
| 28 | control-failure | NN | ALL | CRSX |
| 29 | control-failure | NN | C | CRSX |
| 30 | control-failure | NN | R | CRSX |
| 31 | control-failure | NN | S | CRSX |
| 32 | control-failure | NN | X | CRSX |
| 33 | control-failure | MN | ALL | CRSX |
| 34 | control-failure | MN | C | CRSX |
| 35 | control-failure | MN | R | CRSX |
| 36 | control-failure | MN | S | CRSX |
| 37 | control-failure | MN | X | CRSX |
| 38 | control-failure | MM | ALL | CRSX |
| 39 | control-failure | MM | C | CRSX |
| 40 | control-failure | MM | R | CRSX |
| 41 | control-failure | MM | S | CRSX |
| 42 | control-failure | MM | X | CRSX |
| 43 | first-to-break | NN | ALL | CRS |
| 44 | first-to-break | NN | C | CRS |
| 45 | first-to-break | NN | R | CRS |
| 46 | first-to-break | NN | S | CRS |
| 47 | first-to-break | MN | ALL | CRS |
| 48 | first-to-break | MN | C | CRS |
| 49 | first-to-break | MN | R | CRS |
| 50 | first-to-break | MN | S | CRS |
| 51 | first-to-break | MM | ALL | CRS |
| 52 | first-to-break | MM | C | CRS |
| 53 | first-to-break | MM | R | CRS |
| 54 | first-to-break | MM | S | CRS |
| 55 | second-to-break | NN | ALL | CRS |
| 56 | second-to-break | NN | C | CRS |
| 57 | second-to-break | NN | R | CRS |
| 58 | second-to-break | NN | S | CRS |
| 59 | second-to-break | MN | ALL | CRS |
| 60 | second-to-break | MN | C | CRS |
| 61 | second-to-break | MN | R | CRS |
| 62 | second-to-break | MN | S | CRS |
| 63 | second-to-break | MM | ALL | CRS |
| 64 | second-to-break | MM | C | CRS |
| 65 | second-to-break | MM | R | CRS |
| 66 | second-to-break | MM | S | CRS |
| 67 | control-failure | NN | ALL | CRS |
| 68 | control-failure | NN | C | CRS |
| 69 | control-failure | NN | R | CRS |
| 70 | control-failure | NN | S | CRS |
| 71 | control-failure | MN | ALL | CRS |
| 72 | control-failure | MN | C | CRS |
| 73 | control-failure | MN | R | CRS |
| 74 | control-failure | MN | S | CRS |
| 75 | control-failure | MM | ALL | CRS |
| 76 | control-failure | MM | C | CRS |
| 77 | control-failure | MM | R | CRS |
| 78 | control-failure | MM | S | CRS |
