# Supplementary figures and images for: Modelling new insecticide-treated bed nets for malaria-vector control: how to strategically manage resistance?
Source: Malar J. 2022 Mar 24;21:102. doi: 10.1186/s12936-022-04083-z (PMC8944051; doi:10.1186/s12936-022-04083-z)

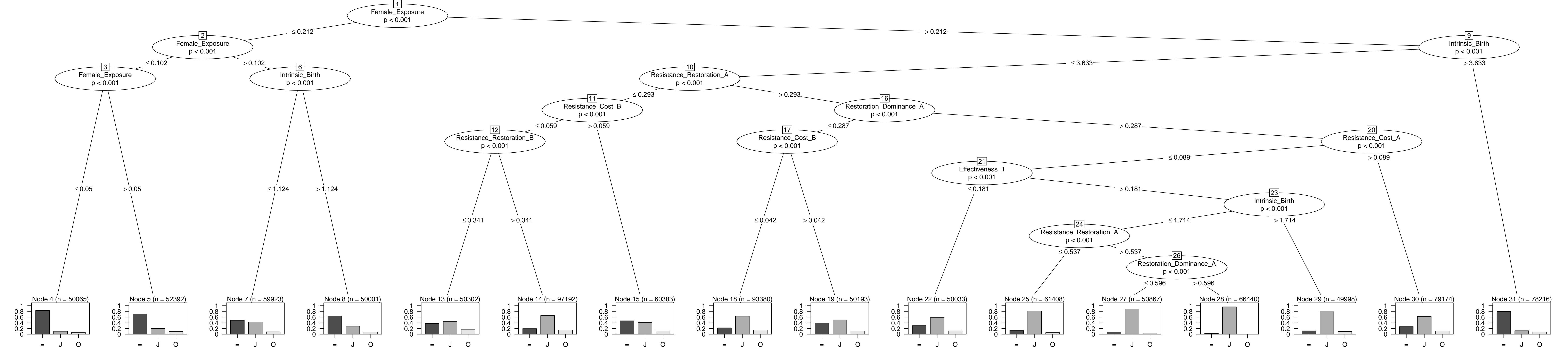

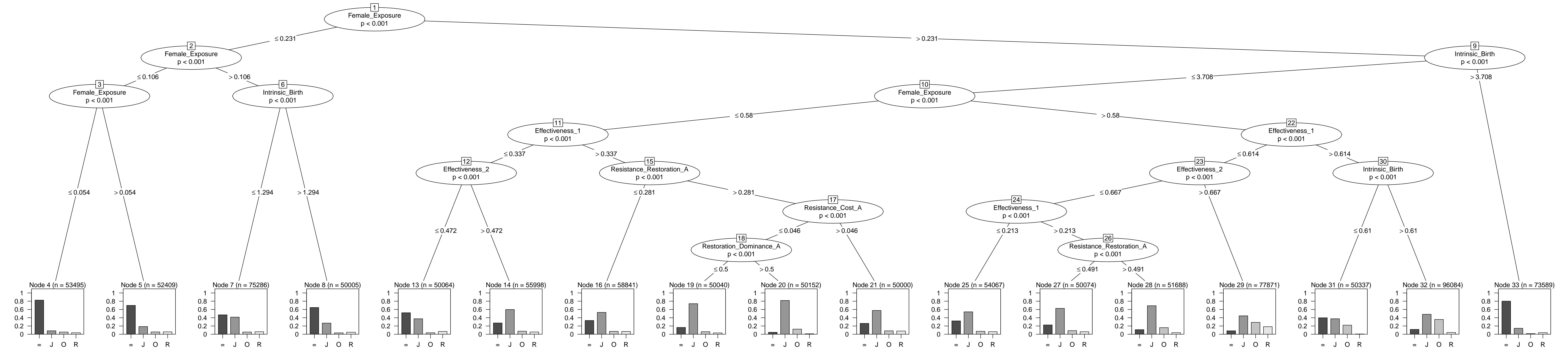

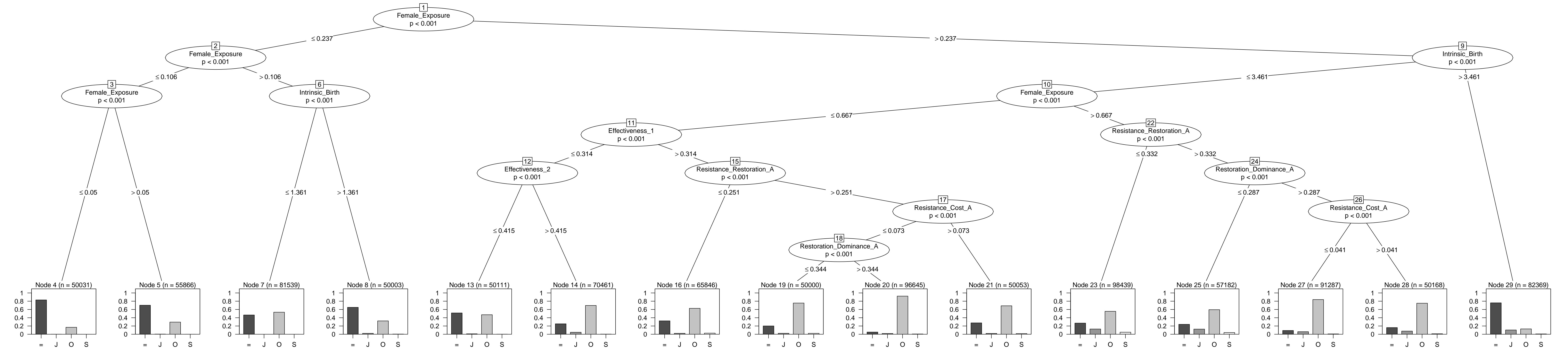

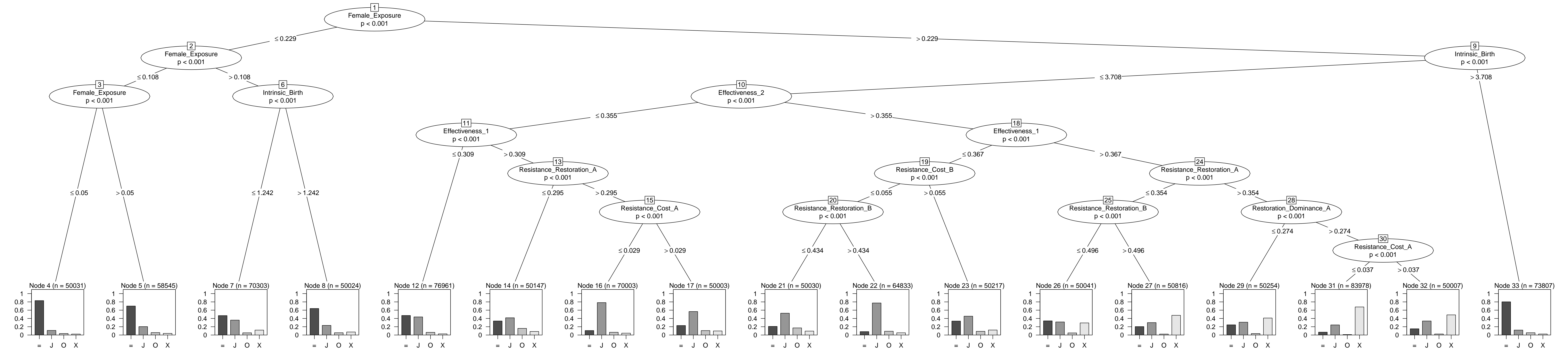

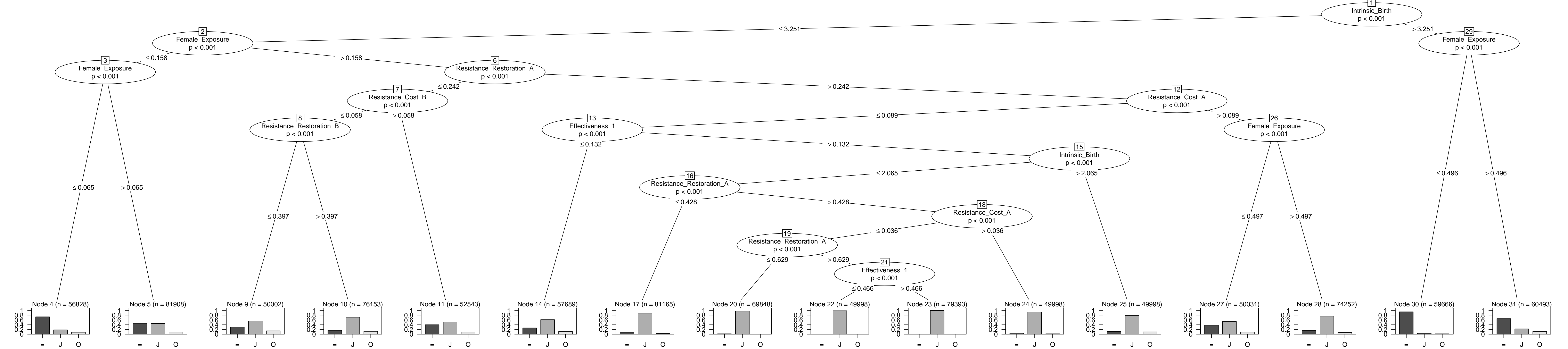

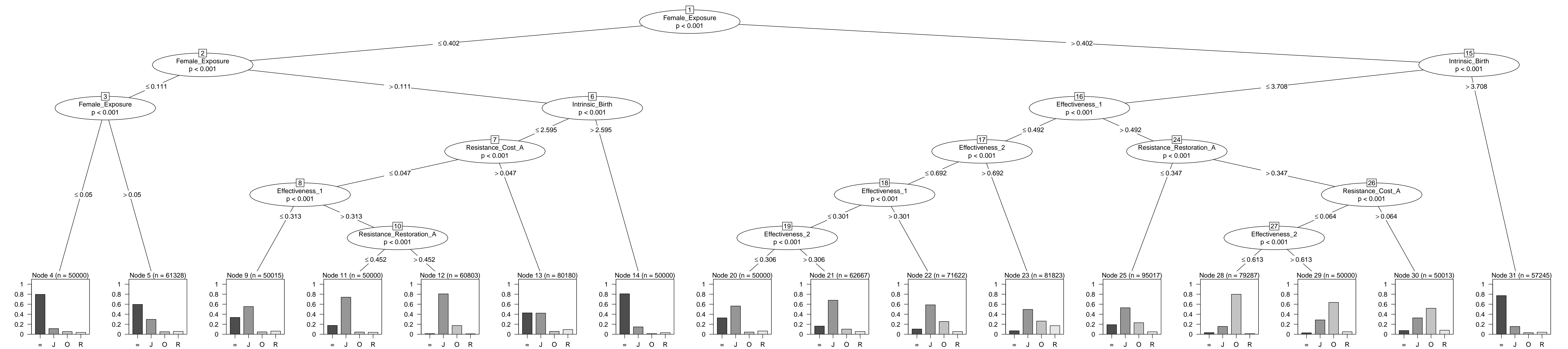

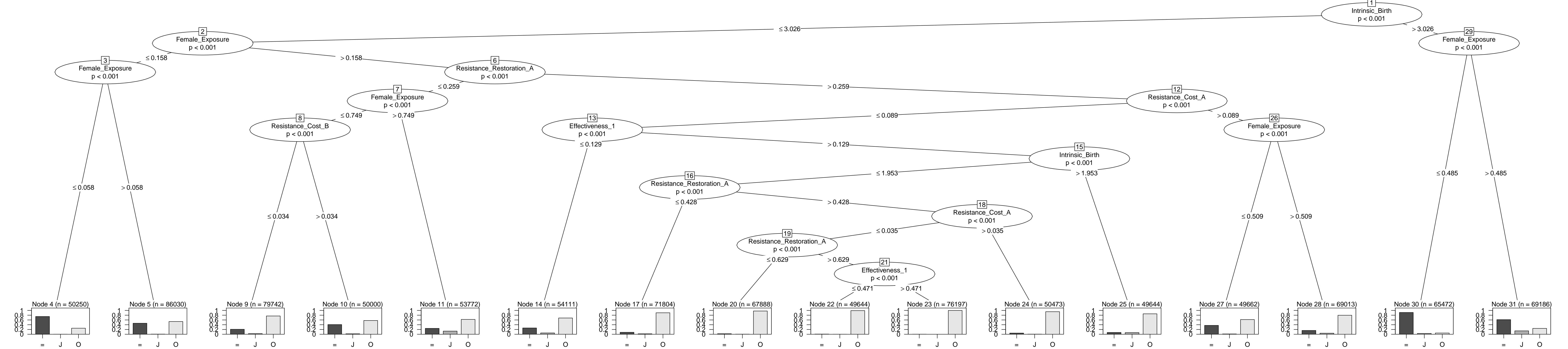

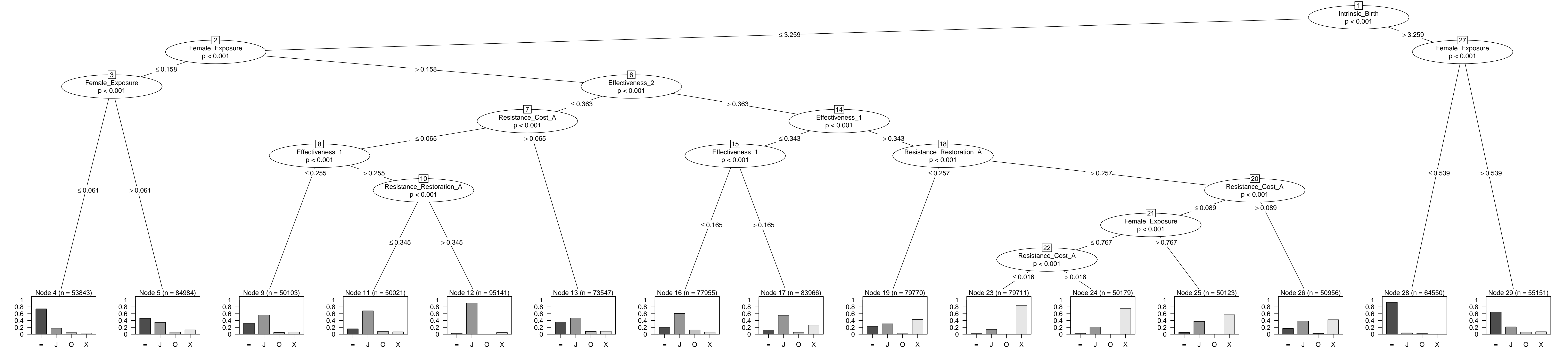

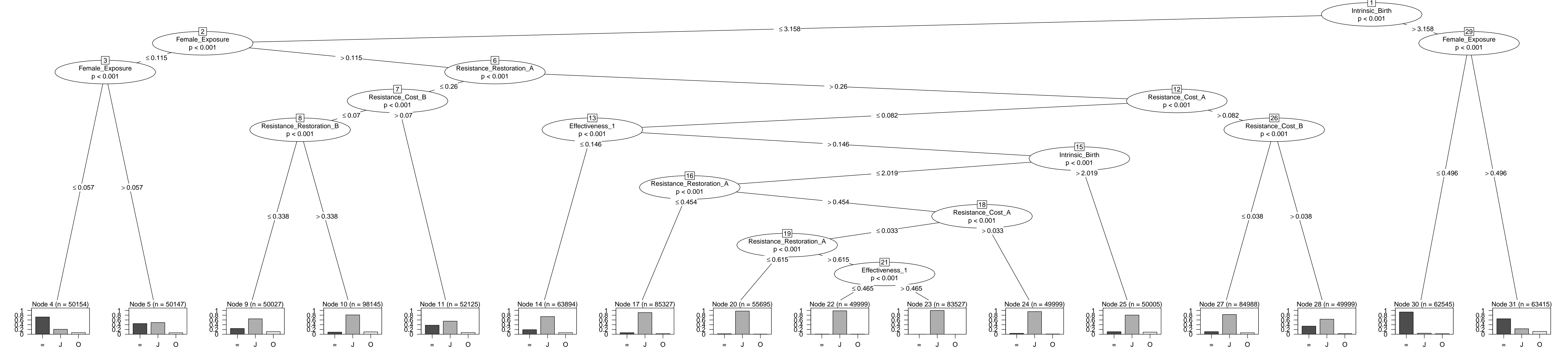

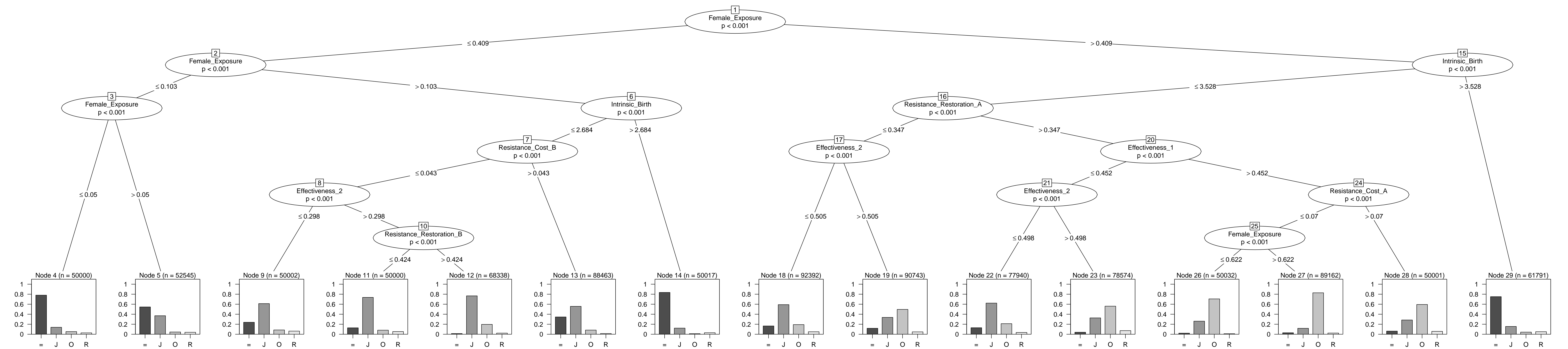

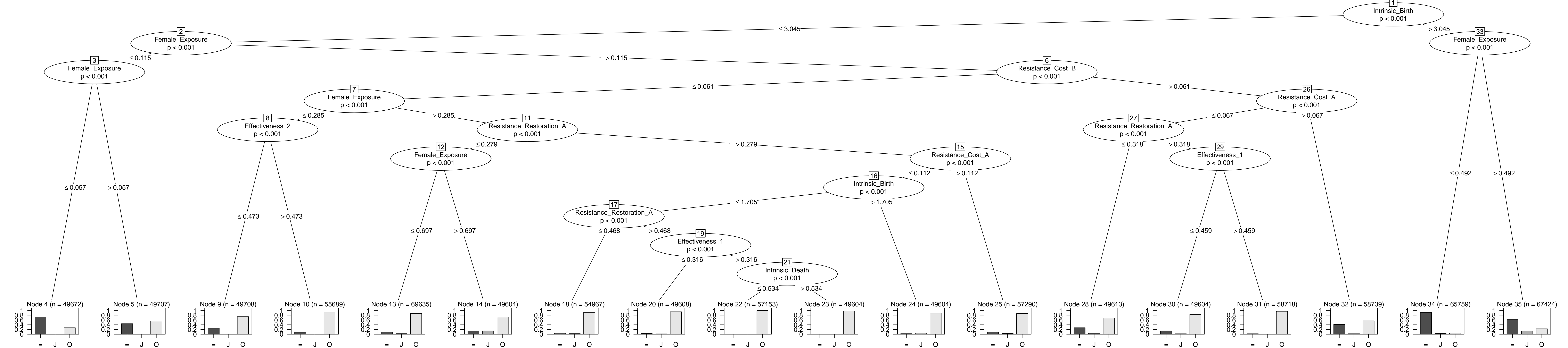

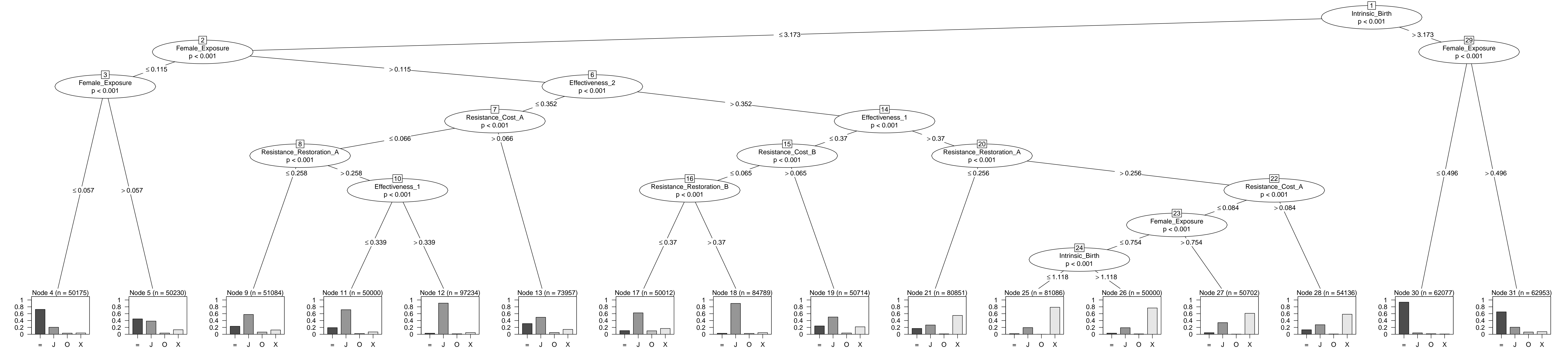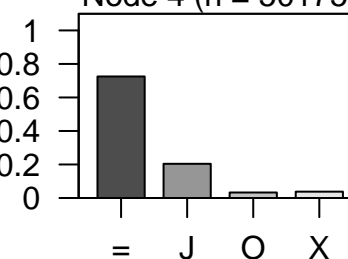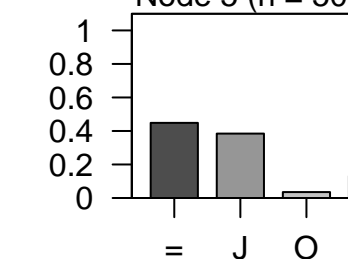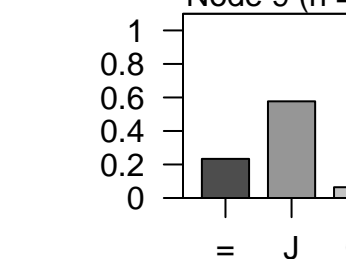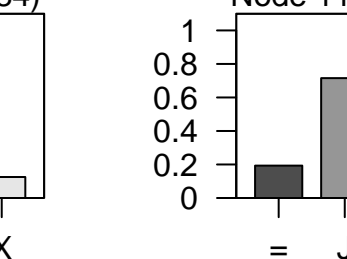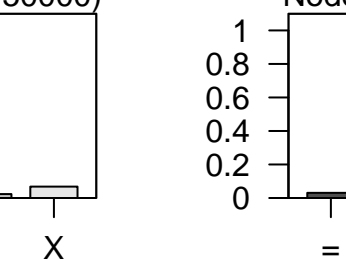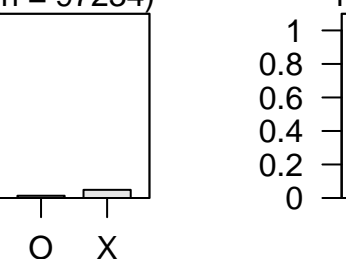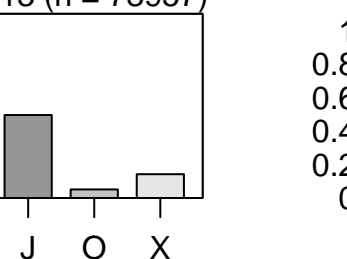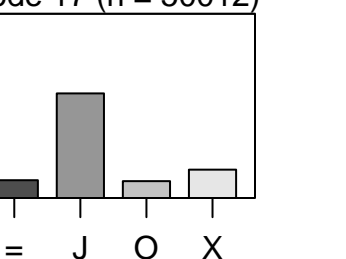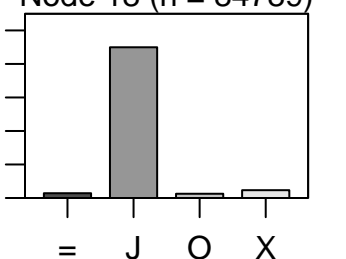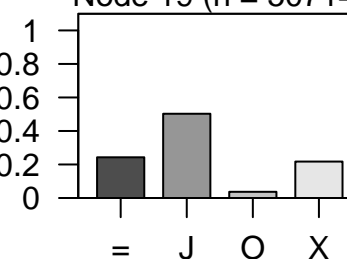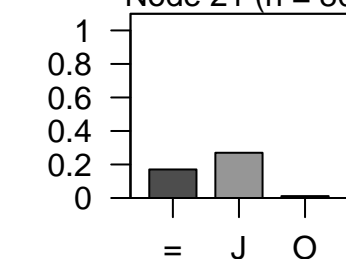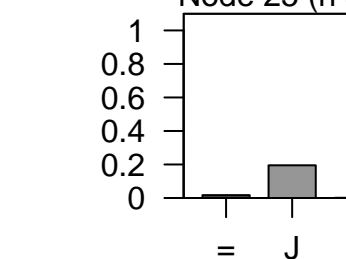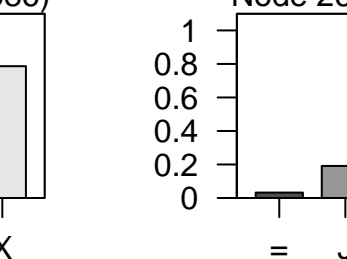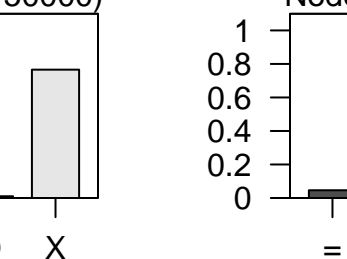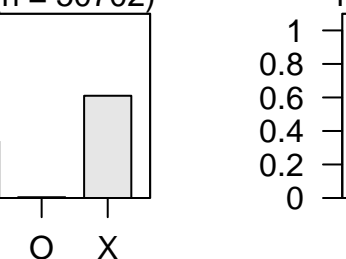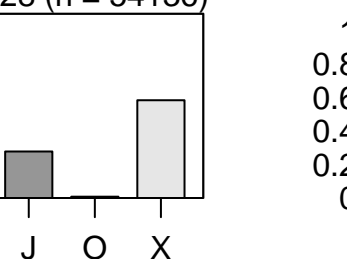

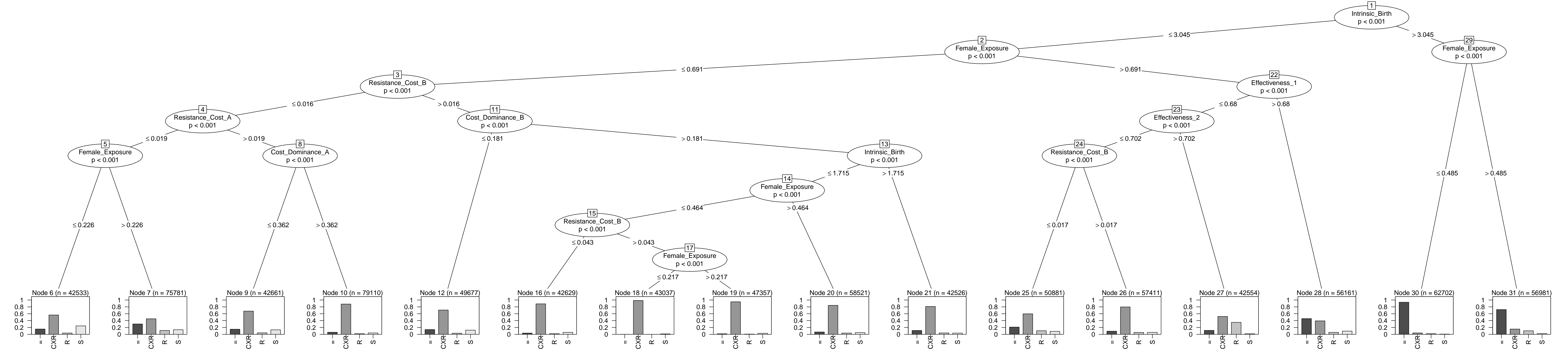

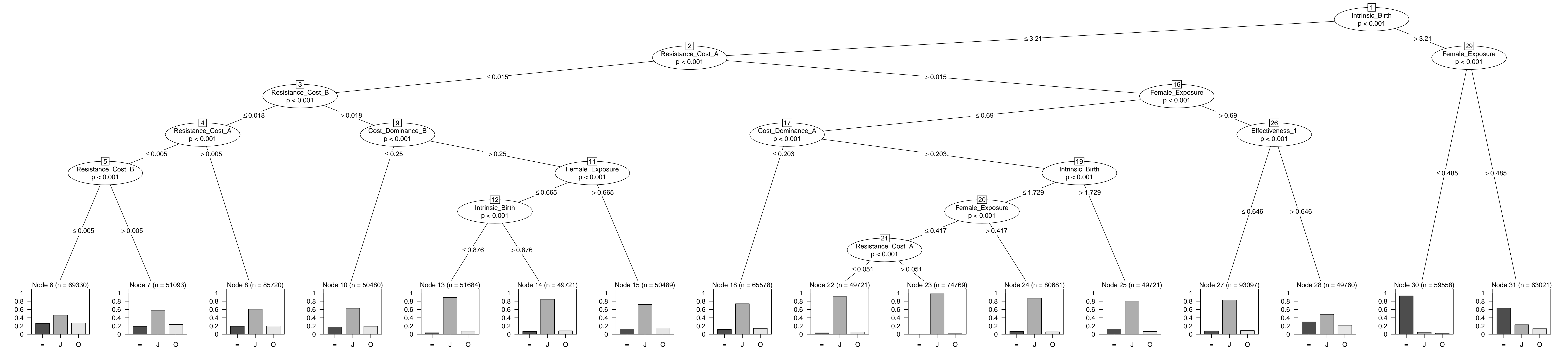

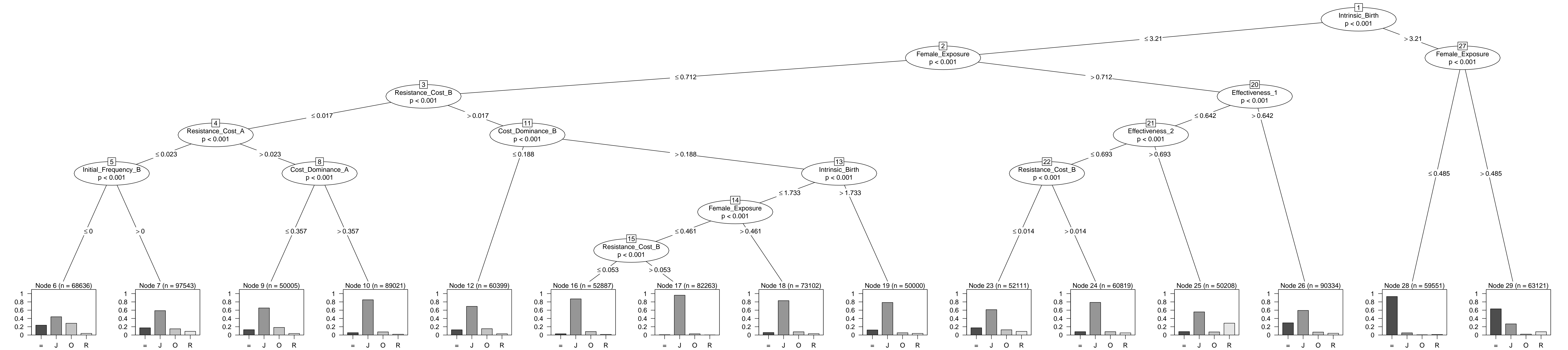

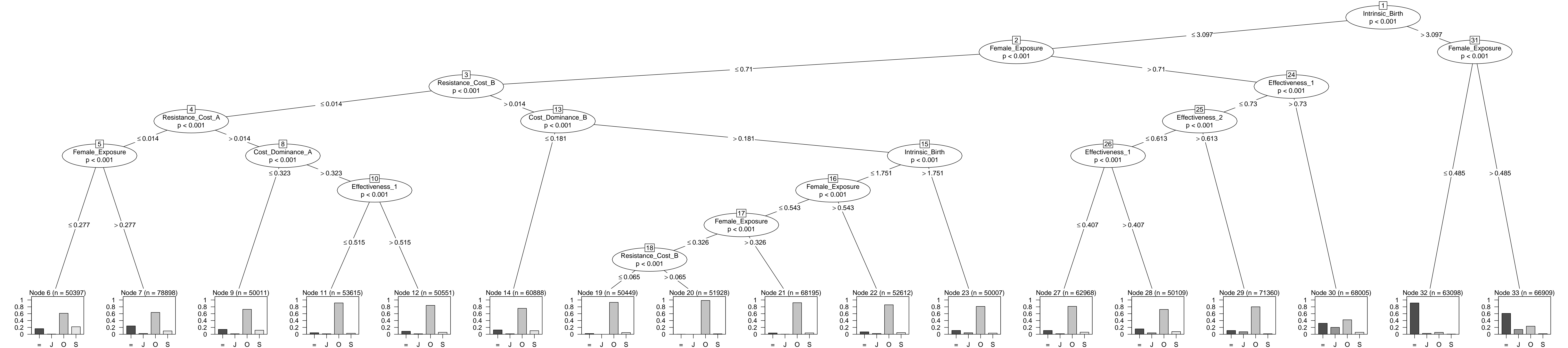

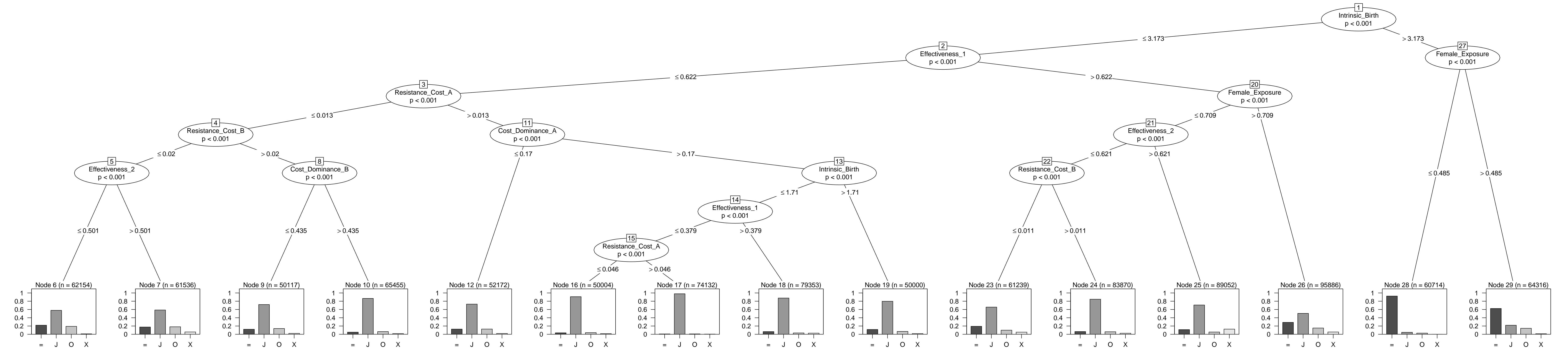

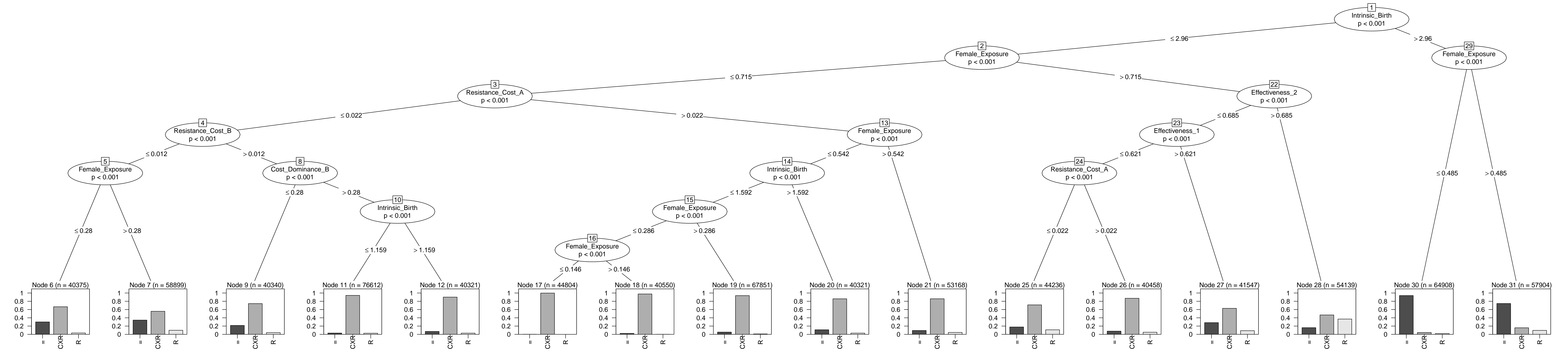

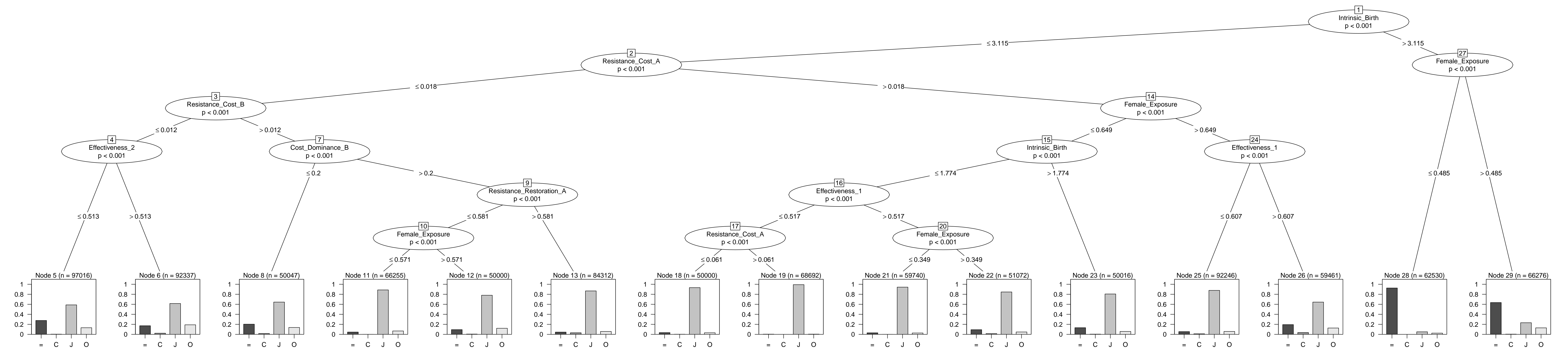

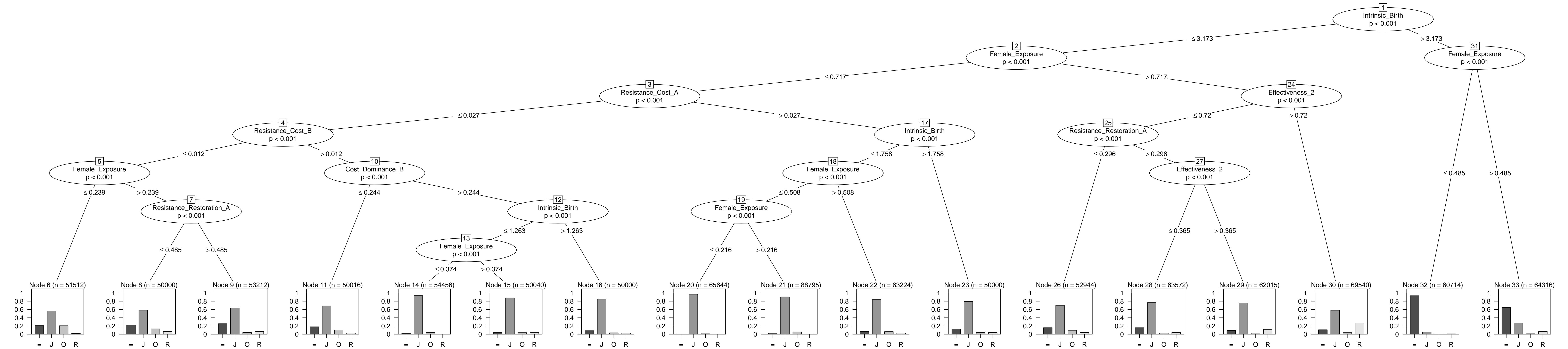

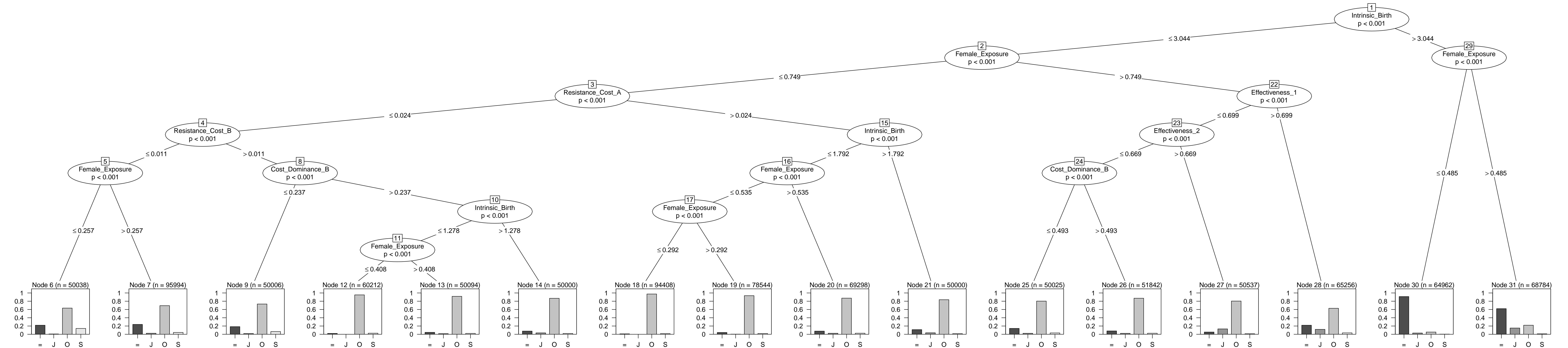

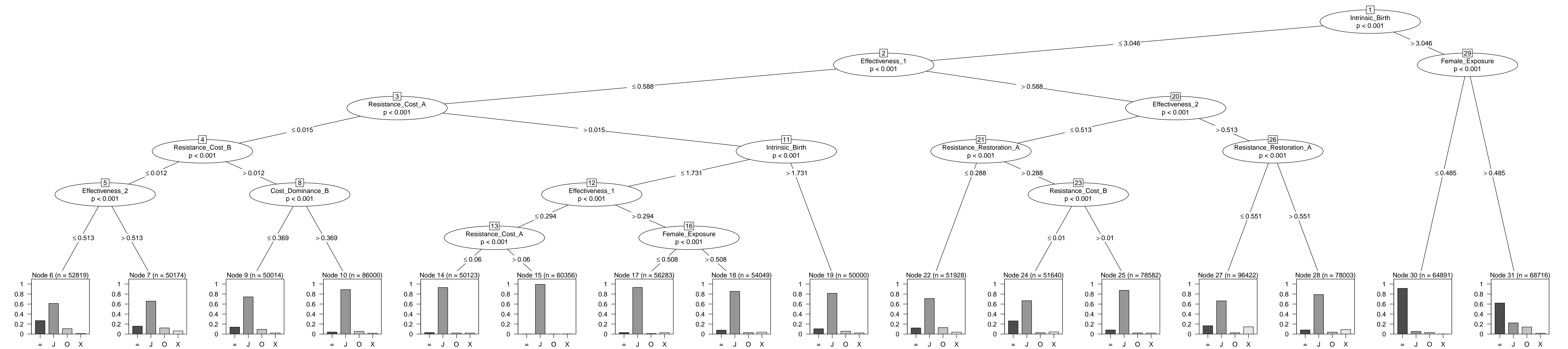

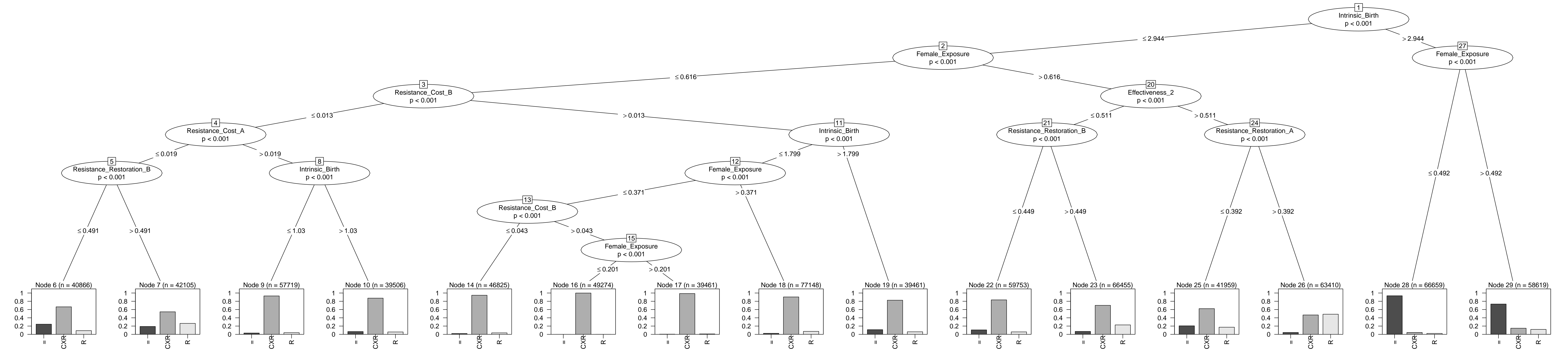

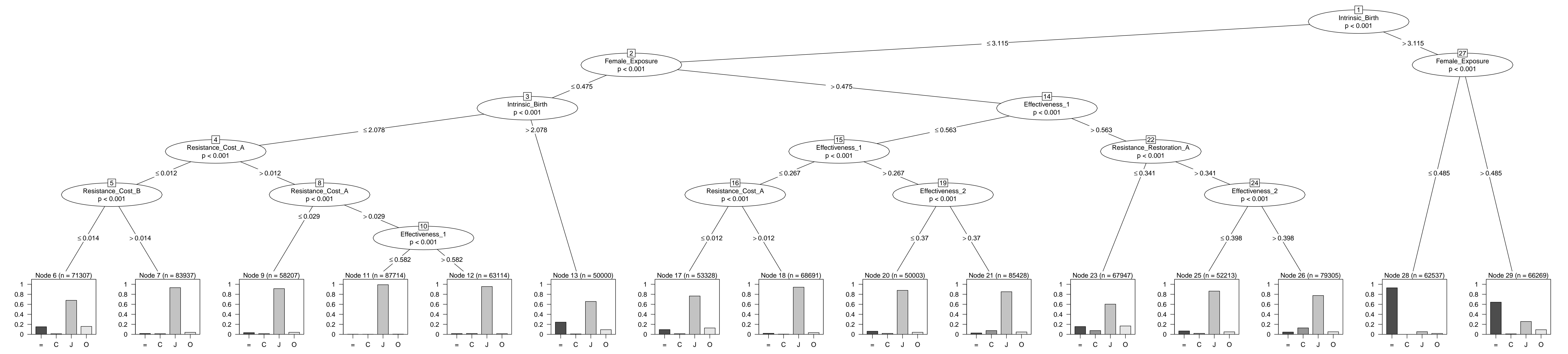

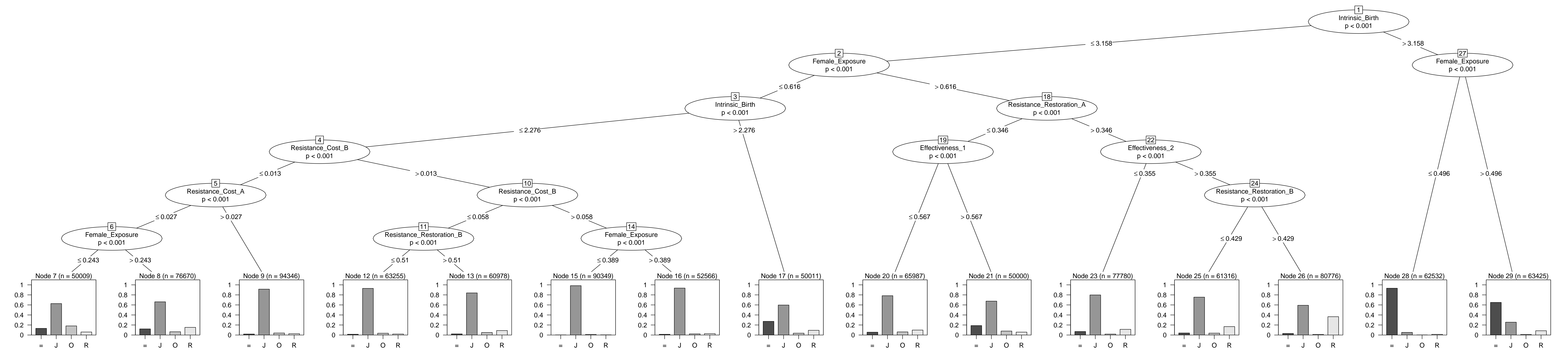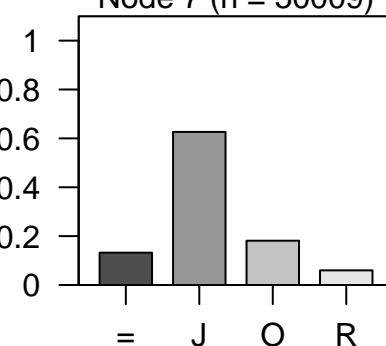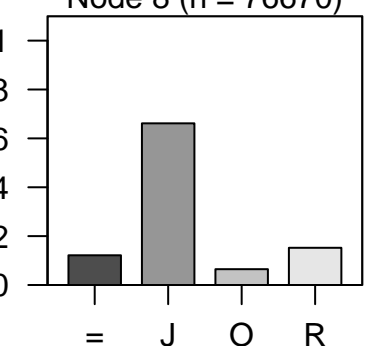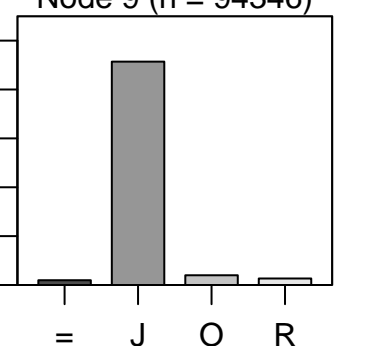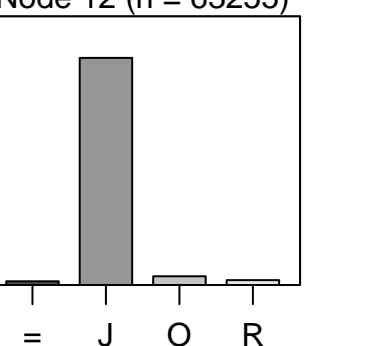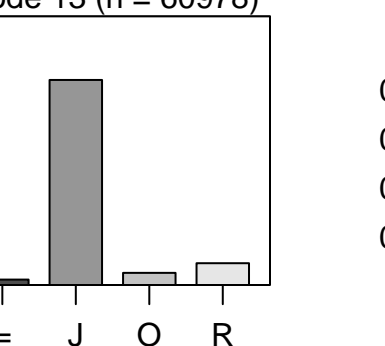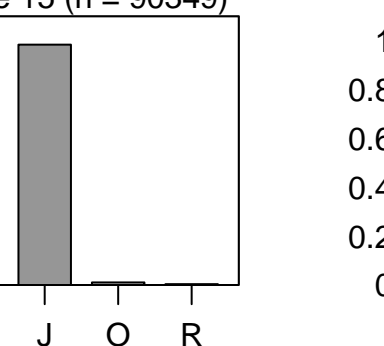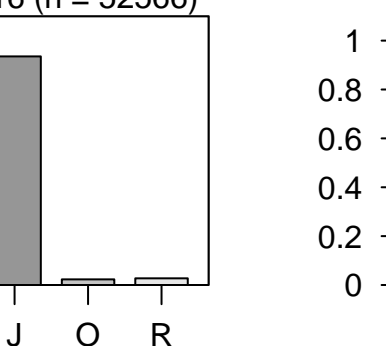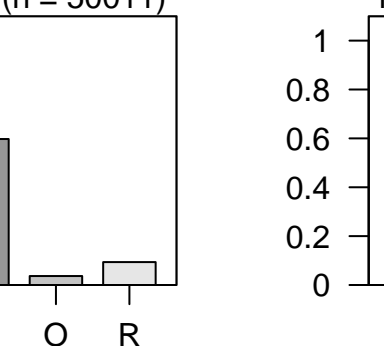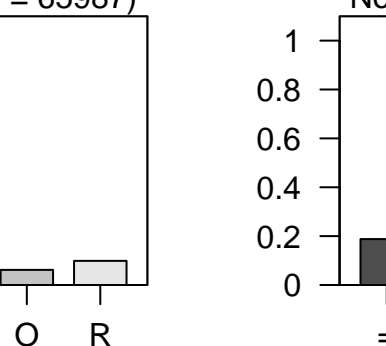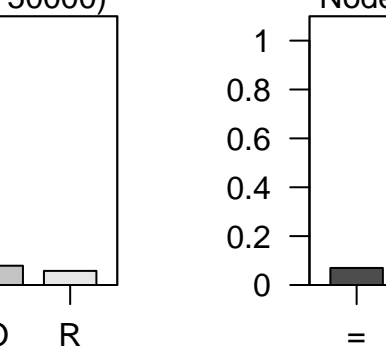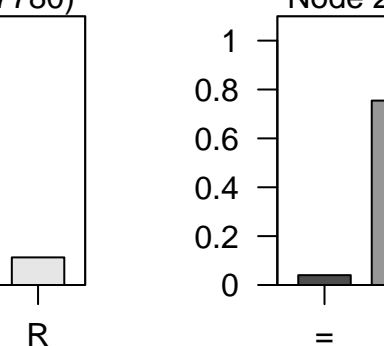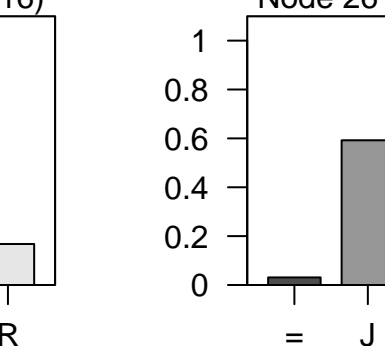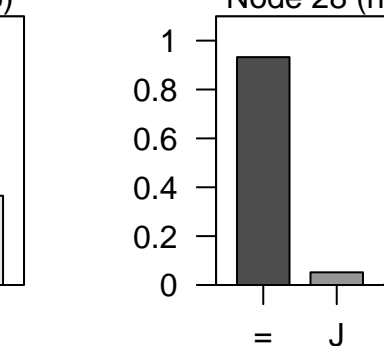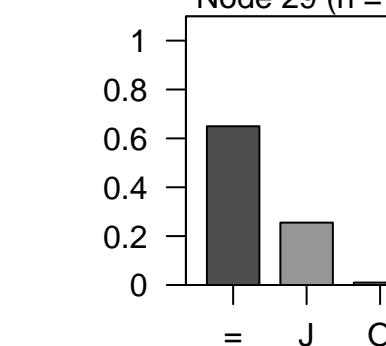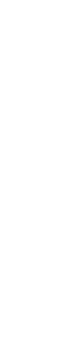

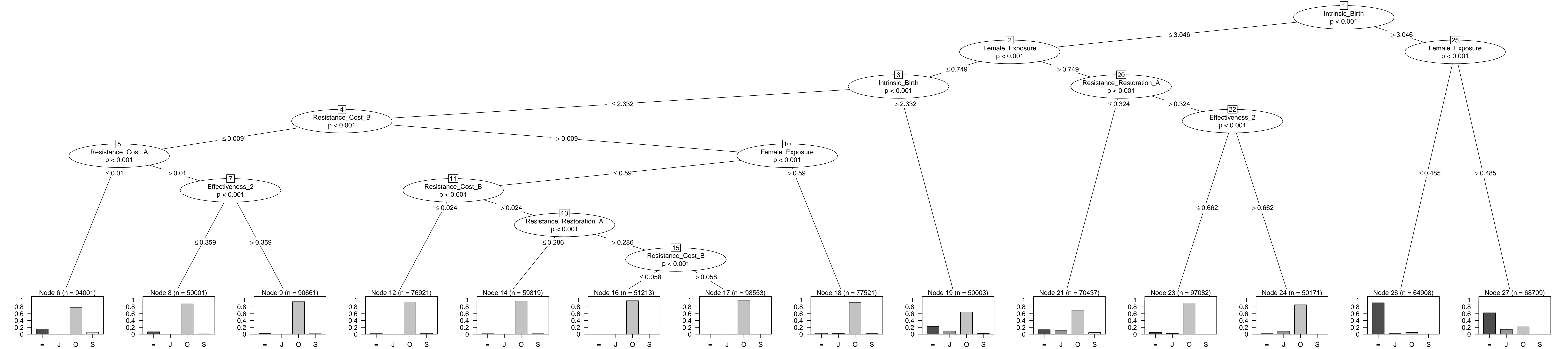

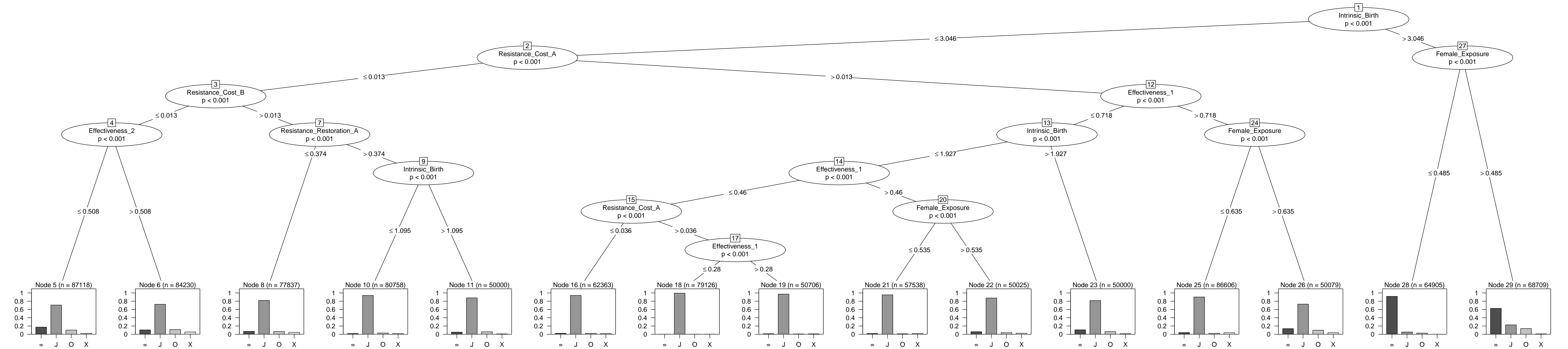

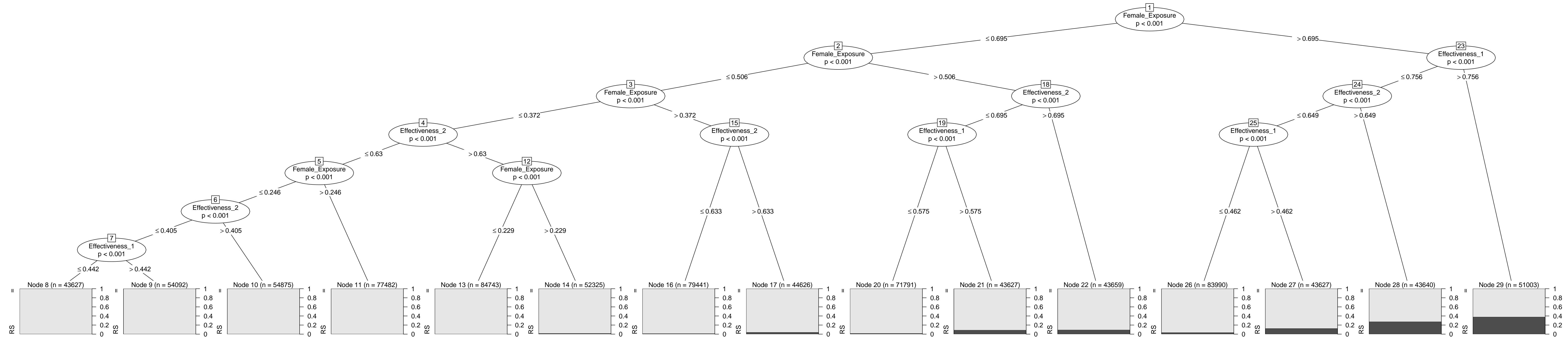

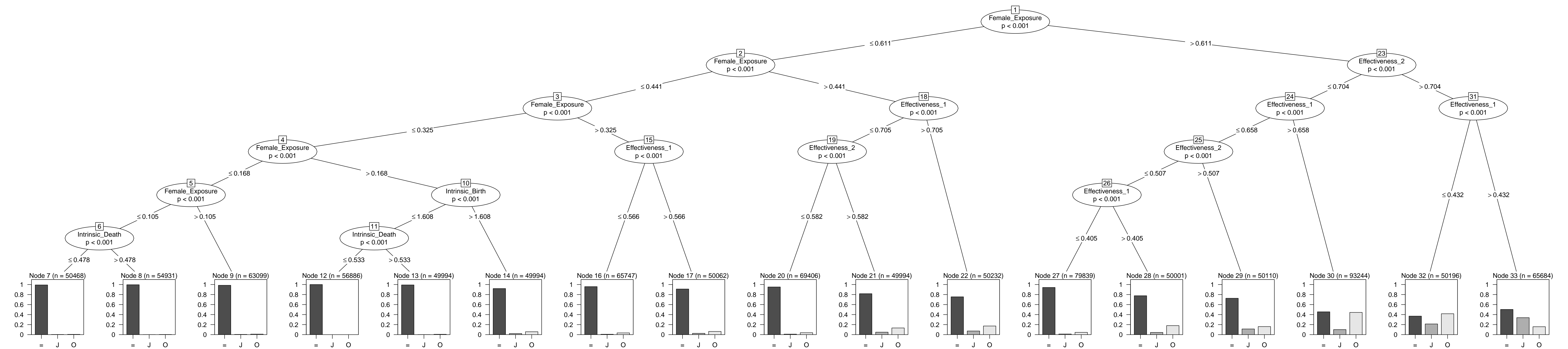

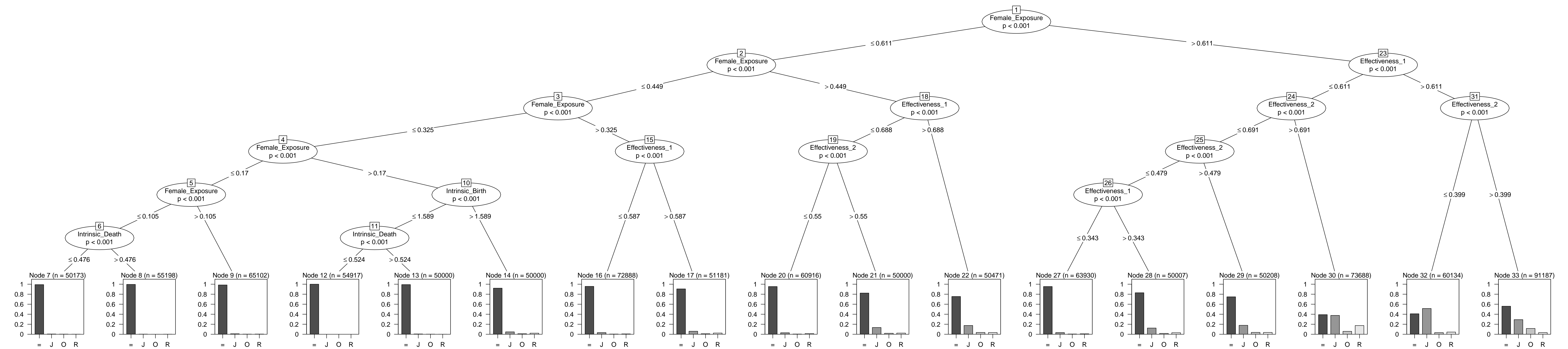

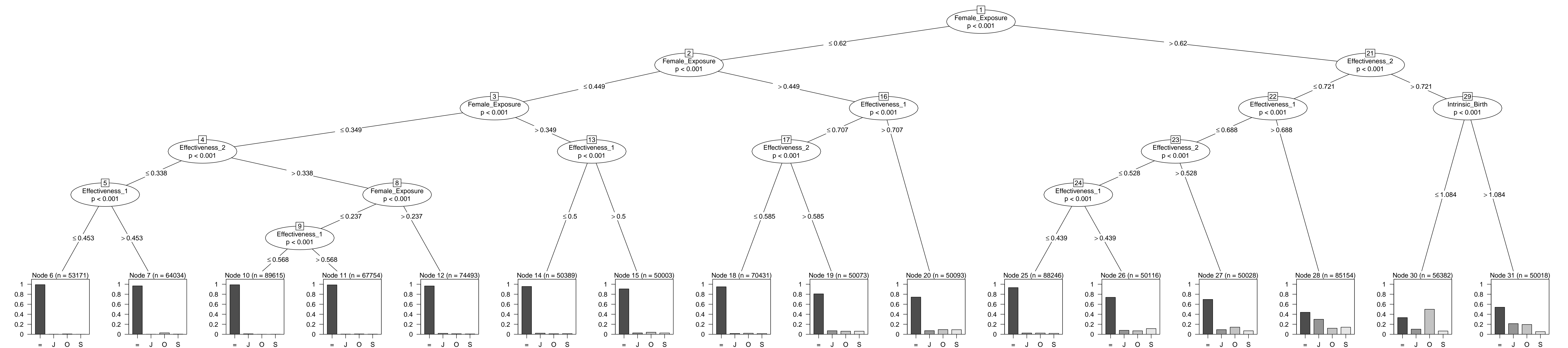

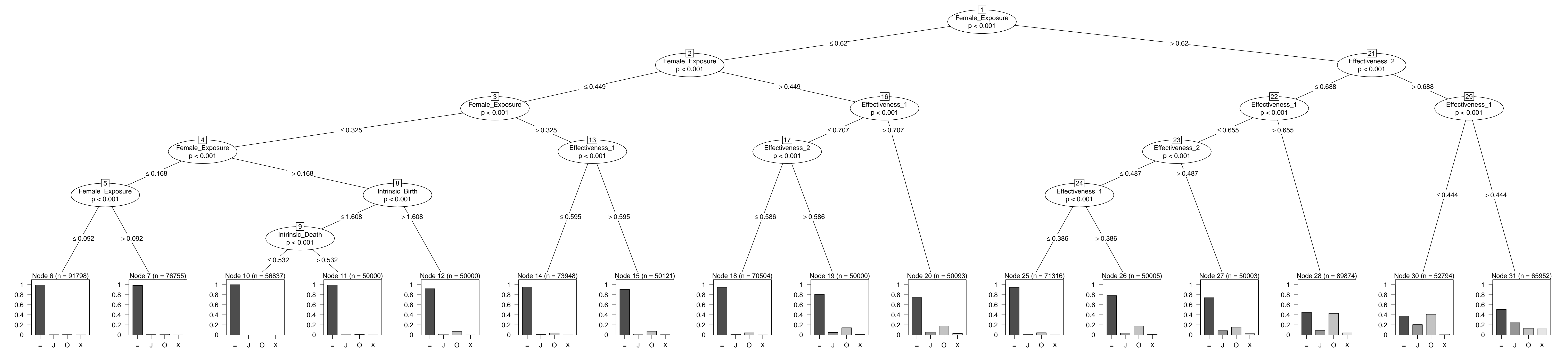

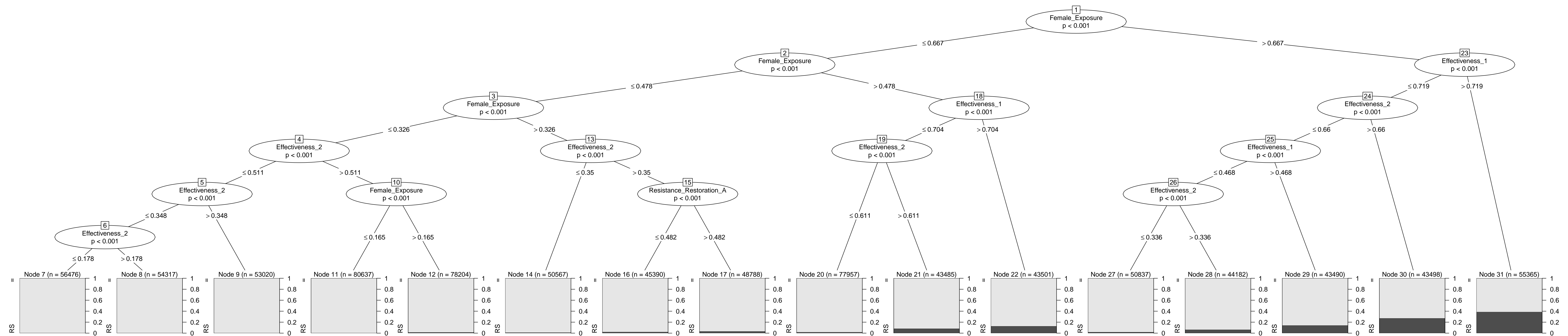

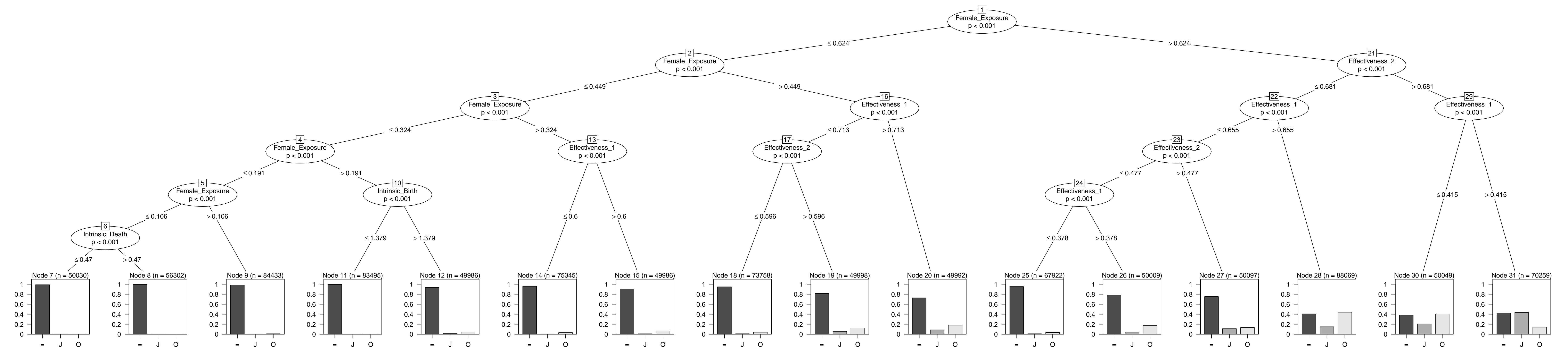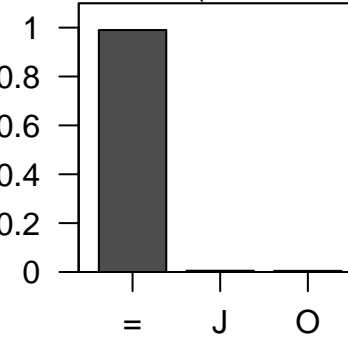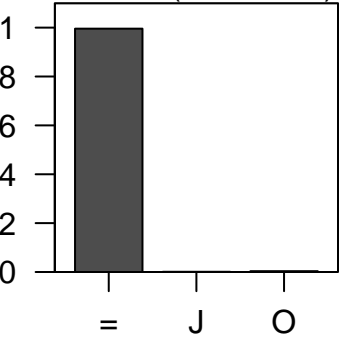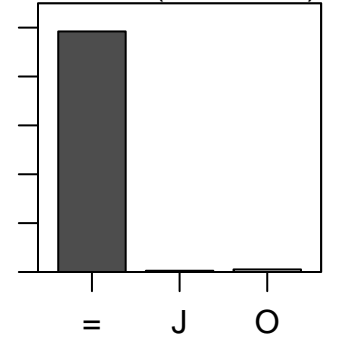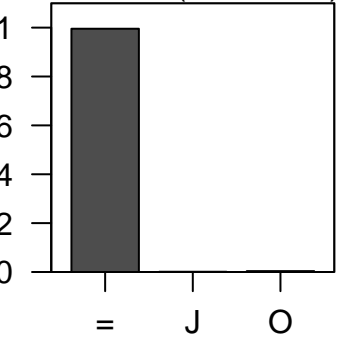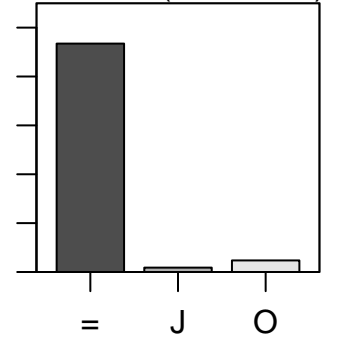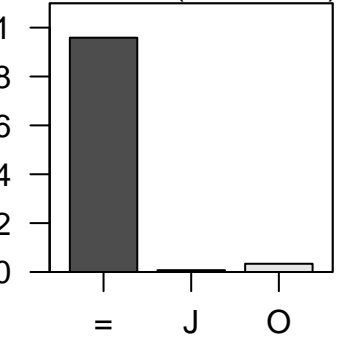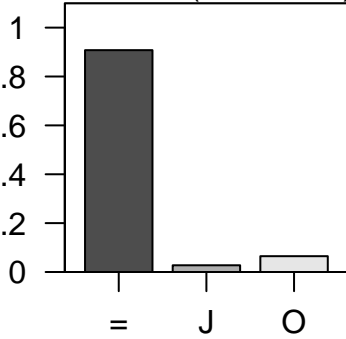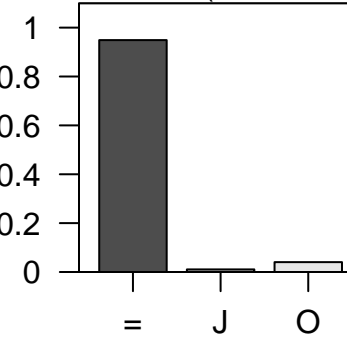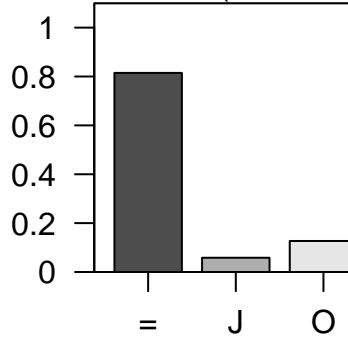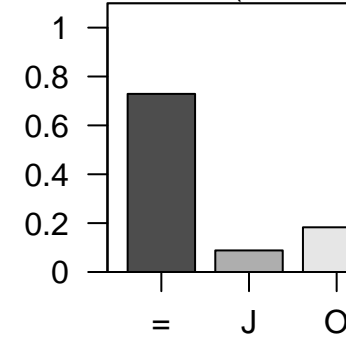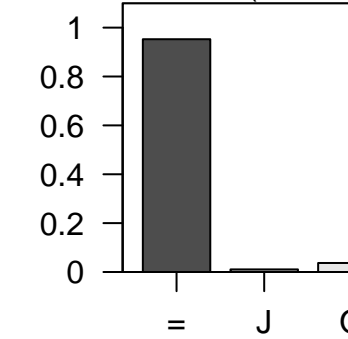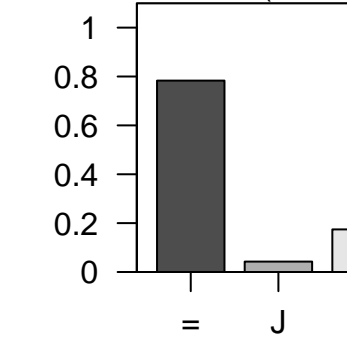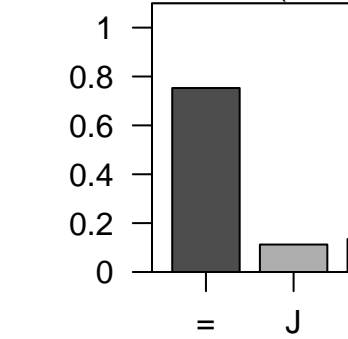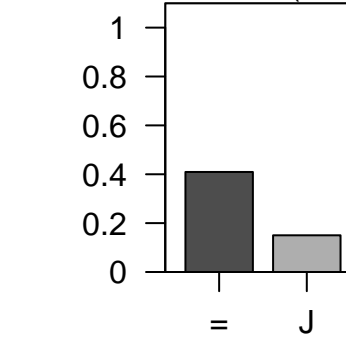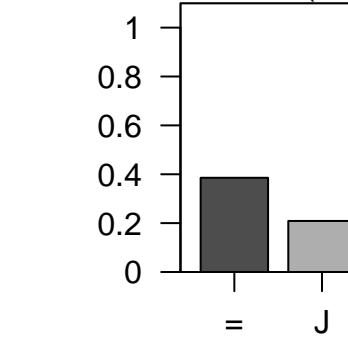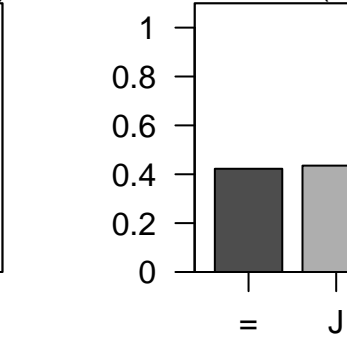

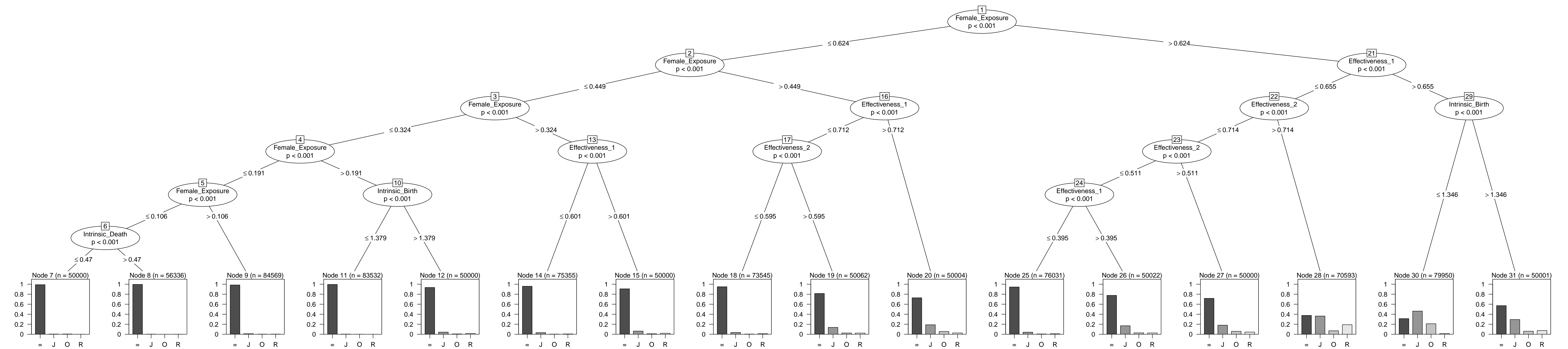

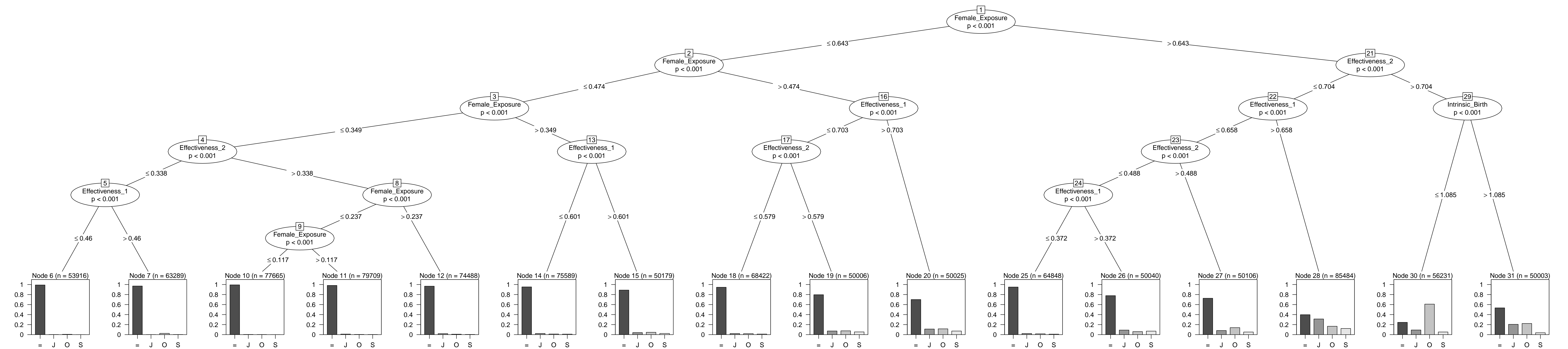

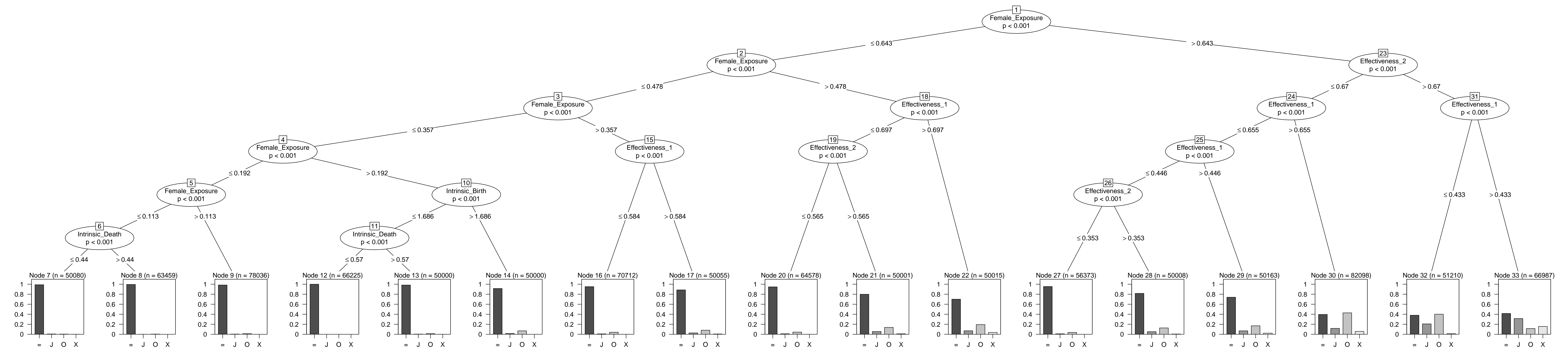

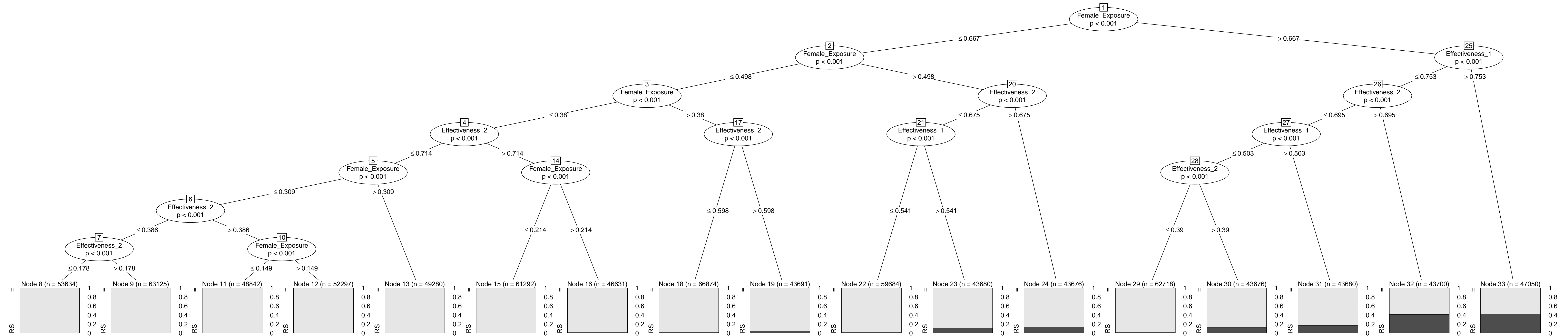

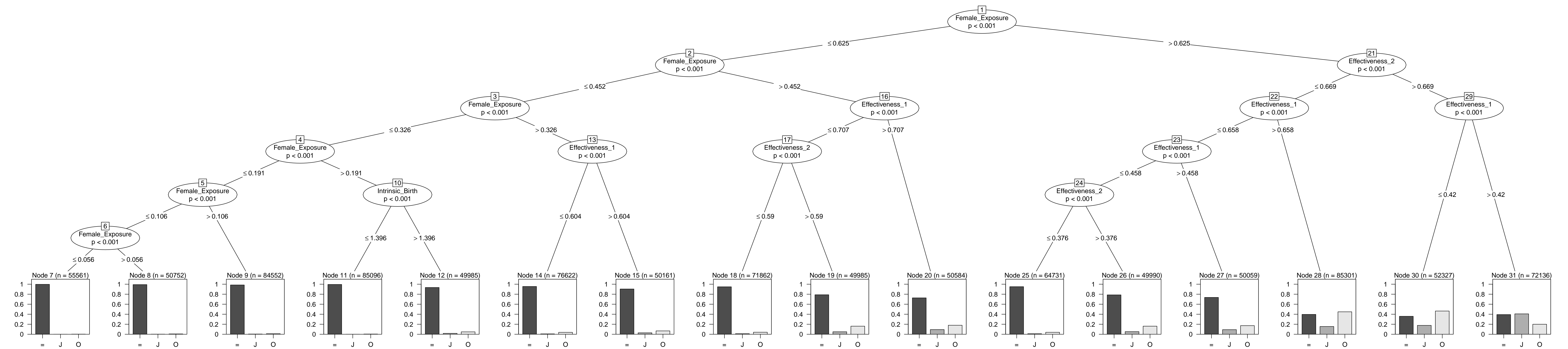

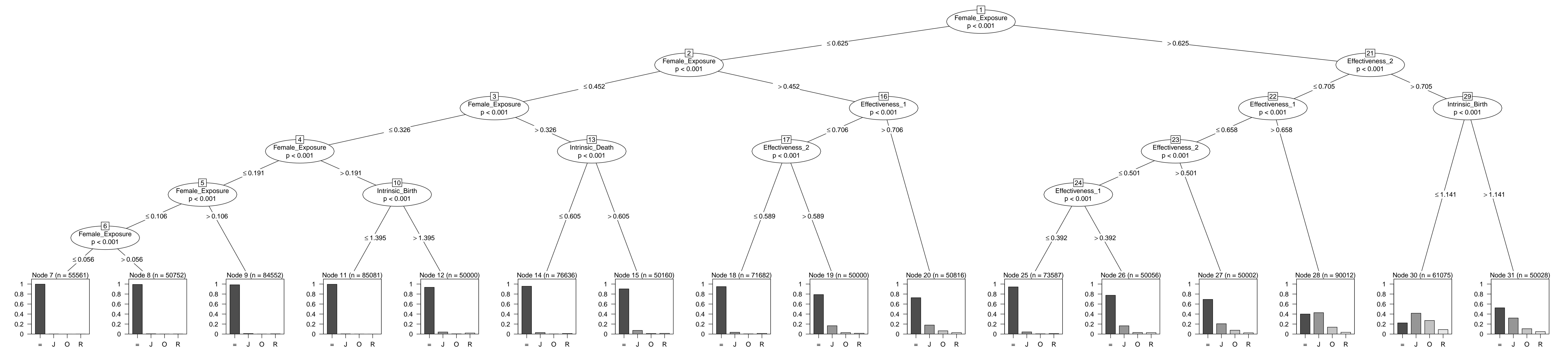

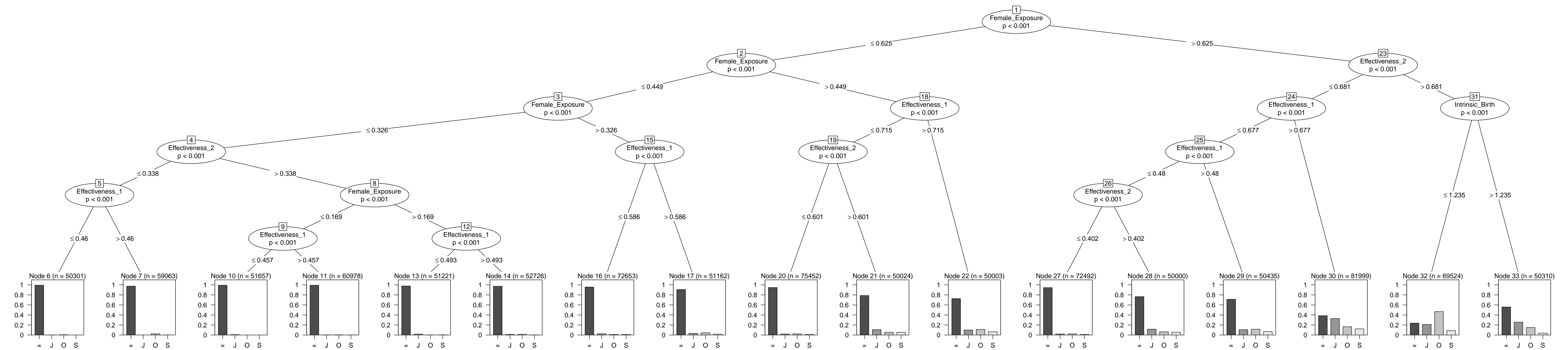

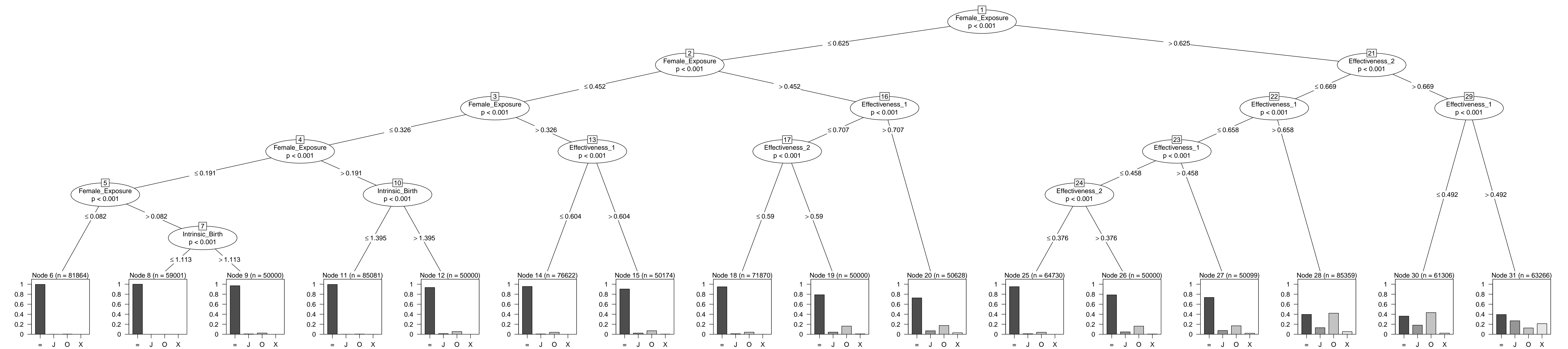

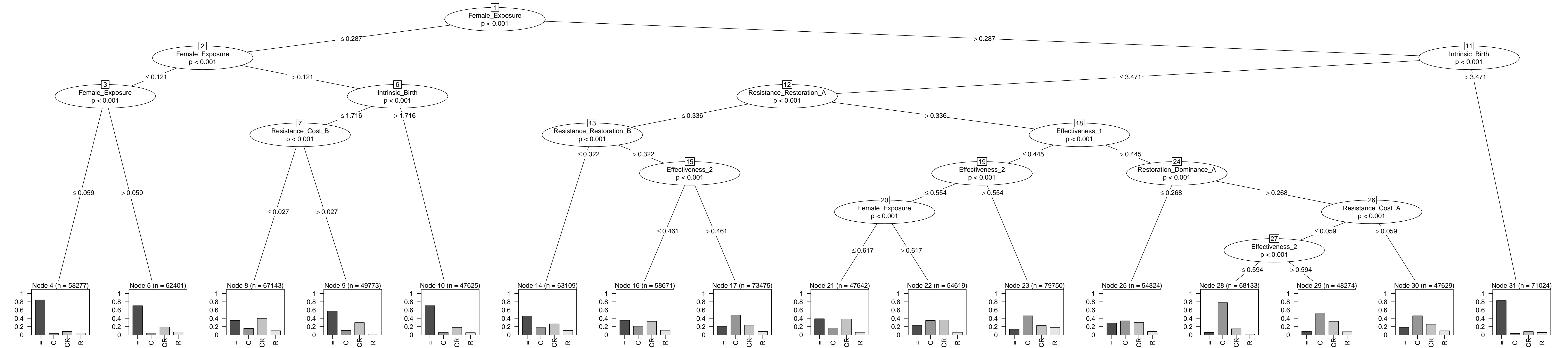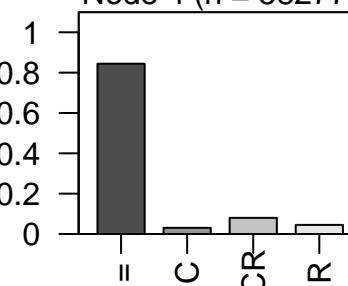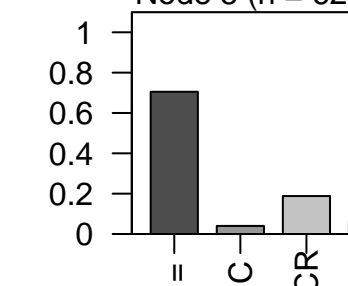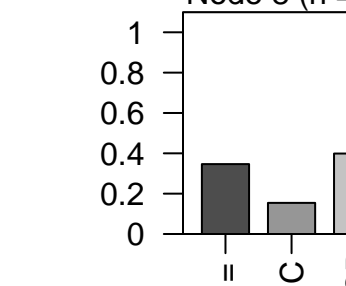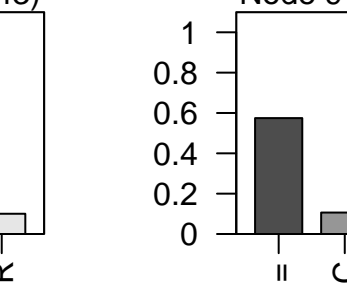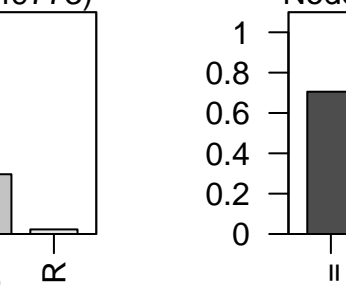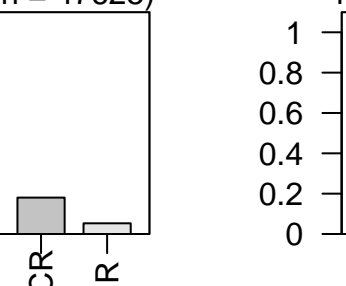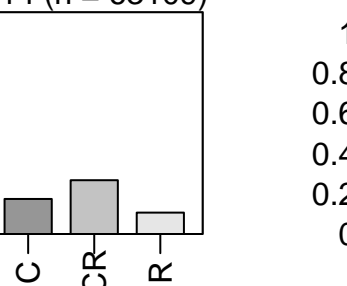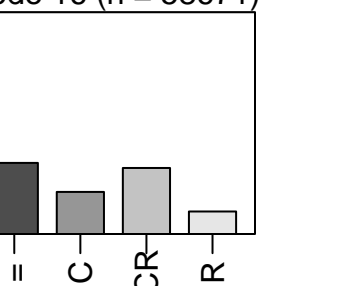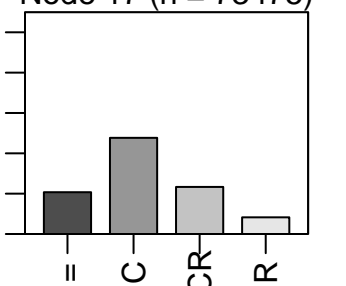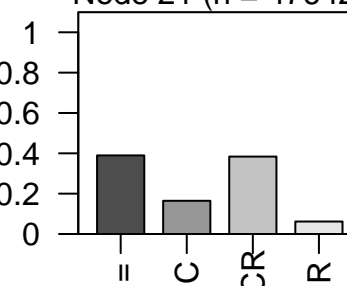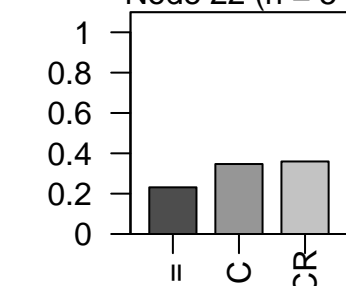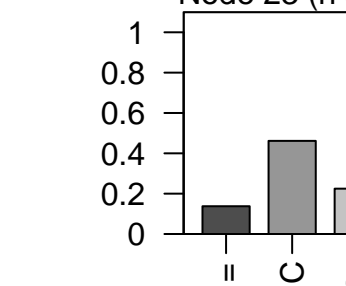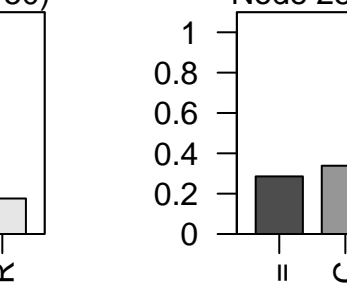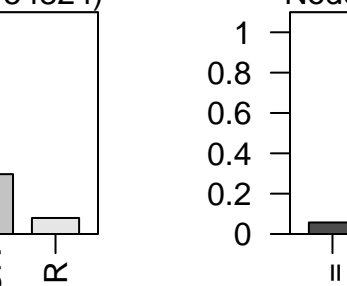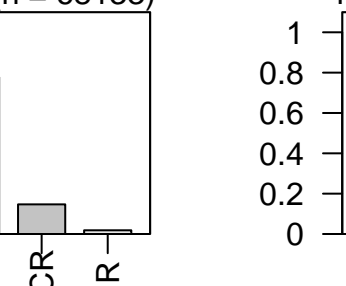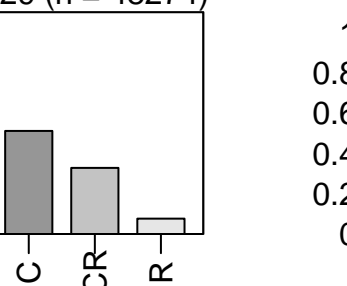

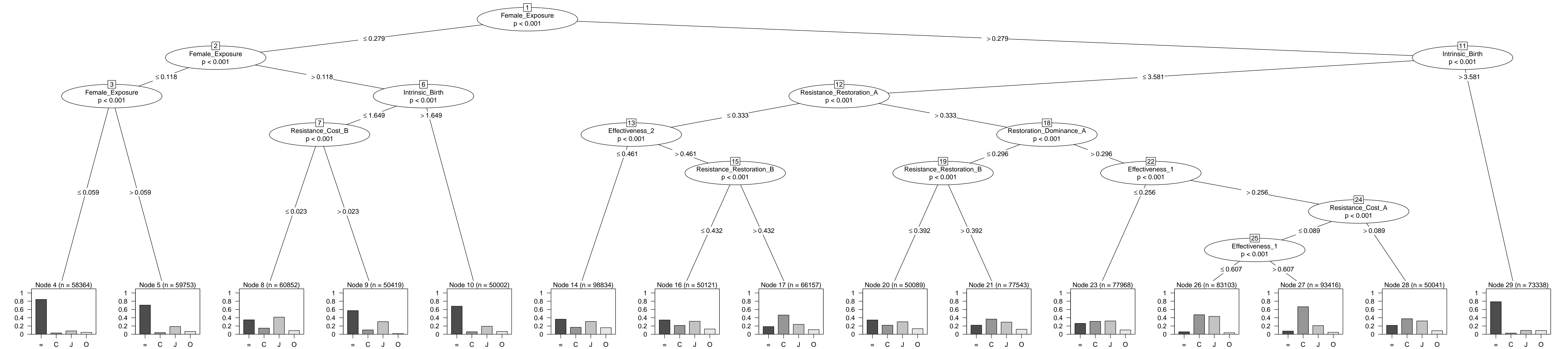

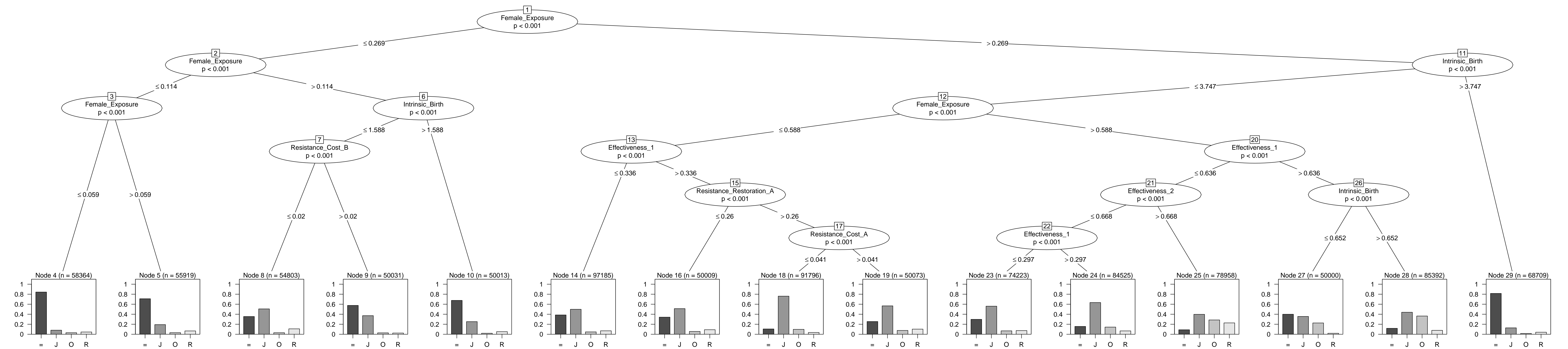

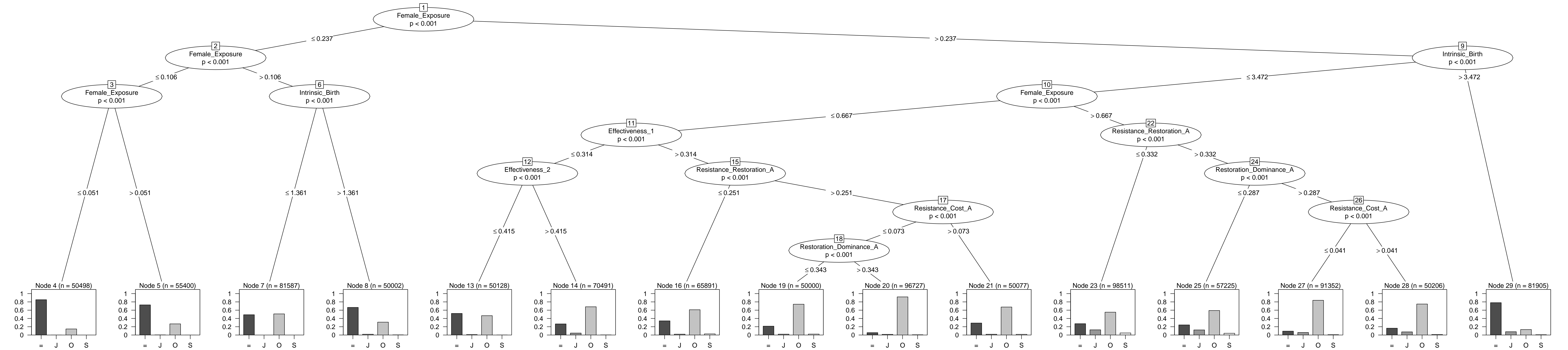

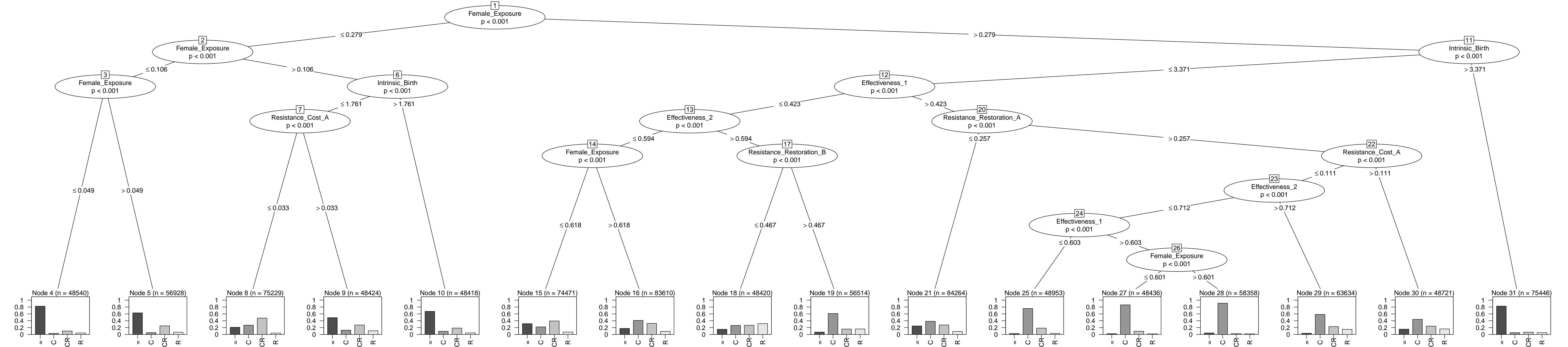

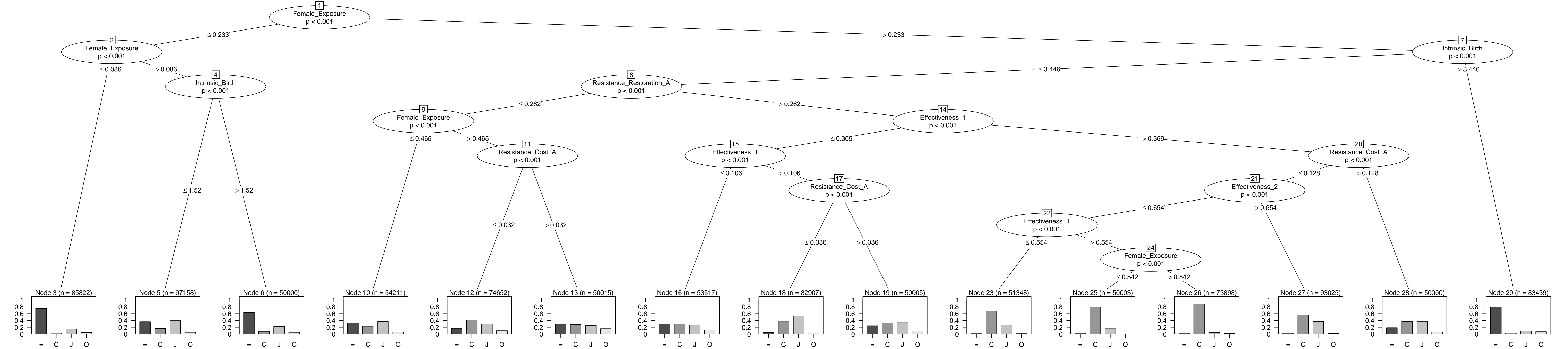

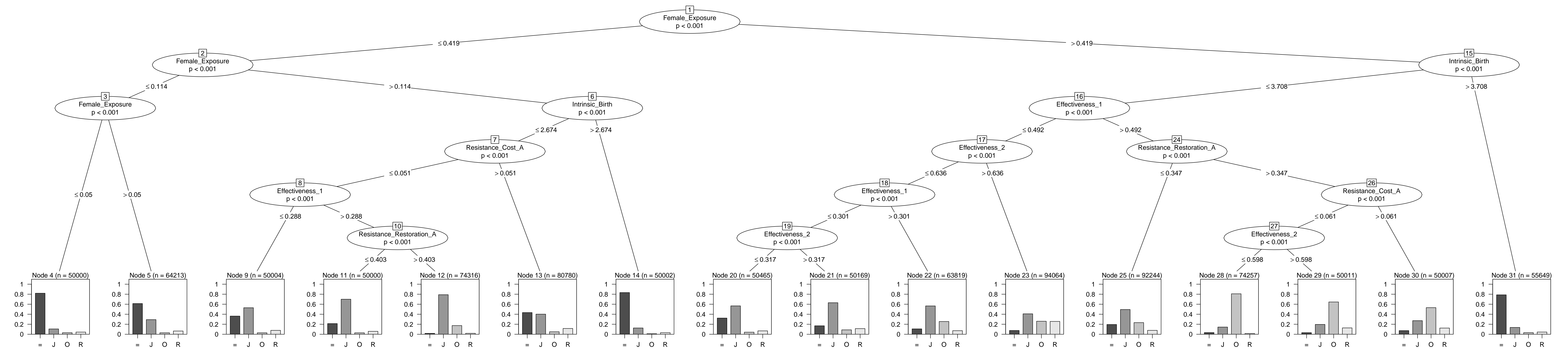

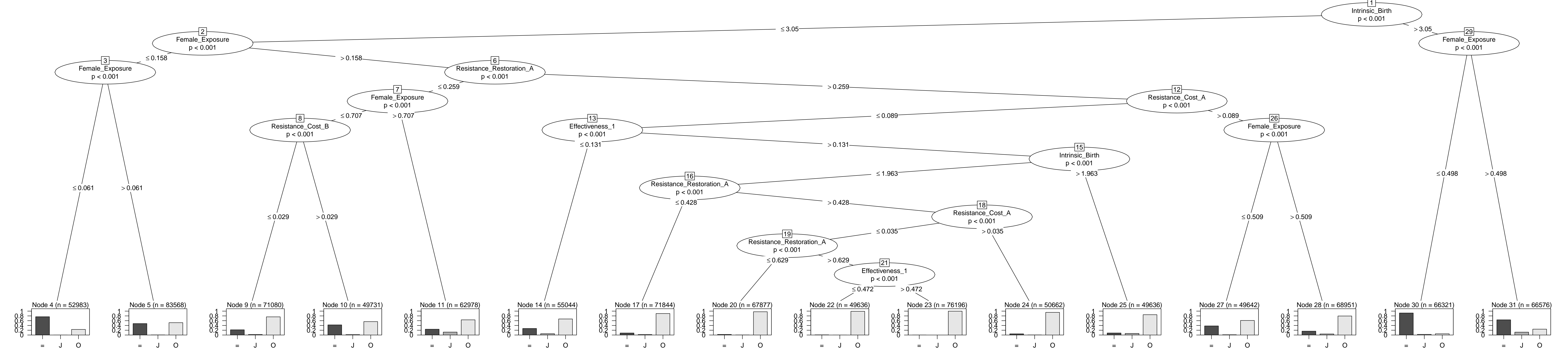

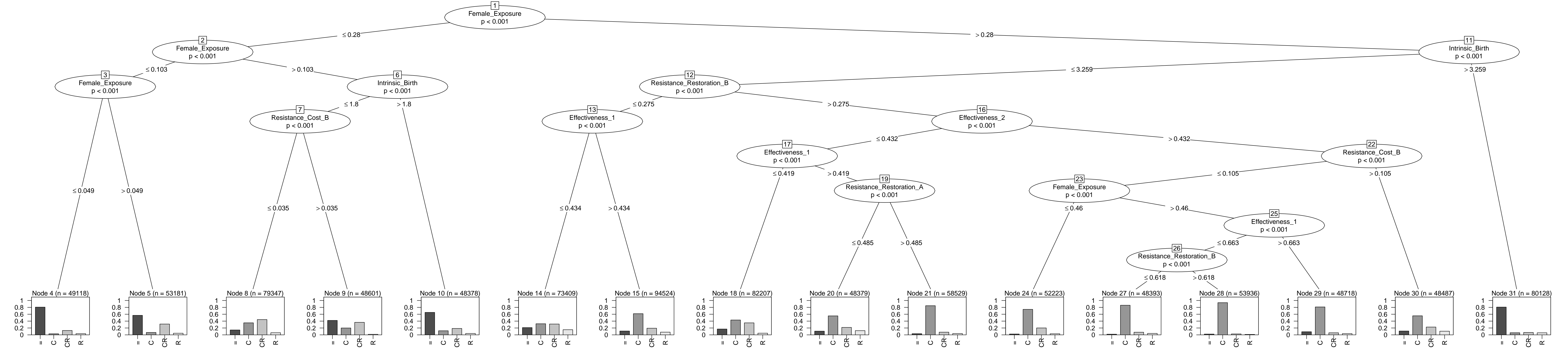

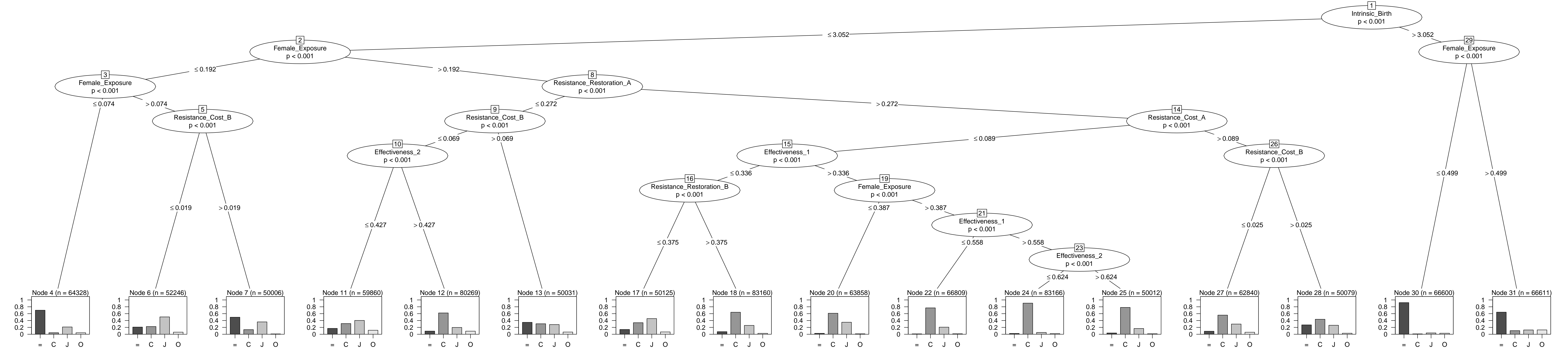

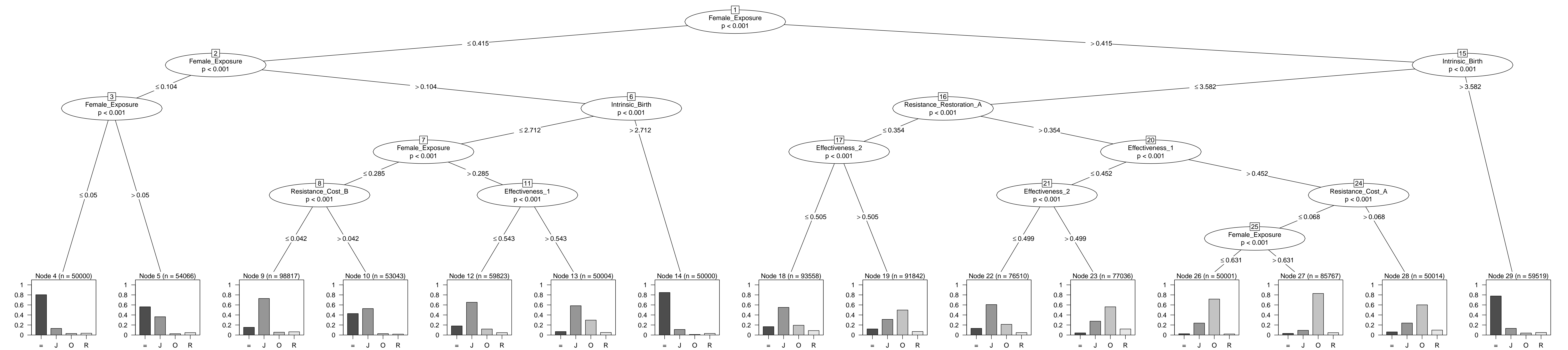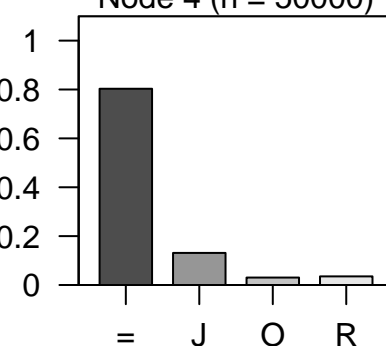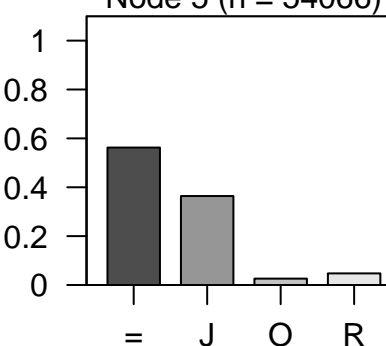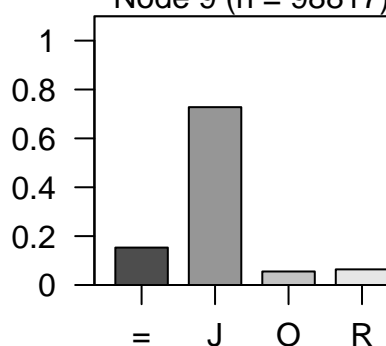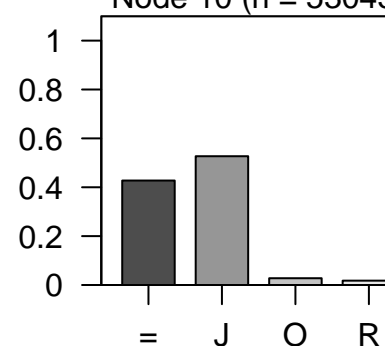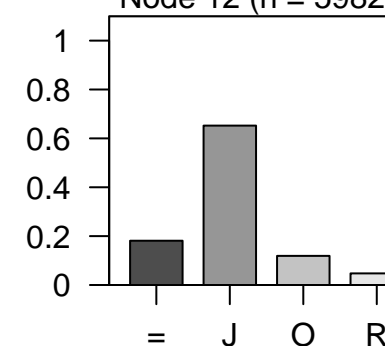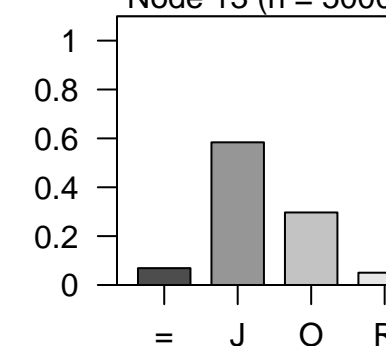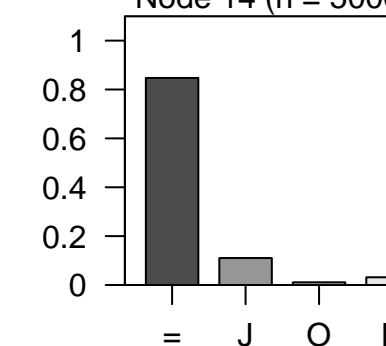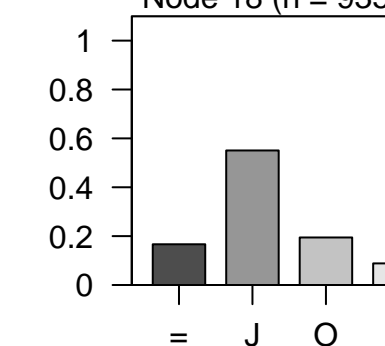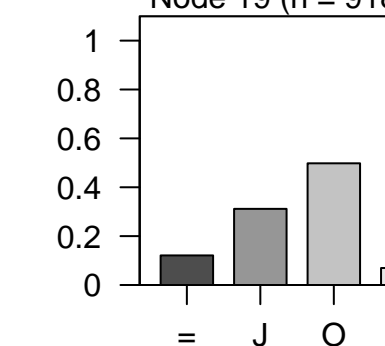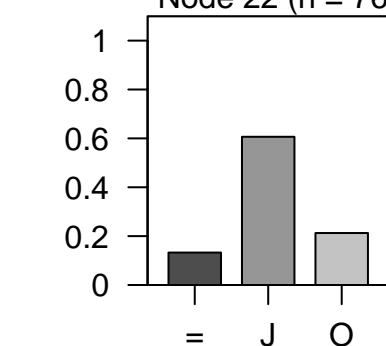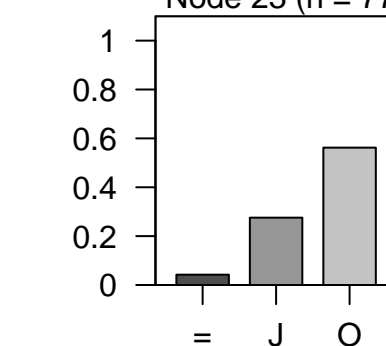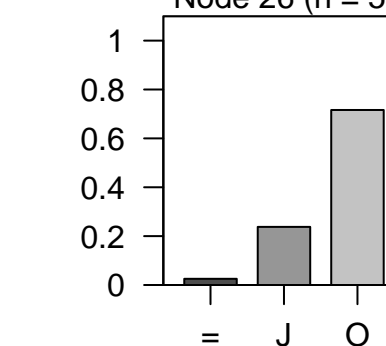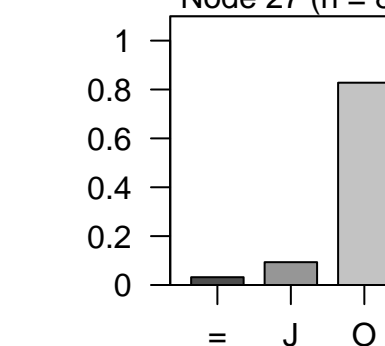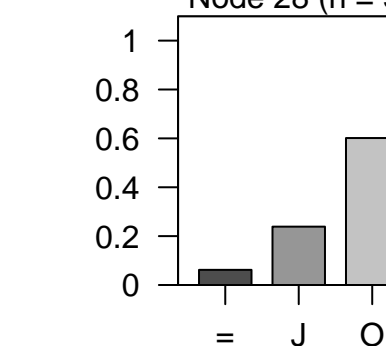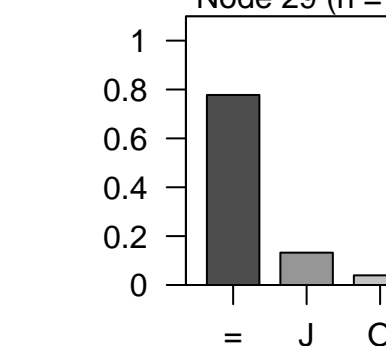

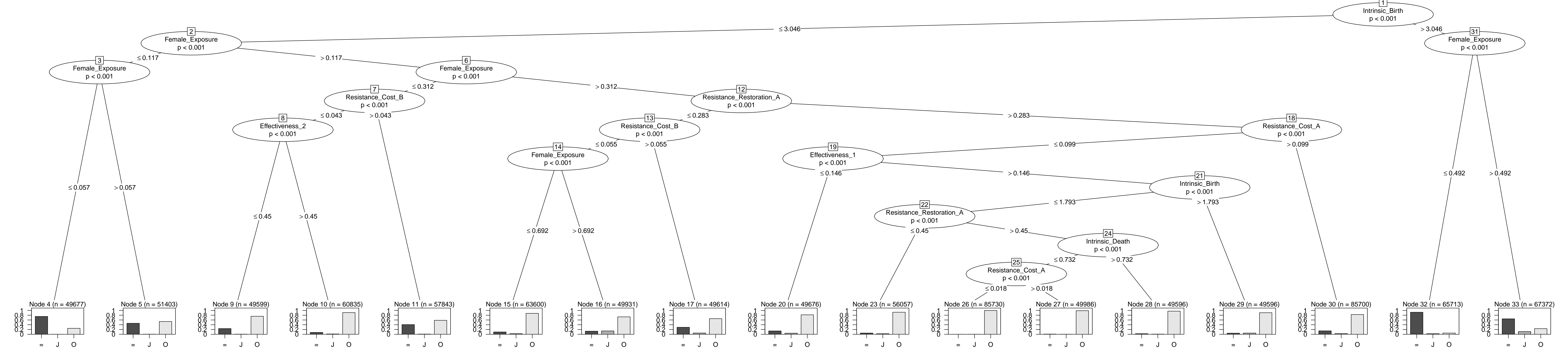

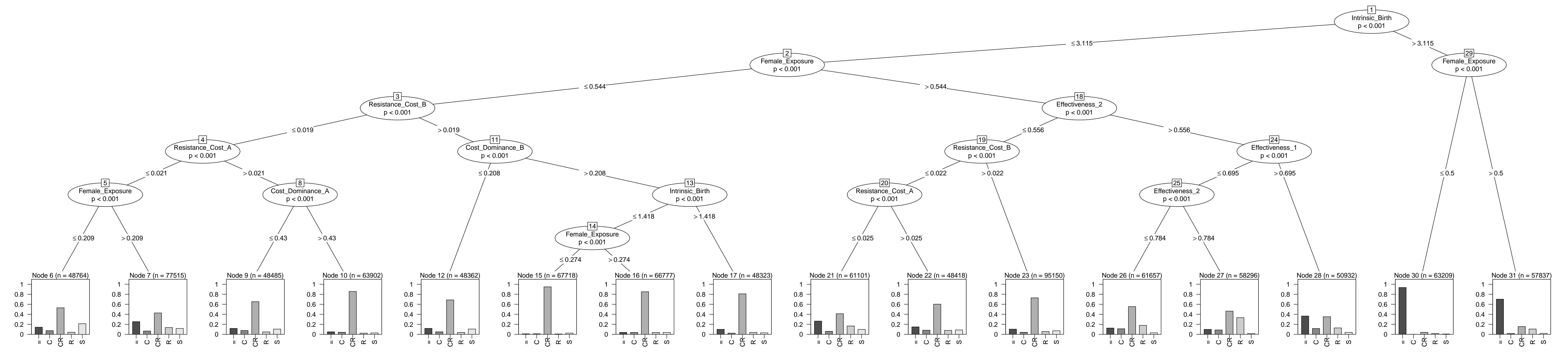

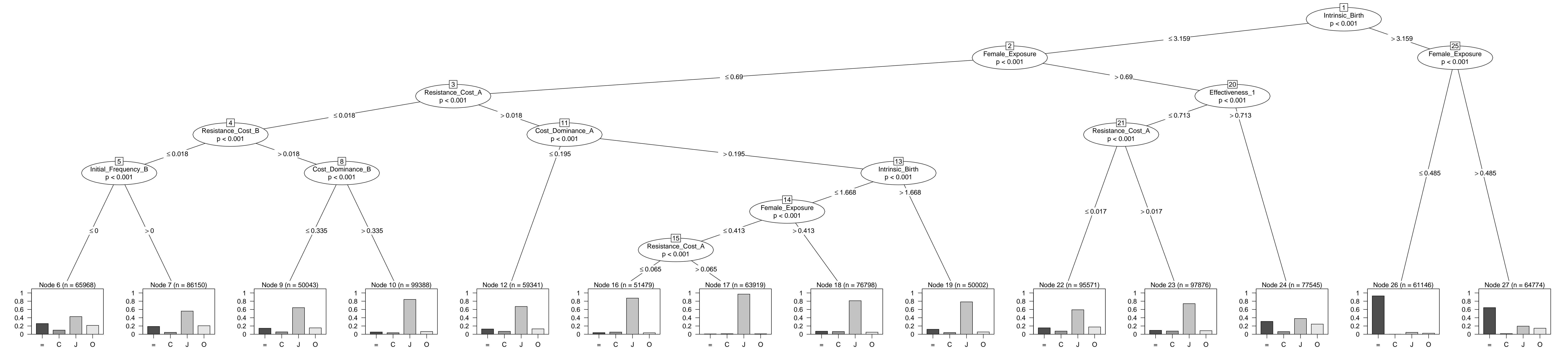

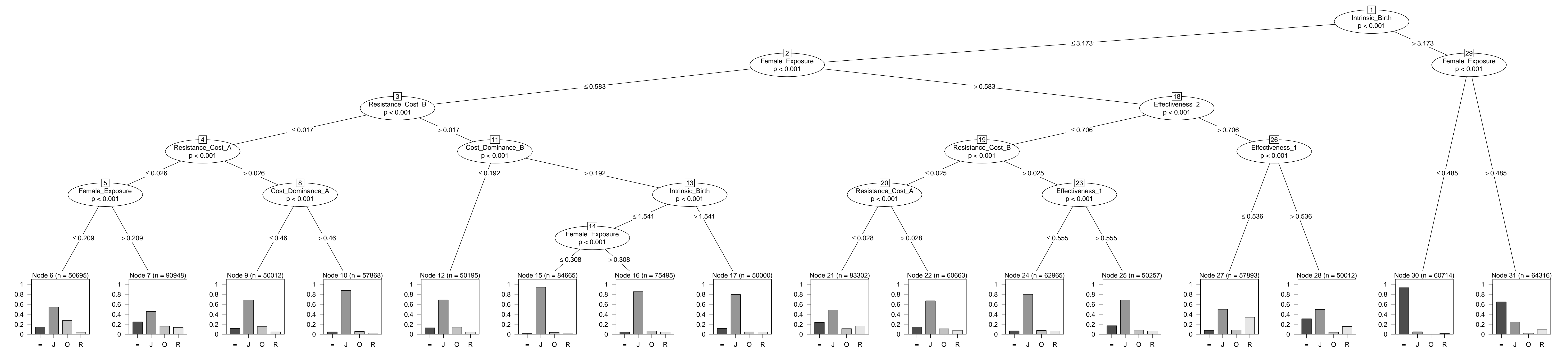

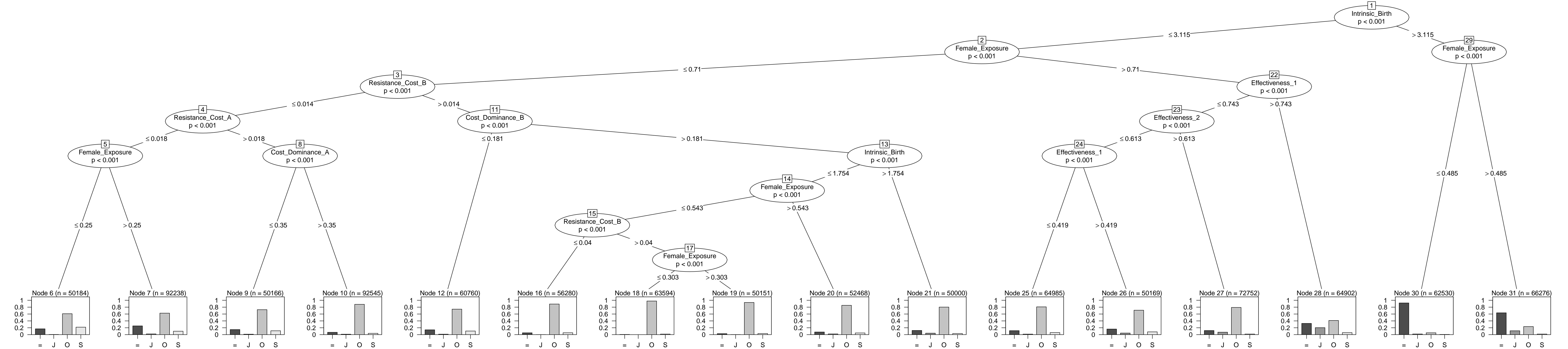

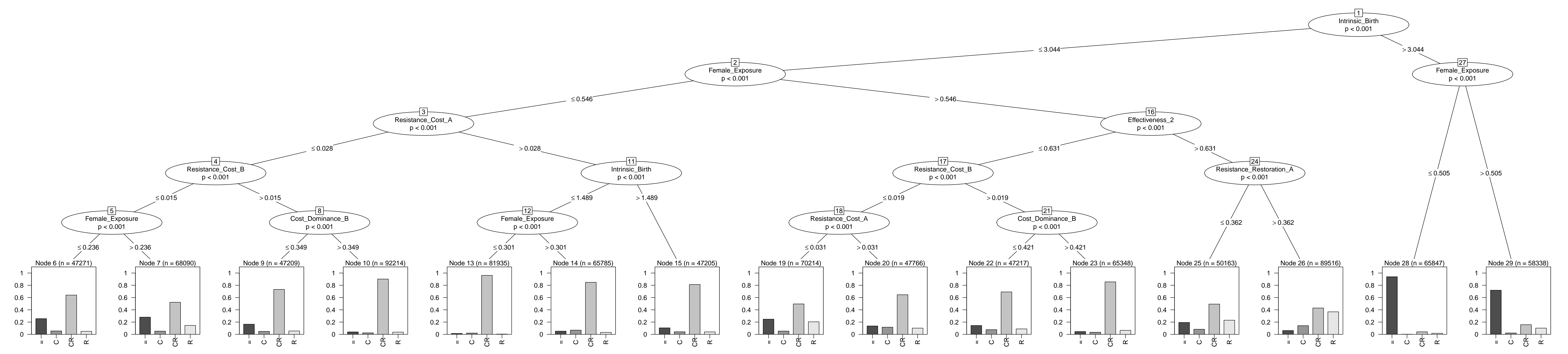

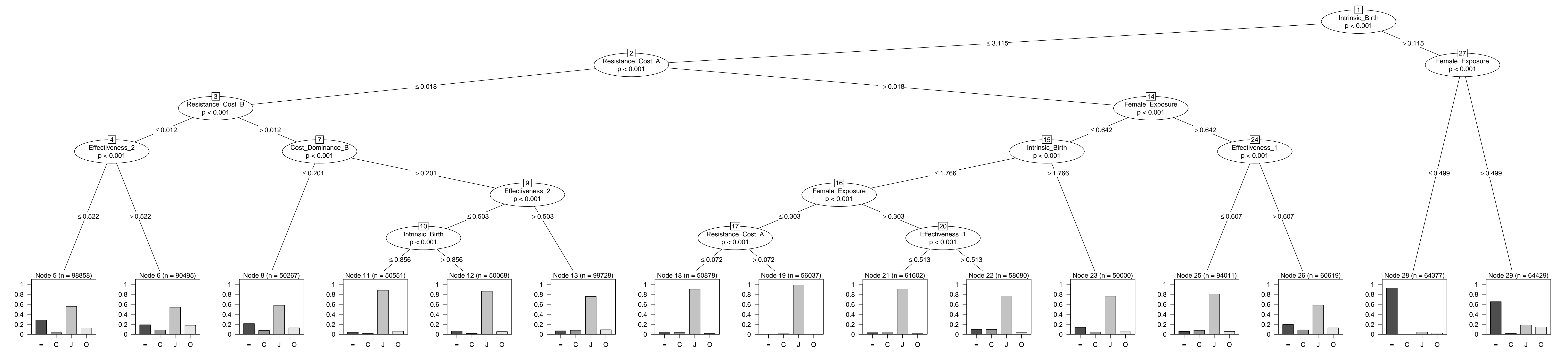

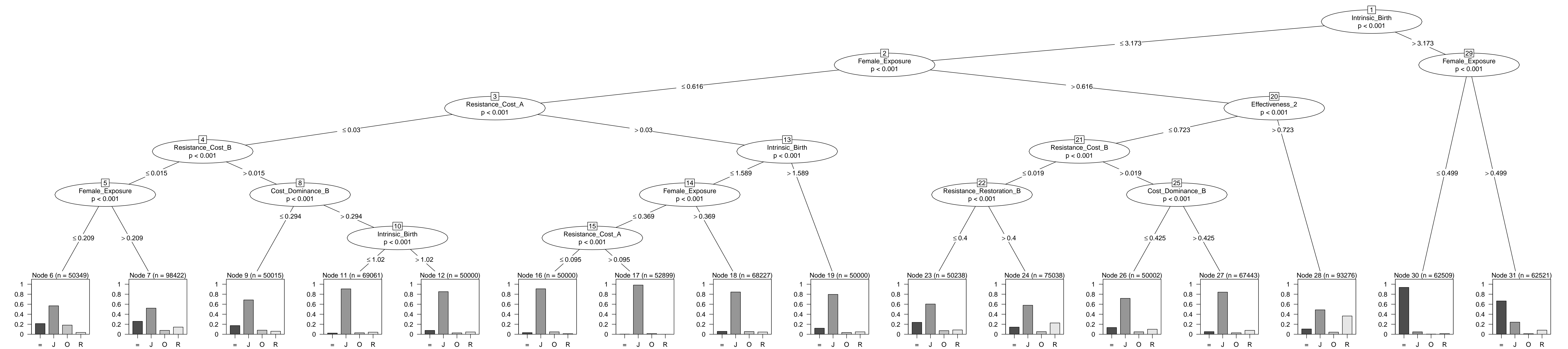

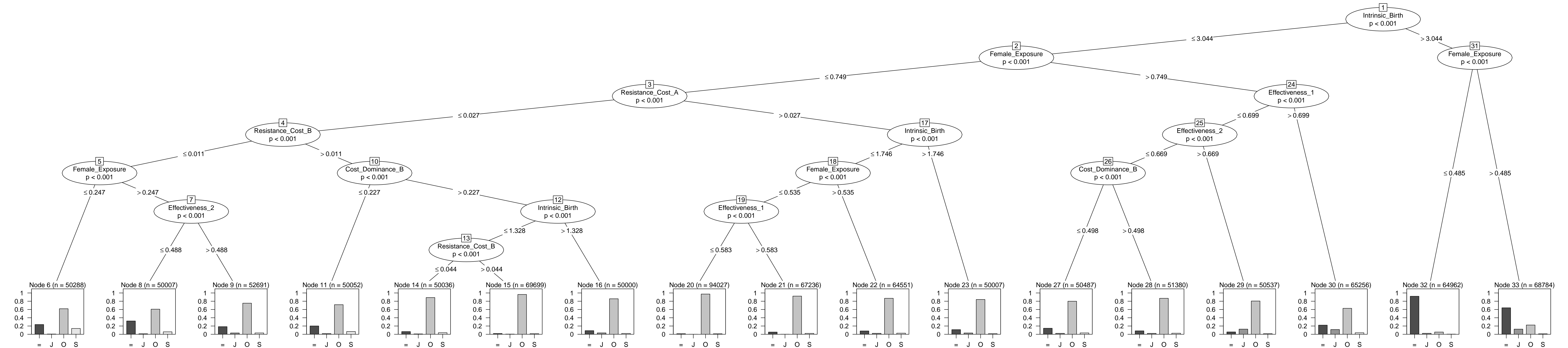

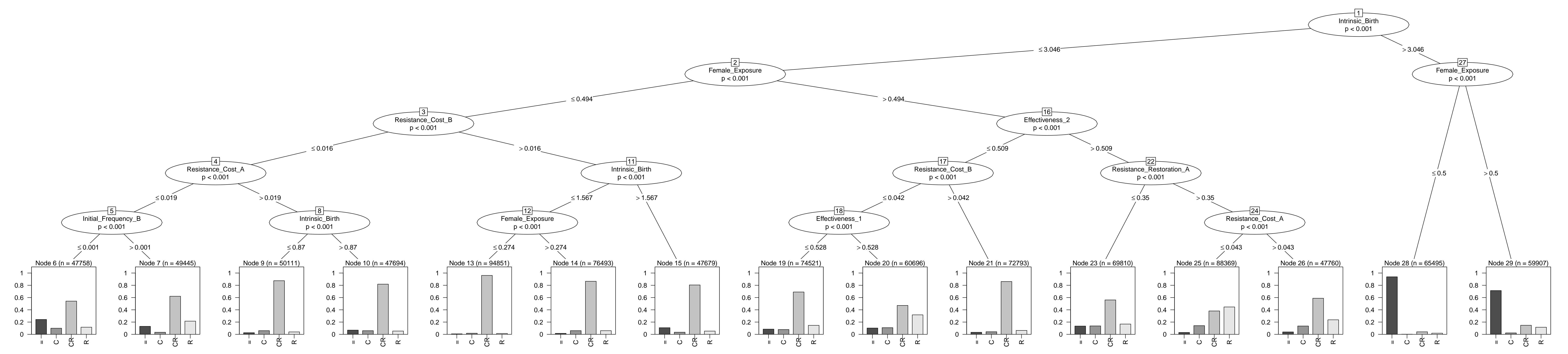

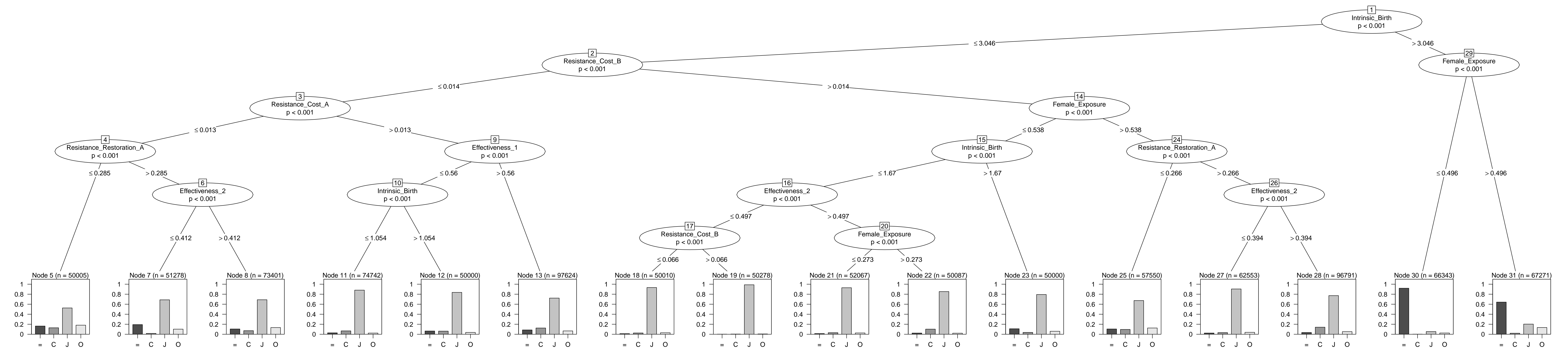

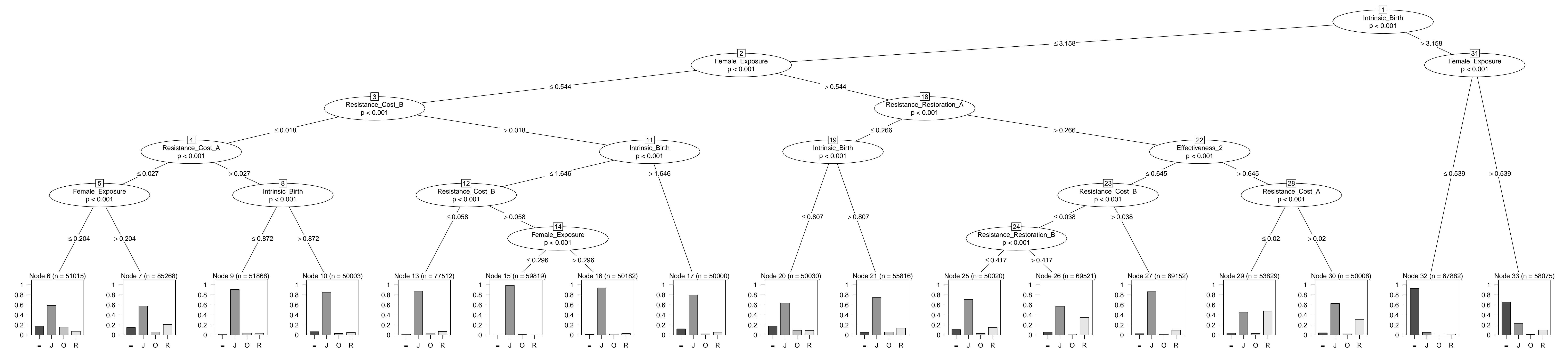

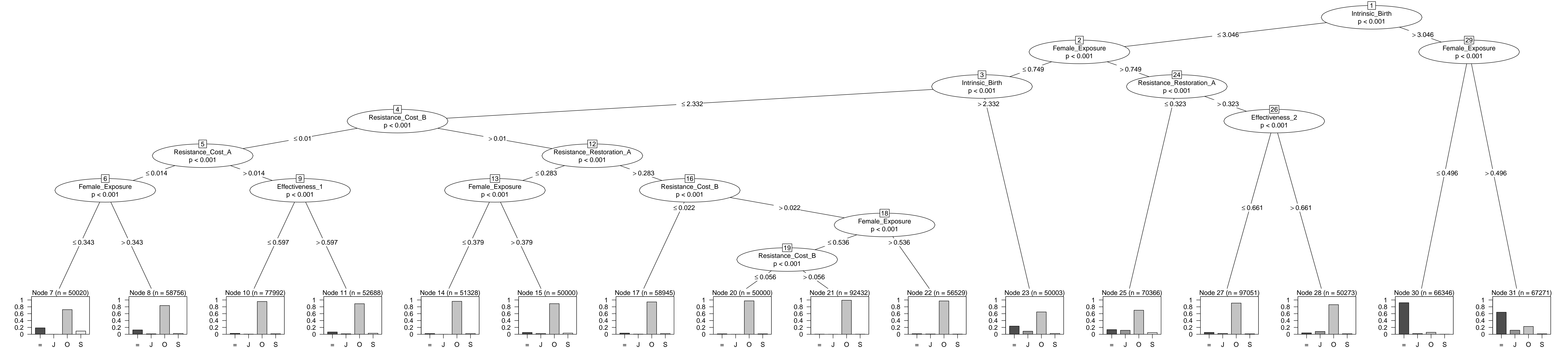

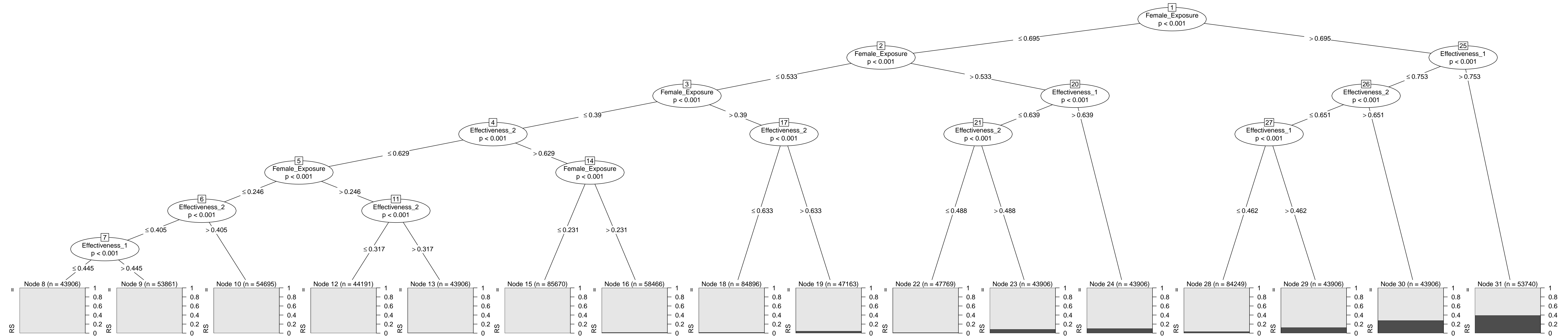

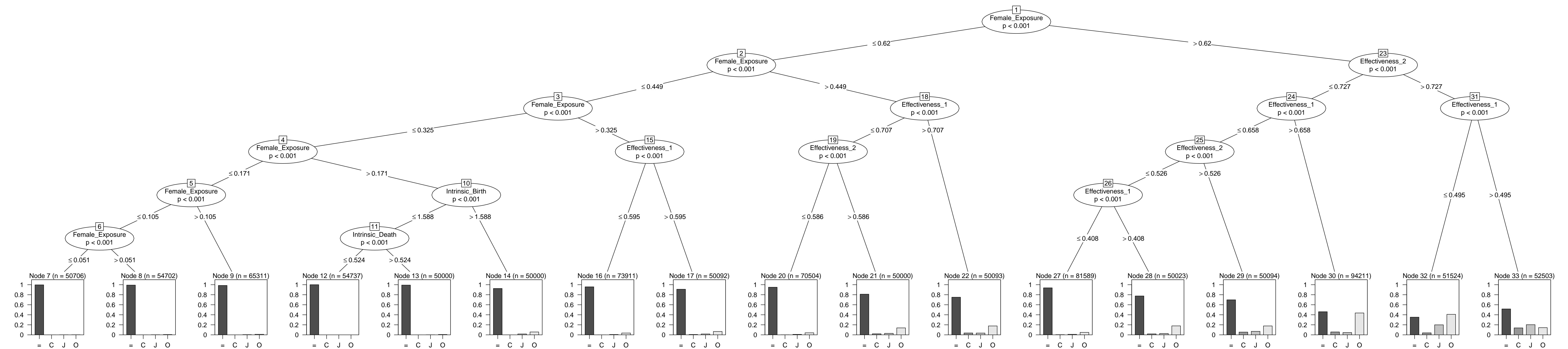

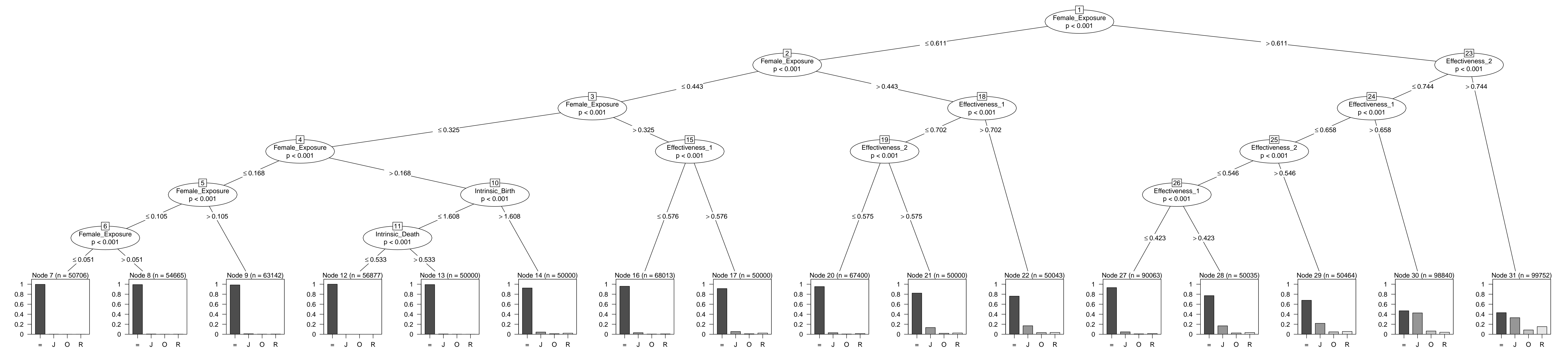

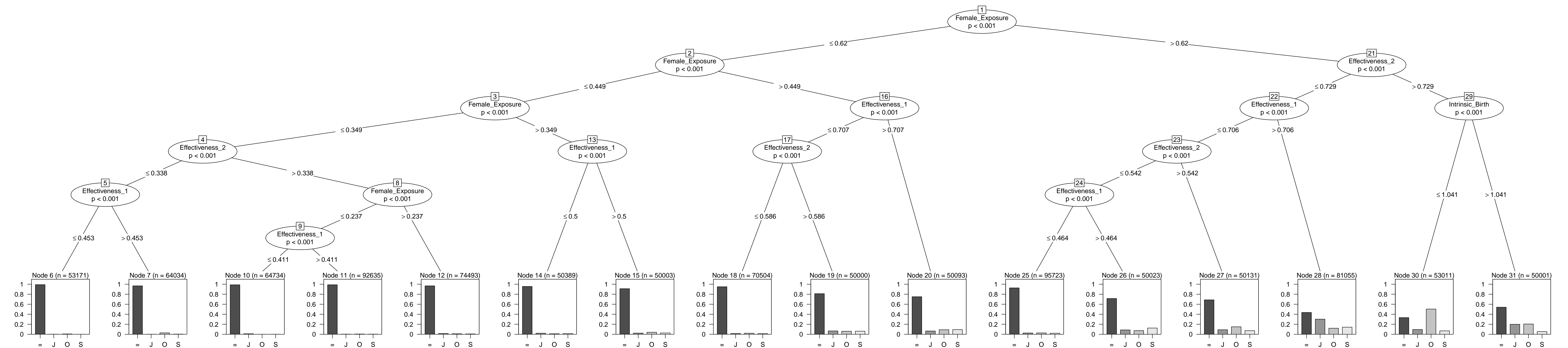

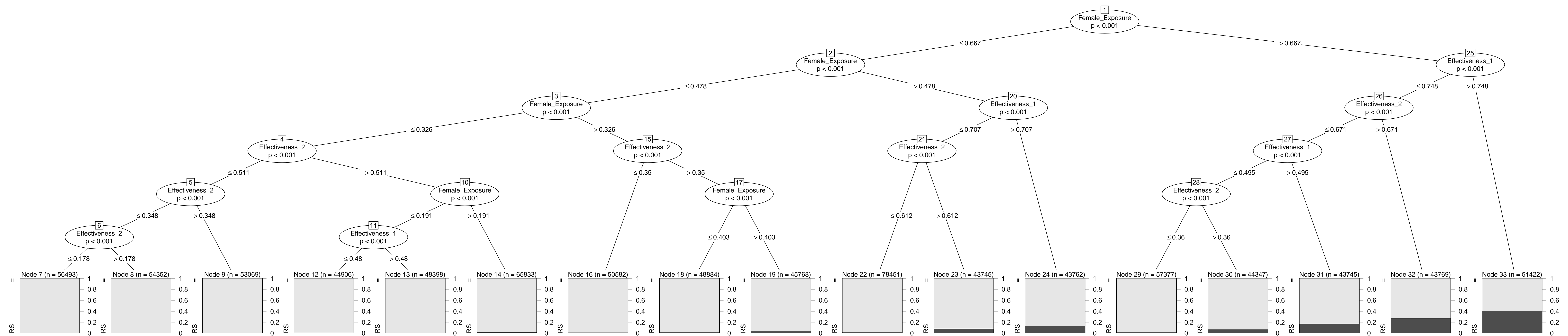

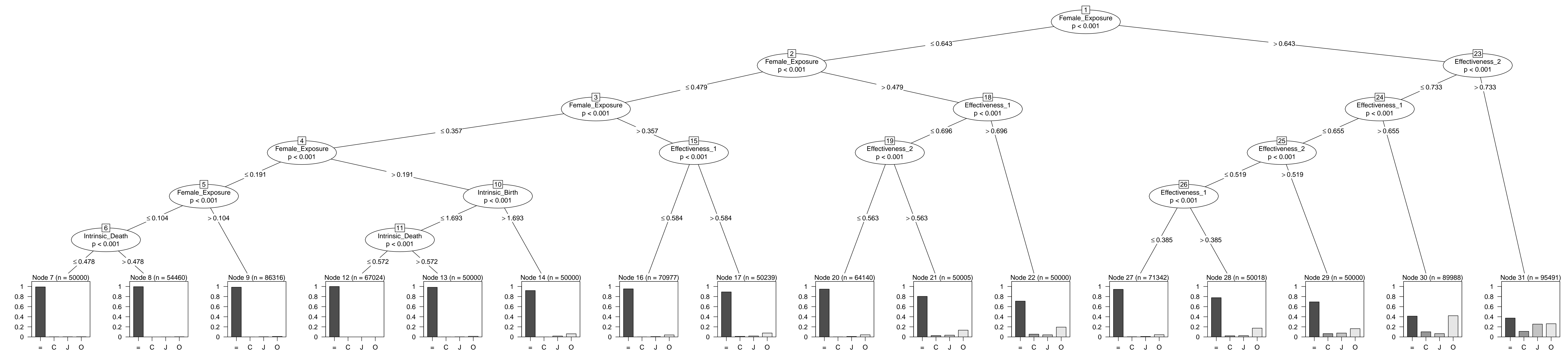

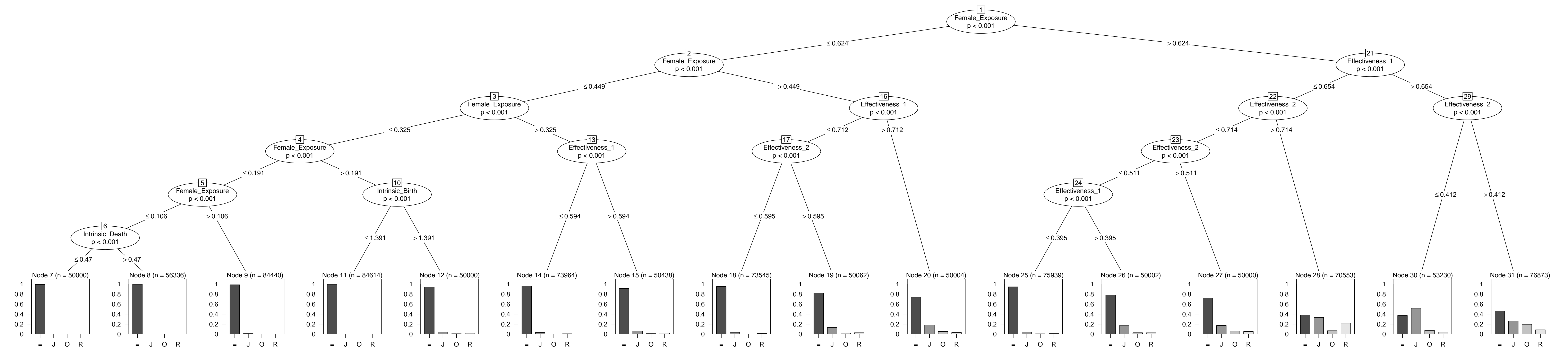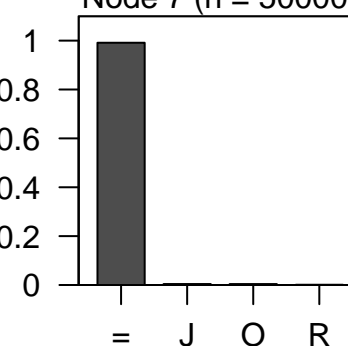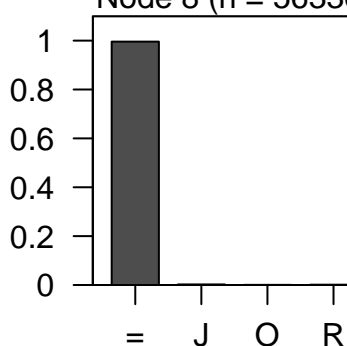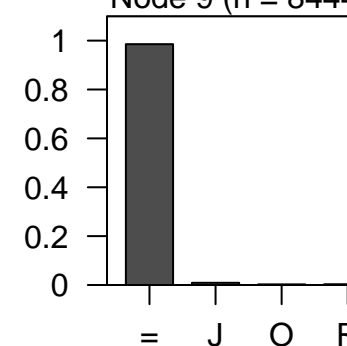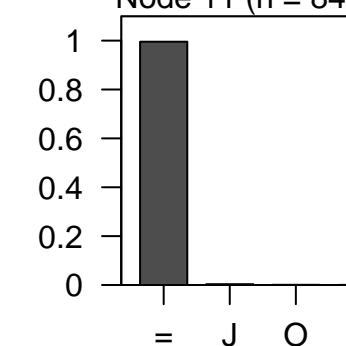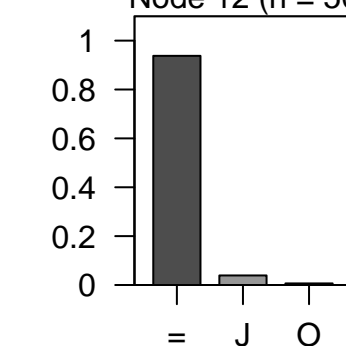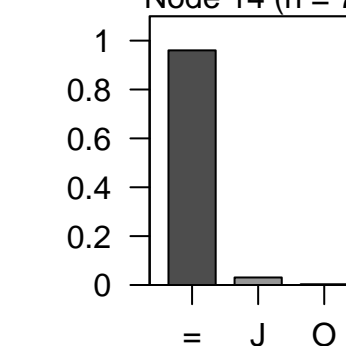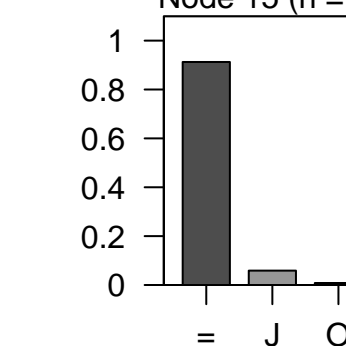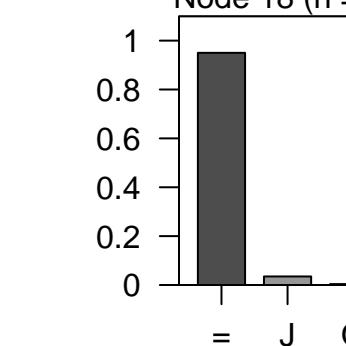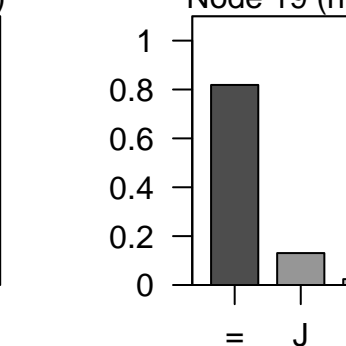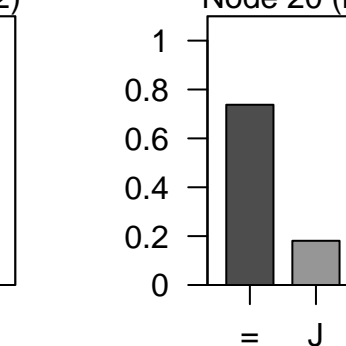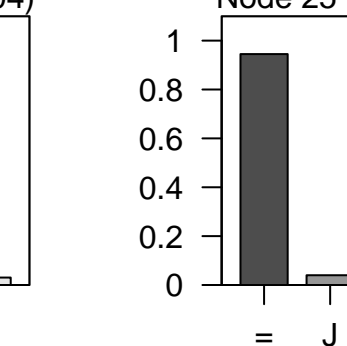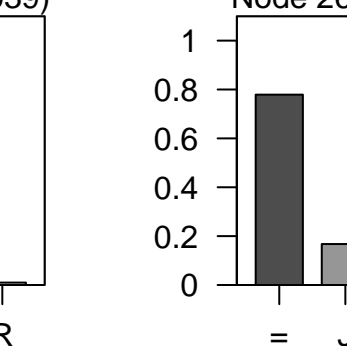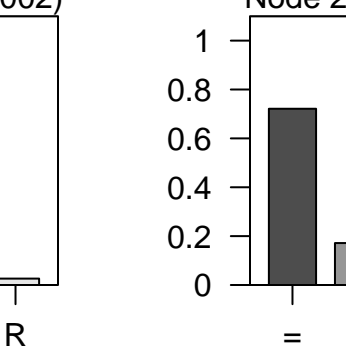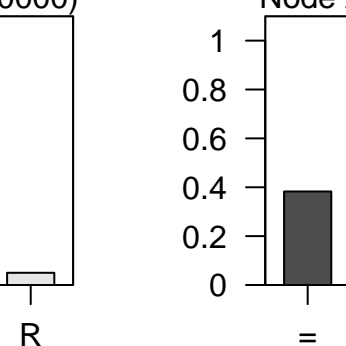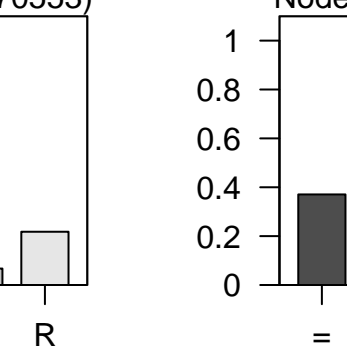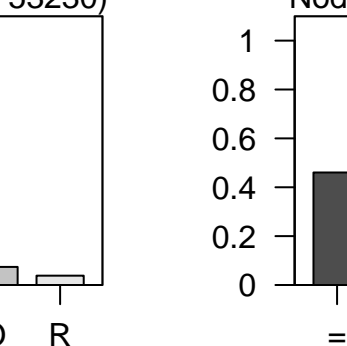

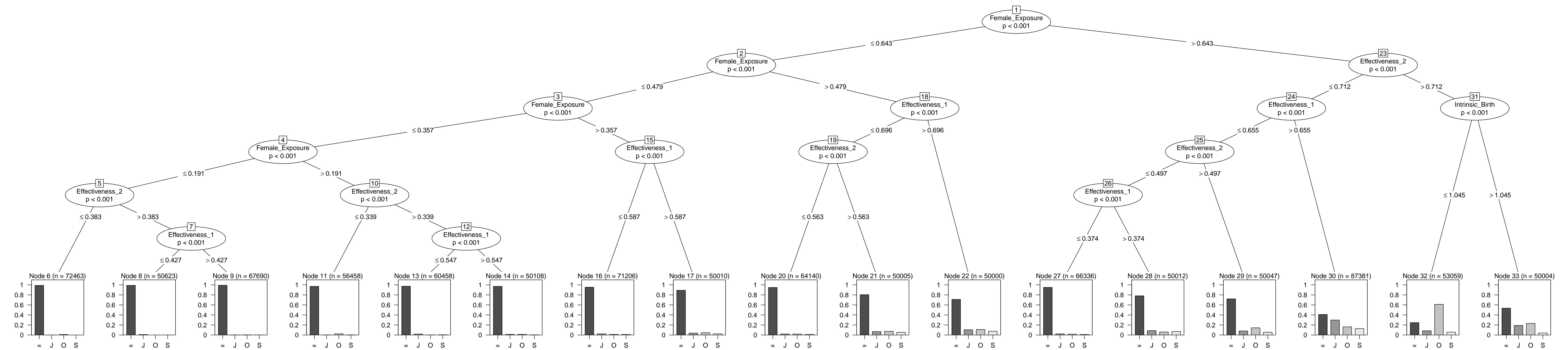

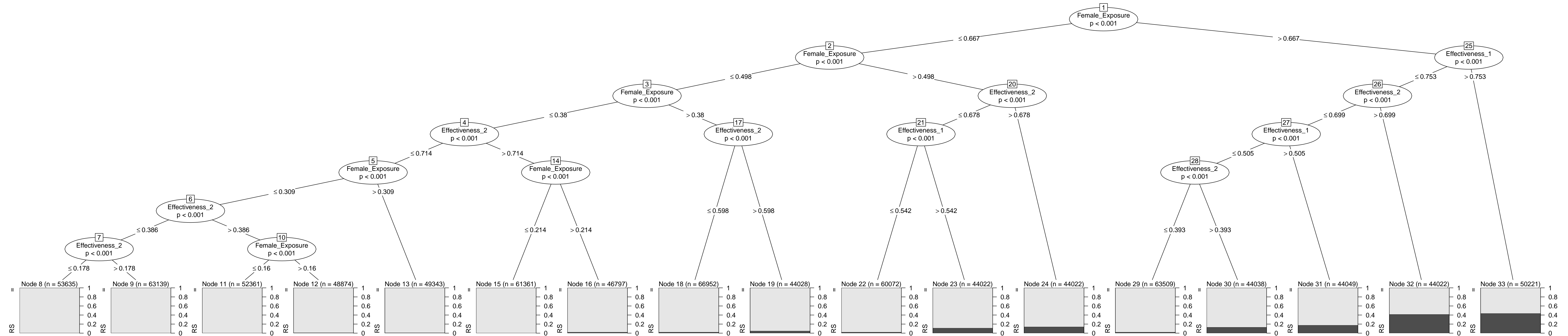

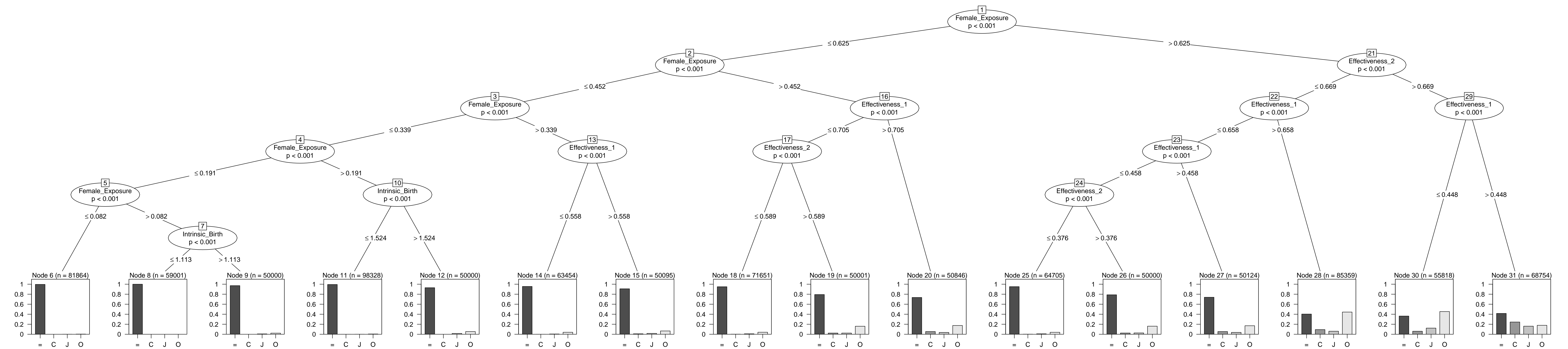

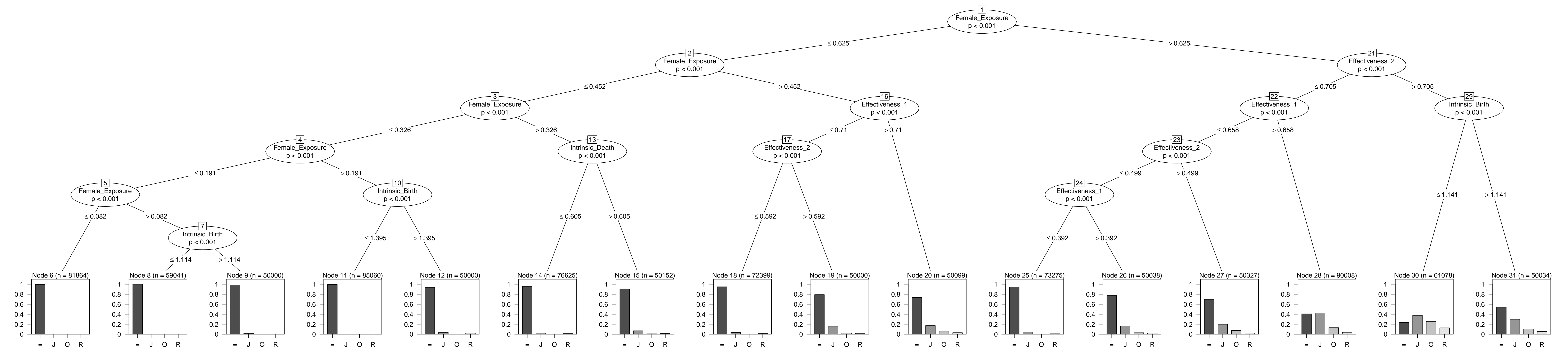

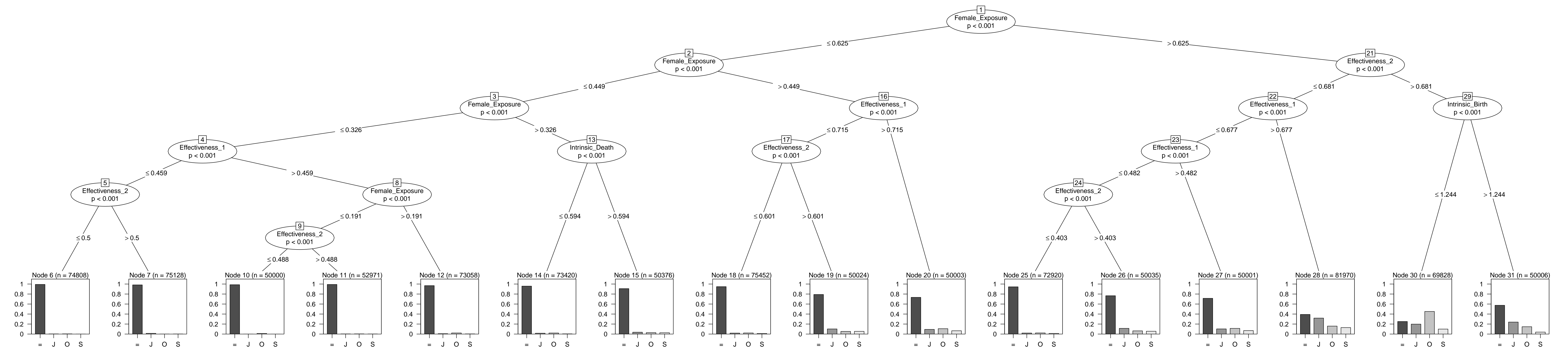

Supplement: Supplementary file 3 — Additional file 3. Supporting conditional inference trees. [file 12936_2022_4083_MOESM3_ESM.pdf]
